# Syntaxin11 deficiency inhibits CRAC channel priming to suppress cytotoxicity and gene expression in T lymphocytes independent of membrane trafficking

**DOI:** 10.7554/eLife.111335

**Published:** 2026-10-19

**Authors:** Sritama Datta, Abhikarsh Gupta, Kunal M Jagetiya, Resmi Bera, Vikas R Tiwari, Megumi Yamashita, Atharva R Yande, Abdul Rishad, Vishal Malik, Sreejith Raran-Kurussi, Sandra Ammann, Mohammad Shahrooei, Kalyaneswar Mandal, Ramanathan Sowdhamini, Murali Prakriya, Adish Dani, Monika Vig

**Affiliations:** 1 Tata Institute of Fundamental Research Hyderabad India; 2 National Centre for Biological Sciences Bangalore India; 3 Northwestern University, Feinberg School of Medicine Chicago United States; 4 Institute for Immunodeficiency, University of Freiburg Freiburg im Breisgau Germany; 5 Institute for Transfusion Medicine and Gene Therapy, University of Freiburg Freiburg im Breisgau Germany; 6 Dr. Shahrooei Lab Tehran Iran; National Institute of Neurological Disorders and Stroke United States; National Institute of Neurological Disorders and Stroke United States

**Keywords:** CRAC channel, Orai, T lymphocytes, Syntaxin, SNARE, FHLH, Human

## Abstract

Mutations in Syntaxin11 (STX11), a Q-SNARE, result in a fatal immune disorder known as familial hemophagocytic lymphohistiocytosis 4 (FHLH4) in human patients. A key diagnostic feature of FHLH4 is defective T and natural killer cell cytotoxicity. Here, we show that STX11 directly binds and regulates Orai1, the pore-forming subunit of calcium release-activated calcium (CRAC) channels. CRAC channels enable store-operated calcium entry (SOCE) from the extracellular space and are crucial for granule exocytosis and nuclear factor of activated T cells (NFAT)-dependent gene expression in activated lymphocytes. STX11 depletion strongly inhibited SOCE, CRAC currents, NFAT activation, interleukin-2 gene expression, and degranulation in FHLH4 patient T lymphocytes and cell lines without affecting membrane trafficking. Remarkably, defects of cytolytic granule exocytosis, as well as interleukin-2 expression, could be reversed by ionomycin in patient T lymphocytes, and a constitutively active, H134S, mutant of Orai1 rescued calcium entry in STX11-depleted cells. Further analyses showed that STX11 ‘primes’ Orai1 for optimal on-site multimeric assembly, which was Stim independent but required for gating. Priming of ion channel pore subunits is, therefore, a primary function of specific SNAREs which may have preceded their role in membrane trafficking and vesicle fusion.

## Introduction

Familial hemophagocytic lymphohistiocytosis 4 (FHLH4) is a life-threatening immune disease caused due to mutations of STX11 (Zhang et al., 1993). The underlying cause of FHLH is reduced cytolytic activity of T and natural killer (NK) cells (D’Orlando et al., 2013), which renders patients susceptible to recurrent infections. Patients suffer from high fever, severe lymphopenia in early infancy, and succumb to disease by adolescence, unless given bone marrow transplants. STX11 is highly expressed in almost all cells of the immune system and, being a Q-SNARE, is associated with exocytosis of vesicles, by default. Intriguingly, FHLH4 patients also manifest cytokine storm, which cannot be reconciled with a presumed general defect in membrane trafficking and vesicle fusion.

Most eukaryotic cells have a limited amount of calcium sequestered inside intracellular stores, the largest of which is the endoplasmic reticulum (ER). Signaling from cell surface receptors induces the release of stored calcium which, in turn, activates store-operated calcium entry (SOCE) to replenish stores, sustain signaling, and drive other calcium-dependent cellular processes (Clapham, 2007). Calcium release activated calcium (CRAC) channels are the major conductors of SOCE (Vig and Kinet, 2007). In lymphocytes and mast cells, CRAC currents have been shown to be crucial for cytotoxicity, degranulation, as well as gene expression (Vig et al., 2008; Maul-Pavicic et al., 2011). Working in concert with a number of accessory proteins, Orai (CRACM) multimers conduct SOCE upon functional clustering with Stim proteins in ER-PM junctions (Vig et al., 2006a; Vig et al., 2006b; Miao et al., 2013). Yet, the prevailing thought attributes all structural transitions that result in the activation of CRAC currents to Stim proteins (Prakriya and Lewis, 2015).

Genome-wide RNAi screens, which initially identified Orai (CRACM), have also yielded a wealth of information regarding additional crucial players in SOCE (Vig et al., 2006b; Feske et al., 2006; Zhang et al., 2006). For instance, we have previously shown that alpha-soluble *N*-ethylmaleimide sensitive factor (NSF) attachment protein (α-SNAP), a well-known synaptic family adaptor protein that forms a part of the 20S SNARE super-complex (Yoon and Munson, 2018), is a crucial component of the CRAC channel supramolecular complex (Vig et al., 2006b; Miao et al., 2013). α-SNAP independently binds Stim as well as Orai and is required for the correct assembly and calcium selectivity of CRAC channels (Li et al., 2016; Miao et al., 2017; Ramanagoudr-Bhojappa et al., 2021). α-SNAP depletion revealed that Stim:Orai coupling is necessary but not sufficient for SOCE, a process that requires distinct time-resolved molecular steps in vivo.

In addition to α-SNAP, SNAREs also came up as candidates in two genome-wide RNAi screens for regulators of SOCE but were not characterized (Vig et al., 2006b; Zhang et al., 2006). Furthermore, SNAREs have been previously found to form inhibitory associations with specific ion channels, but their exact role was not defined (Jarvis and Zamponi, 2005). Therefore, we hypothesized a more fundamental function for the association of SNAREs with ion channel pore subunits and set out to test this in the context of SOCE. Using a targeted RNAi screen, biochemical and biophysical analyses, we have found a direct role of STX11 in facilitating the assembly of CRAC channel pore subunit, Orai1, into optimal multimers. Assembly of Orai1 was independent of, and preceded, trapping and gating by Stim1. Analyses of T cells derived from a symptomatic STX11 deficient, FHLH4 patient showed that the previously reported (D’Orlando et al., 2013), as well as newly identified, defects in degranulation, interleukin-2 (IL-2) gene expression predominantly stem from suppressed SOCE and not due to a general defect in vesicle trafficking or reduced levels of Orai1 in PM. Based on the findings presented here, we demonstrate a novel SNARE-dependent priming step for CRAC channel pore in the molecular sequence of SOCE and suggest preventive pharmacological interventions for FHLH4 patients.

## Results

**Figure 1.**
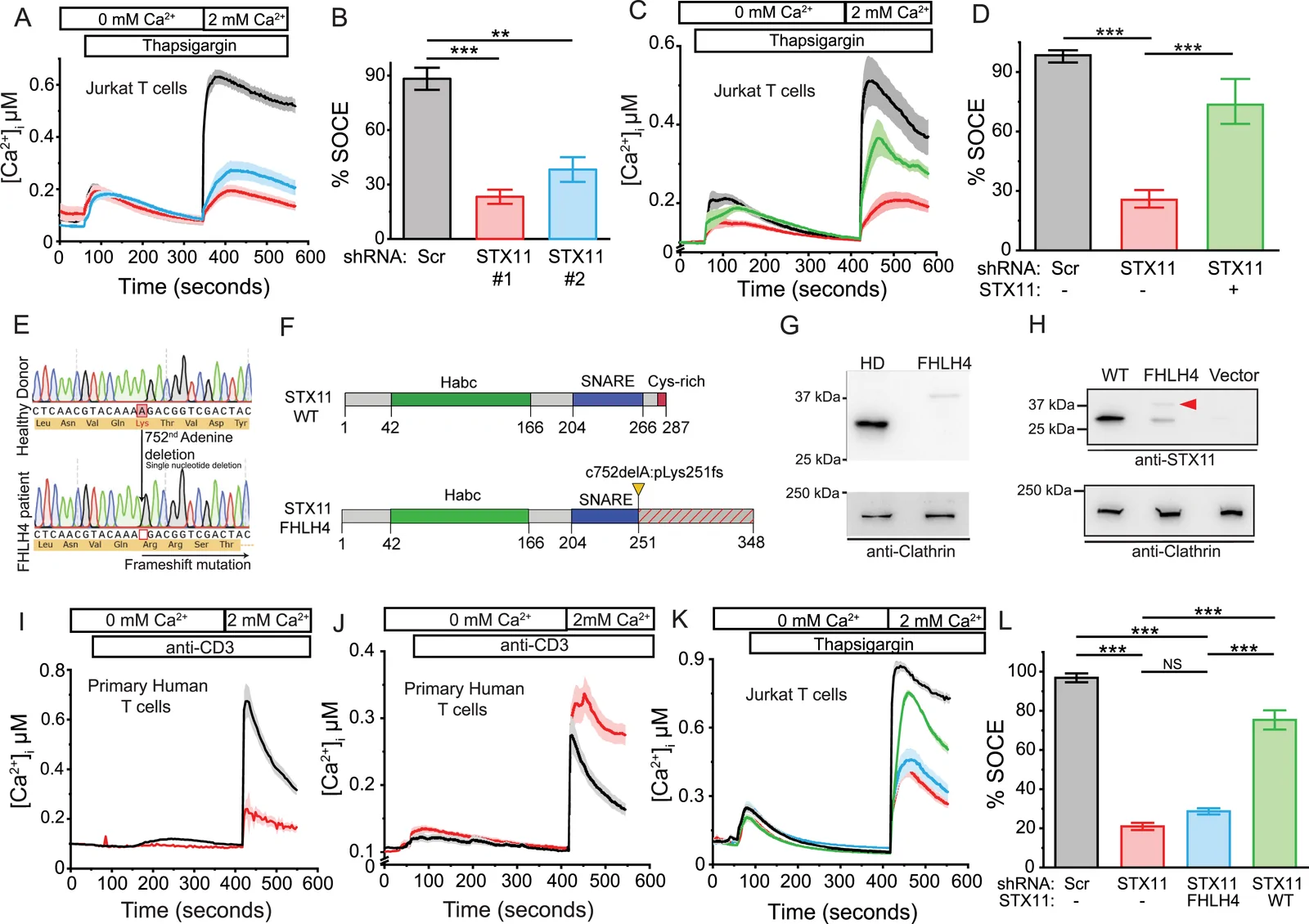
STX11 is required for SOCE. Measurement of SOCE in STX11-depleted Jurkat and FHLH4 patient T cells. (**A–B**) Representative Fura-2 calcium imaging assay (**A**) and quantification of independent repeats (**B**) measuring thapsigargin (TG) induced SOCE in Jurkat T cells treated with Scr (black), STX11#1 (red) or STX11#2 (blue) shRNAs. (n=30–40 cells/group, N=3). (**C–D**) Representative Fura-2 calcium imaging assay (**C**) and quantification of repeats (**D**) showing reconstitution of SOCE in STX11-depleted Jurkat T cells by ectopic expression of STX11. Scr shRNA with empty vector (EV) expression (black), STX11 shRNA with EV expression (red), STX11 shRNA with STX11 expression (green). (n=30–40 cells/group, N=3). (**E**) Sanger sequencing of FHLH4 patient DNA showing deletion of a single adenine at the 752nd position and the resulting frameshift. (**F**) Schematic showing the predicted domain distribution of the wildtype *versus* FHLH4 mutant STX11 protein. (**G**) Western blot on WCLs of healthy donor (HD) and FHLH4 patient PBMCs showing the relative molecular weight and abundance of the wildtype and mutant STX11 bands (red arrow). (**H**) Western blot on WCLs of HEK293 cells over-expressing wildtype or FHLH4 mutant STX11 (red arrow) and empty vector. (**I**) Fura-2 calcium imaging assay measuring anti-CD3 induced SOCE in HD (black) and FHLH4 (red) T cells. (n=30–40 cells/group). (**J**) Fura-2 calcium imaging assay measuring SOCE in HD expressing empty vector (EV) (black) and FHLH4 T cells expressing human STX11 (red), respectively. (n=30–40 cells/group). (**K–L**) Representative Fura-2 calcium imaging assay (**K**) and quantification of repeats (**L**) measuring SOCE in Jurkat T cells treated either with Scr shRNA expressing empty vector (EV) (black), STX11 shRNA expressing EV (red), STX11 shRNA expressing wildtype STX11 (green), or STX11 shRNA expressing FHLH4 mutant STX11 (blue). Figure 1—source data 1.Numerical data underlying the quantitative analyses represented in Figure 1A–D and I–L. Figure 1—source data 2.Folder containing the original sequencing result files, shown in Figure 1E. Figure 1—source data 3.Folder containing labeled PDF files of the original western blots for Figure 1G–H. Figure 1—source data 4.Folder containing the original images of western blots displayed in Figure 1G–H.

RNAi-dependent ablation of a Q-SNARE, Syntaxin 5 (STX5), has earlier shown inhibition of SOCE in a genome-wide screen performed in *Drosophila* S2 cells (Zhang et al., 2006) and STX1A has been previously shown to bind a variety of ion channels (Jarvis and Zamponi, 2005). Therefore, we first knocked down STX5 (Figure 1—figure supplement 1A) and STX1A (Figure 1—figure supplement 1B) using different sequences of shRNA targeting each respective gene in HEK293 cells and measured SOCE in response to Thapsigargin (TG). In both cases, we did not see a significant defect in SOCE. Since several proteins involved in synaptic function are not ubiquitously expressed and many show redundancy in function, we searched online databases to target SNAREs that are expressed in HEK293 and/or T cells (Figure 1—figure supplement 1C–H). We found that STX11 knockdown showed strong and consistent inhibition of SOCE by days 3–4 in HEK293 (Figure 1—figure supplement 2A–B) and Jurkat T cells (Figure 1A–B). Quantitative PCR (qPCR) analysis of total RNA extracted from scramble (Scr) and STX11 shRNA-treated cells showed ~60–70% depletion of STX11 mRNA in both the cell lines (Figure 1—figure supplement 2C–D) and ectopic expression of STX11 in STX11-depleted Jurkat cells rescued SOCE (Figure 1C and D). The efficiency of STX11 depletion was assessed by qPCR for each experiment reported in this study. We restricted our analysis to days 3–4, post RNAi treatment, as longer incubations with RNAi or CRISPR knockout of STX11 affected cell viability.

**Figure 1—figure supplement 1.**
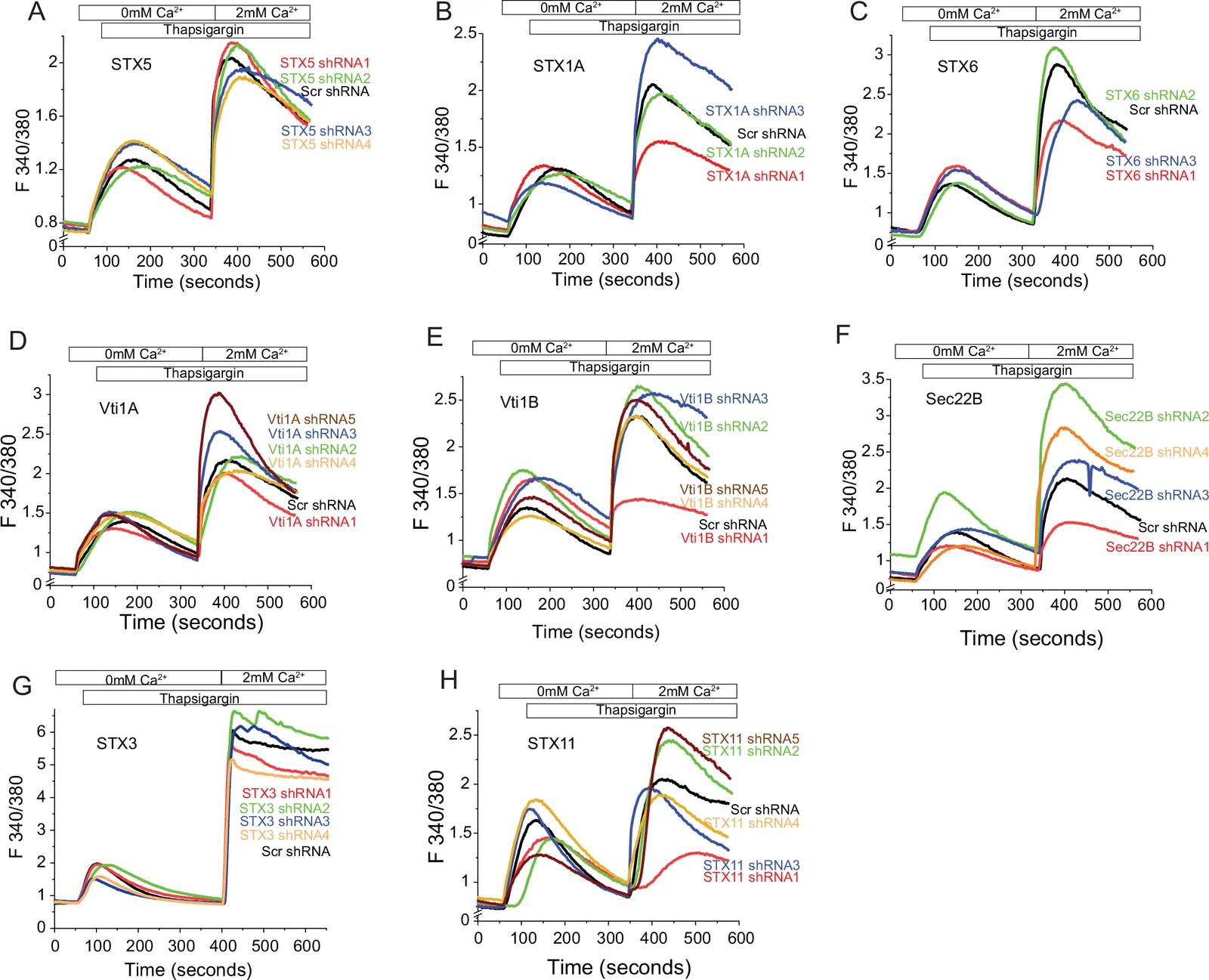
Targeted RNAi screen for genes involved in SOCE. (**A–H**) Fura-2 calcium imaging assays measuring thapsigargin (TG) induced SOCE in HEK293 and/or Jurkat cell lines treated with Scr shRNA or different shRNA sequences against the gene of interest for 3–4 days. Shown here are the average traces from 30 to 40 cells per group. Figure 1—figure supplement 1—source data 1.Numerical data underlying the quantitative analyses represented in Figure 1—figure supplement 1.

**Figure 1—figure supplement 2.**
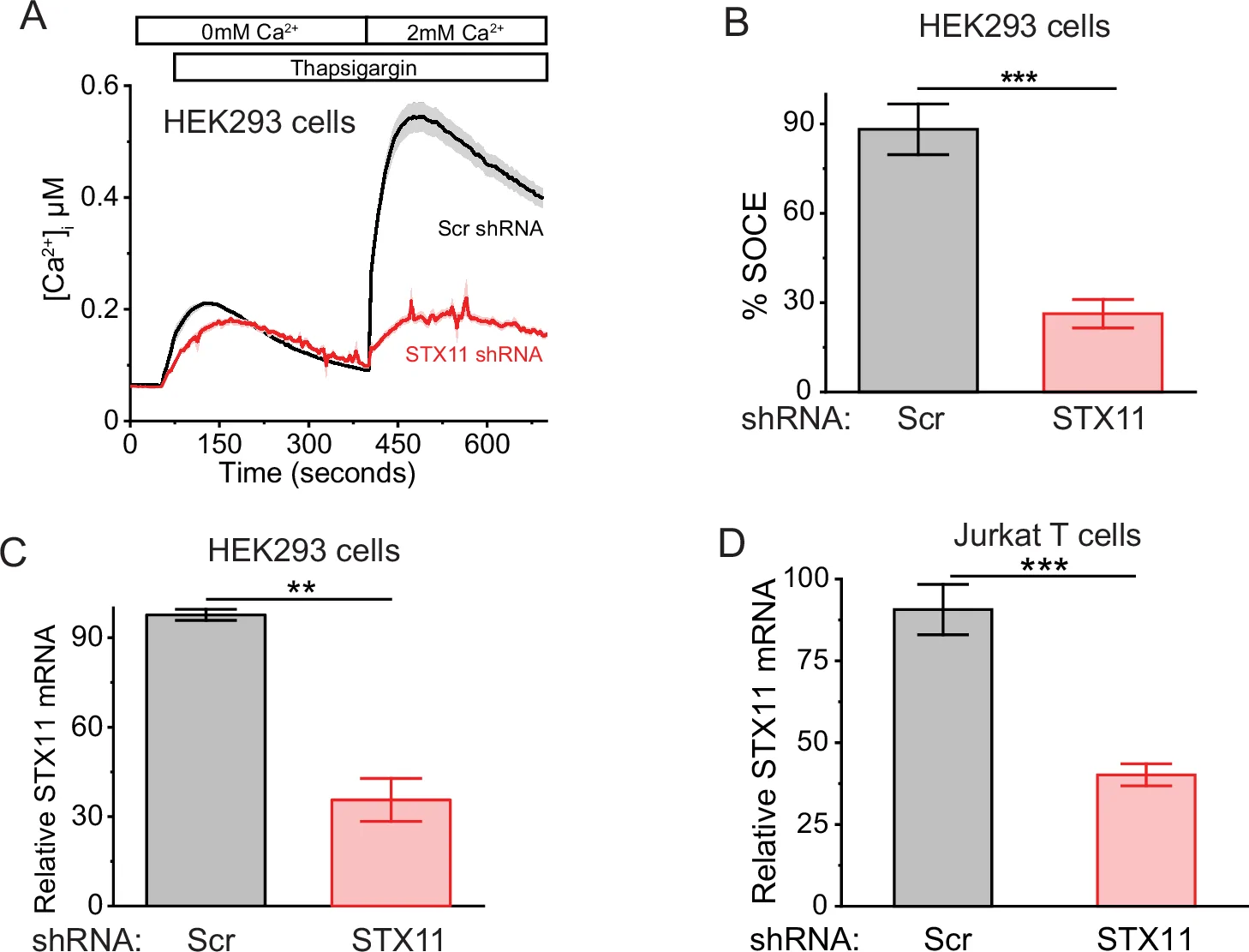
Validation of STX11 knockdown in HEK293 and Jurkat T cells. (**A–B**) Representative Fura-2 calcium imaging assay (**A**) and its quantification from repeats (**B**) measuring thapsigargin (TG) induced SOCE in HEK293 cells treated with Scr or STX11 shRNA. Bars show relative SOCE ± SE from three independent experiments in (**A**) where SOCE from Scr shRNA-treated group in one experiment was set at 100%. (**C**) Quantitative PCR to assess the efficiency of knockdown in shRNA-treated HEK293 (**C**) and Jurkat T cells (**D**) from three independent repeats. Total RNA extracted from cells treated with STX11 shRNA was subjected to qPCR analysis using Taqman probes for STX11. Data were normalized to beta-actin housekeeping control. Figure 1—figure supplement 2—source data 1.Numerical data underlying the quantitative analyses represented in Figure 1—figure supplement 2.

**Figure 1—figure supplement 3.**
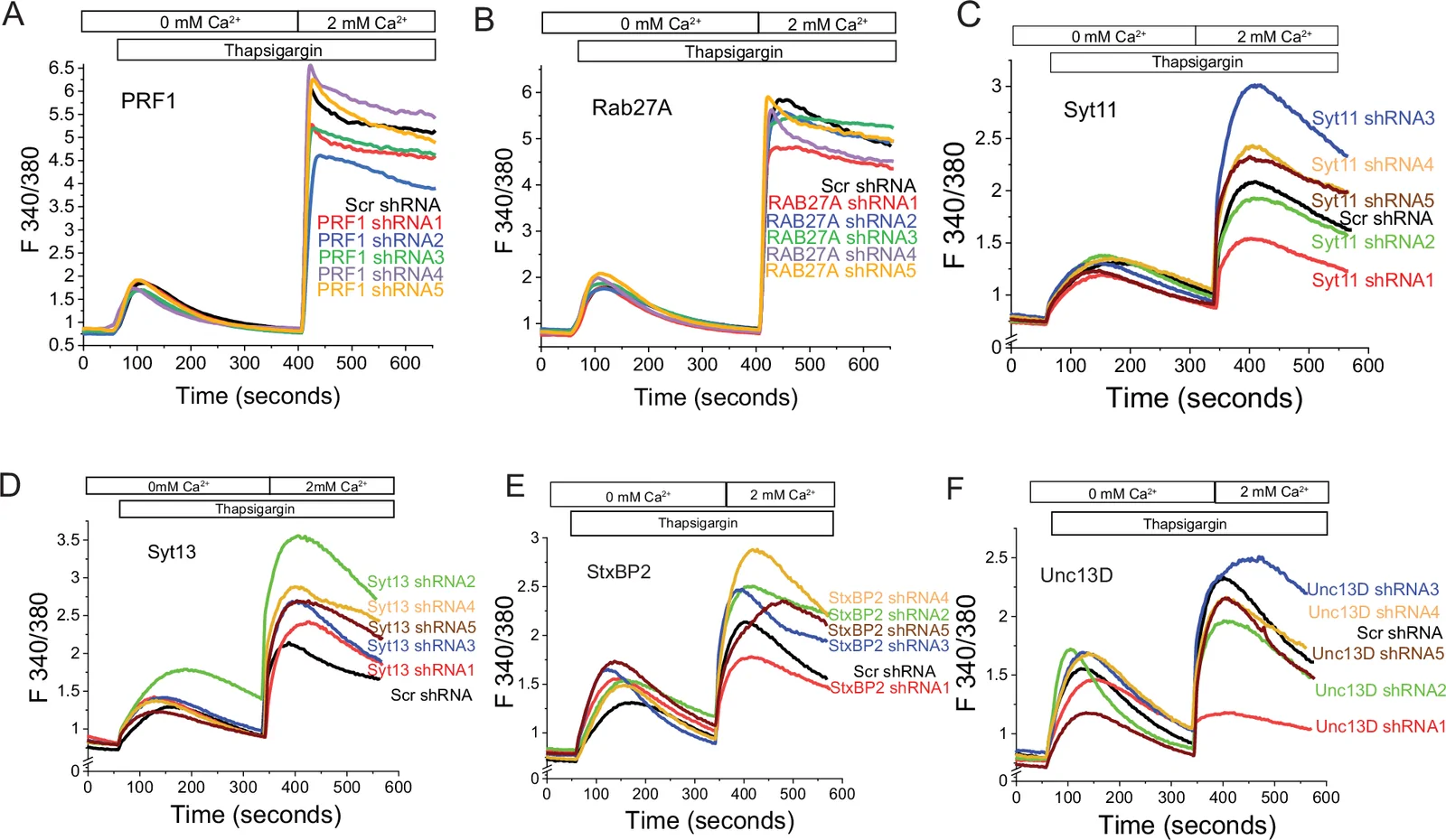
RNAi screen for genes involved in primary HLH and/or vesicle fusion for their role in SOCE. (**A–F**) Fura-2 calcium imaging assays measuring thapsigargin (TG) induced SOCE in HEK293 or Jurkat T cells treated with Scr shRNA or different shRNA sequences against the gene of interest for 3–4 days. Shown here are the average traces from 30 to 40 cells per group. Figure 1—figure supplement 3—source data 1.Numerical data underlying the quantitative analyses represented in Figure 1—figure supplement 3.

Given that STX11 is highly expressed in most immune cells and is the only known SNARE that, when mutated, causes defects in T and NK cell cytotoxicity in humans and mice (D’Orlando et al., 2013), we hypothesized that reduced SOCE contributes to the cytotoxicity defects of FHLH4 patient T cells. To test this hypothesis, we isolated PBMCs from a female FHLH4 patient with a homozygous deletion frameshift mutation in the STX11 coding sequence, c.752delA:p.Lys251fs. The patient presented with all symptoms of FHLH4 disease by 4 years of age. Given the limited sample amount, we expanded PBMC numbers by periodically stimulating patient and healthy donor (HD) cells with phytohemagglutinin (PHA) and IL-2, prior to analysis. Isolation and Sanger sequencing of DNA from patient PBMCs showed deletion of a single adenine at position 752 in the STX11 gene which led to frameshift following lysine 251 (Figure 1E). The absence of any contaminating wildtype peaks showed that the mutation was present in all blood cells. Figure 1F shows a schematic of the predicted mutant STX11 protein with an estimated molecular weight of ~39.5 kDa compared to ~33 kDa for wildtype STX11. The frameshift mutation resulted in disruption of the SNARE domain, ablation of the terminal cysteines, and elongation of the resulting transcript. We subjected whole cell lysates (WCLs) prepared from the FHLH4 patient and HD PBMCs to SDS-PAGE and western blot and found that while the wildtype STX11 band was absent, a faint but distinct band running at ~37 KDa was present in the FHLH4 patient sample lysate (Figure 1G). Therefore, the predicted and observed molecular weight of the FHLH4 mutant protein was higher, but its levels appeared significantly reduced compared to STX11 from HD. This is likely due to protein instability and degradation because ectopic expression of the FHLH4 STX11 mutant in HEK293 cells showed at least two clear bands (Figure 1H), although reduced reactivity to anti-STX11 antibody and premature termination of translation are also possible. Interestingly, all prior FHLH4 STX11 patient mutations that have been biochemically characterized, to date, also showed severe depletion of STX11 protein levels in patient T and NK cells (Bryceson et al., 2007; Marsh et al., 2010; Macartney et al., 2011; Müller et al., 2014). Therefore, the frameshift mutation that has been characterized in our study is representative of most other STX11 human mutations, characterized earlier, in terms of the mechanisms underlying the observed phenotypes. In accordance with our findings in the STX11-depleted cell lines (Figure 1A–B), SOCE was found to be significantly defective in FHLH4 T cells when compared to HD (Figure 1I). Further, expression of wildtype (Figure 1J), but not FHLH4 mutant STX11 (Figure 1K–L), reversed defective SOCE in STX11 deficient patient (Figure 1J) and Jurkat T cells (Figure 1K–L), respectively. These data suggest that, in addition to being unstable, FHLH4 mutant STX11 was functionally compromised and no additional unknown mutations resulted in reduced SOCE.

**Figure 2.**
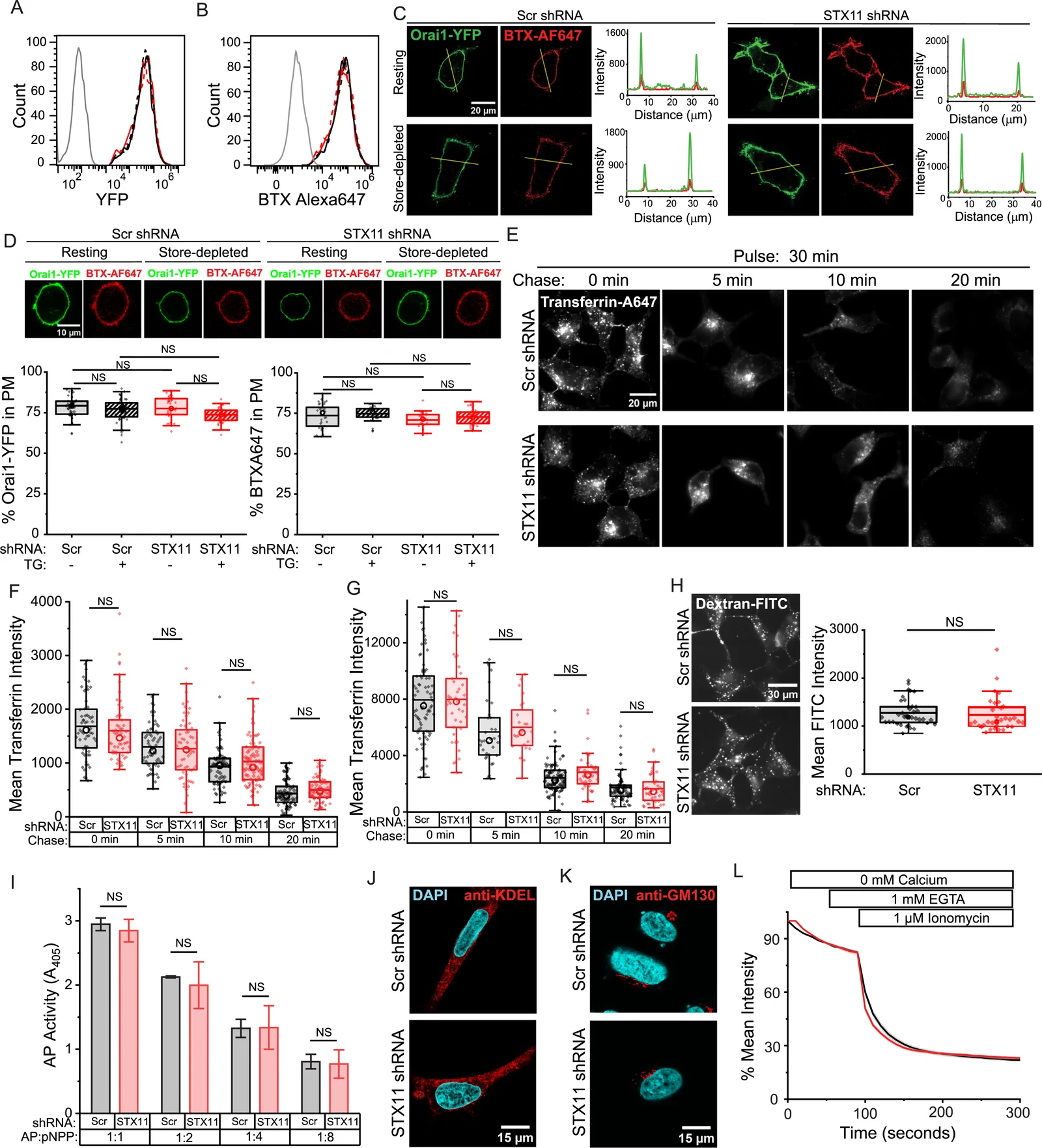
STX11 depletion does not affect PM Orai1 levels and global membrane trafficking or secretion. (**A–C**) Measurement of Bungarotoxin binding site (BBS) tagged Orai1-YFP in the PM of Scr (black) and STX11 shRNA-treated (red), resting (solid line), and store-depleted (broken line), HEK293 cells. Measurement of Orai1-YFP (**A**) and Alexa Fluor 647-conjugated Bungarotoxin binding (**B**) in a population of cells using flow-cytometry. (**C**) Quantification of Orai1-YFP and BTX-A647 in individual cells using confocal imaging. Line profile showing overlay of Orai1-YFP (green) and BTX-A647 (red) signal across the representative cells for individual groups. (**D**) Confocal images and quantification of Orai1-YFP and BTX-A647 in scramble and STX11 shRNA-treated, resting, and store-depleted Jurkat T cells. (n=30–40, N=3) (**E–G**) Quantification of receptor-mediated uptake and recycling in STX11 shRNA-treated cells. Scr or STX11 shRNA-treated HEK293 and Jurkat T cells were loaded with Alexa-647-Transferrin (30 min pulse), washed, chased for 0, 5, 10, and 20 min and imaged to quantify total Transferrin levels inside each cell. Representative images from HEK293 cells (**E**) and quantification from 50 to 60 HEK293 (**F**) and Jurkat T cells (**G**) is shown. N=3 (**H**) Quantification of non-receptor mediated endocytosis in STX11-depleted HEK293 cells. Representative images of FITC-conjugated Dextran uptake in Scr and STX11-shRNA-treated HEK293 cells (left panel) and quantification of the mean intensity per cell (right panel). (**I**) Quantification of biosynthesis and secretion in STX11-depleted cells. Scr or STX11 shRNA-treated HEK293 cells were transfected with secretory alkaline phosphatase (AP) expressing plasmid and levels of AP secreted into the culture supernatants were measured using p-nitrophenyl phosphate (pNPP) substrate at 48 hr post-transfection. (**J–K**) Immunolabeling of HEK293 cells to assess ER (**J**) and Golgi (**K**) health. Scr and STX11 shRNA-treated HEK293 cells were stained with anti-KDEL (**J**) and anti-GM130 antibodies (**K**), respectively, and imaged. (**L**) Measurement of ER calcium content in Scr and STX11 shRNA-treated HEK293 cells using transiently transfected ER-localized CEPIA. Cells were incubated in Ringer’s buffer containing 0 mM calcium followed by 1 mM EGTA, stimulated with 1 μM ionomycin and imaged. (N=3). Figure 2—source data 1.Folder containing the original flow cytometry files and gating strategy for Figure 2A–B. Figure 2—source data 2.Numerical data underlying the quantitative analyses represented in Figure 2C–D, F–I and L.

To determine whether the inhibition of SOCE in Figure 1 resulted from changes in the activity of CRAC channels or their PM expression levels, we quantified the total as well as plasma membrane levels of Orai1 in different cell lines (Figure 2). We generated a stable HEK293 cell line by expressing a previously characterized Orai1 fusion construct containing bungarotoxin binding site (BBS) in the extracellular loop and YFP in the C-terminus (Orai-BBS-YFP; Figure 2—figure supplement 1A; Miao et al., 2013). Resting and store-depleted Orai-BBS-YFP expressing HEK293 cells were labeled with Alexa-647-conjugated alpha-bungarotoxin (BTX-A647), washed, fixed, and analyzed using flow-cytometry (Figure 2A–B) as well as microscopy (Figure 2C) to estimate total expression levels and PM localization of Orai1. We found no difference in the total (Figure 2A) or surface Orai1 (Figure 2B–C) expression in any group. Verification of knockdown efficiency in these HEK293 cells is shown in Figure 2—figure supplement 1B-C. Similar observations were made in Jurkat T cells transiently transduced with pMSCV-Orai1-BBS-YFP (Figure 2D; Figure 2—figure supplement 1D) as well as U2OS cells (Figure 2—figure supplement 2A–E) stably expressing the same plasmid construct as described above for HEK293. Neither total Orai1 expression levels, nor its PM localization was found to be altered upon store-depletion or STX11 ablation in these cell types.

**Figure 2—figure supplement 1.**
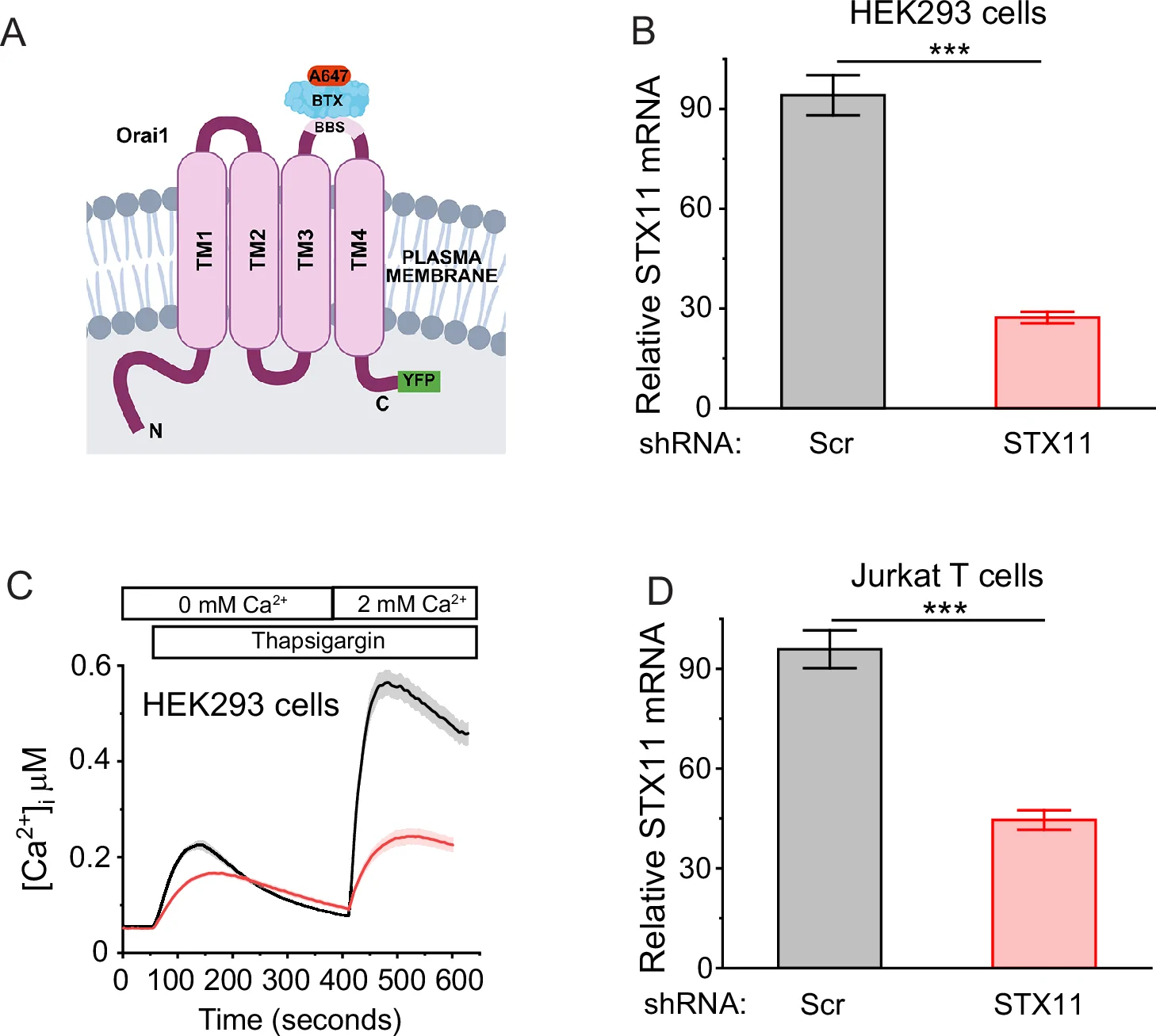
Validation of STX11 knockdown in Orai1-BBS-YFP expressing HEK293 and Jurkat T cells. (**A**) Schematic showing the design of recombinant Orai1-BBS-YFP fusion protein. Shown here is the placement of the bungarotoxin binding site (BBS) inside the second extracellular loop and YFP tag in the C-terminus of Orai1 as well as binding of BTX-A647 to BBS. Created with BioRender.com. (**B**) Representative quantitative PCR to assess the efficiency of knockdown in shRNA-treated HEK293 cells stably expressing Orai1-BBS-YFP. Total RNA extracted from cells treated with STX11 shRNA was subjected to qPCR analysis using Taqman probes for STX11. Data were normalized to RPL30 housekeeping control. (**C**) Representative Fura-2 calcium imaging assay measuring Thapsigargin (TG) induced SOCE in Orai1-BBS-YFP expressing HEK293 cells treated with Scr or STX11 shRNA. (**D**) Representative quantitative PCR to assess the efficiency of knockdown in shRNA-treated Jurkat T cells expressing Orai1-BBS-YFP. Total RNA extracted from cells treated with STX11 shRNA was subjected to qPCR analysis using Taqman probes for STX11. Data were normalized to RPL30 housekeeping control. Figure 2—figure supplement 1—source data 1.Numerical data underlying the quantitative analyses represented in Figure 2—figure supplement 1.

**Figure 2—figure supplement 2.**
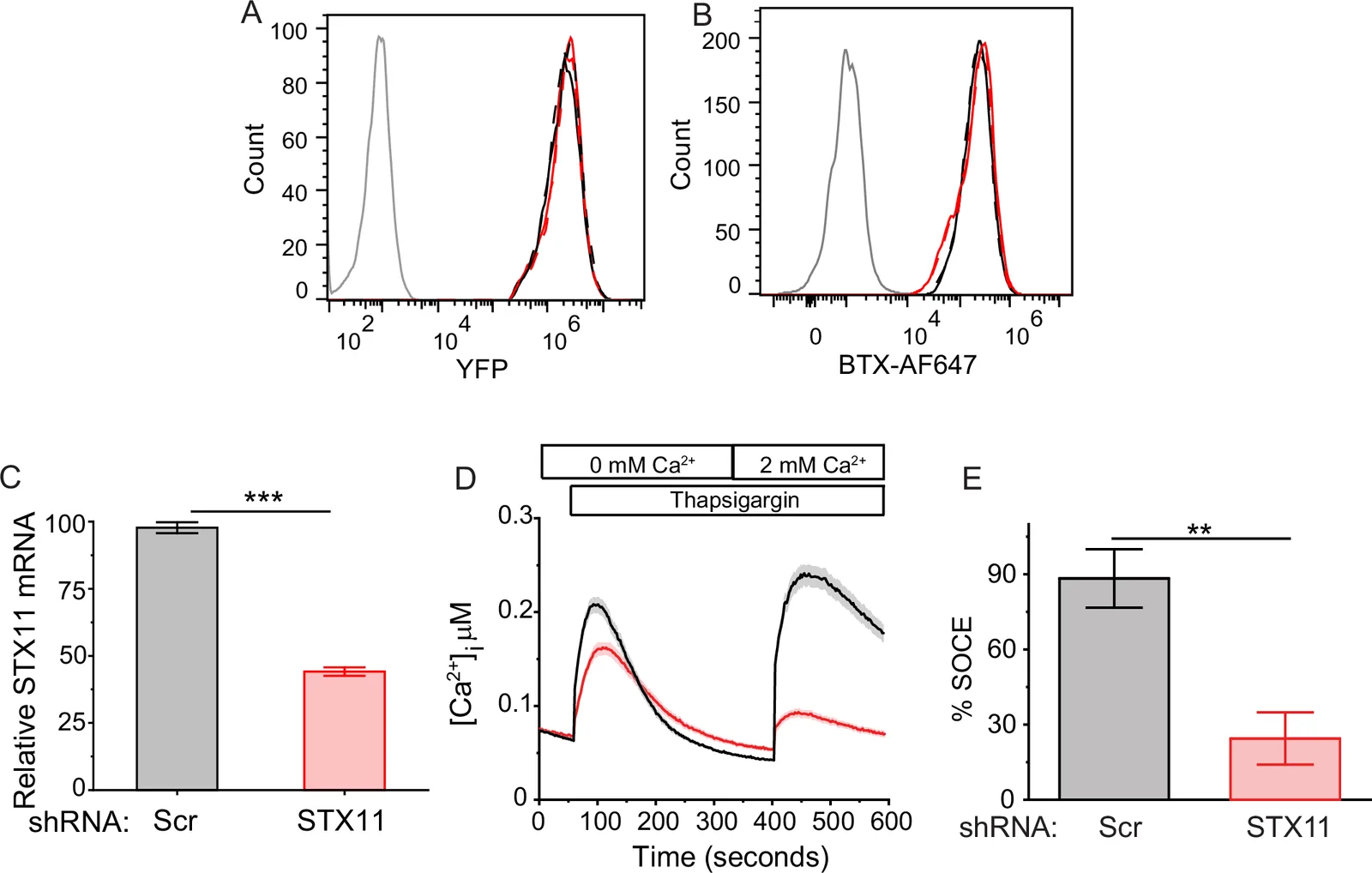
Measurement of total Orai1 levels in the plasma membrane of STX11-depleted U2OS cells. (**A–B**) Measurement of total Orai1 levels in the plasma membrane of STX11-depleted cells by flow-cytometry. U2OS cells stably expressing Orai1-BBS-YFP were transduced with Scr (black) or STX11 (red) shRNA, stimulated with 1 µM TG (broken lines) or control (solid lines) and incubated with alpha-bungarotoxin alexa fluor 647 (BTX-AF647). Total Orai1-YFP levels (**A**) and BTX-AF647 binding to surface Orai1 (**B**) were measured where binding to wildtype U2OS cells was used as the background fluorescence. N=3 (**C**) Representative quantitative PCR to assess the efficiency of knockdown in shRNA-treated Orai1-BBS-YFP expressing U2OS cells from (**A–B**). Total RNA extracted from cells treated with STX11 shRNA was subjected to qPCR analysis using Taqman probes for STX11. Data were normalized to RPL30 housekeeping gene. (**D–E**) Measurement of thapsigargin (TG) induced SOCE in U2OS cells treated with STX11 RNAi in (**A–B**). (**D**) Representative averaged traces of Fura-2 calcium imaging assay performed on cells in panels (**A–B**). (**E**) Bar plot showing relative mean % SOCE ± SE from three independent experiments in (**D**). Figure 2—figure supplement 2—source data 1.Folder containing the original flow cytometry files and gating strategy for Figure 2—figure supplement 2A–B. Figure 2—figure supplement 2—source data 2.Numerical data underlying the quantitative analyses represented in Figure 2—figure supplement 2C–E.

**Figure 2—figure supplement 3.**
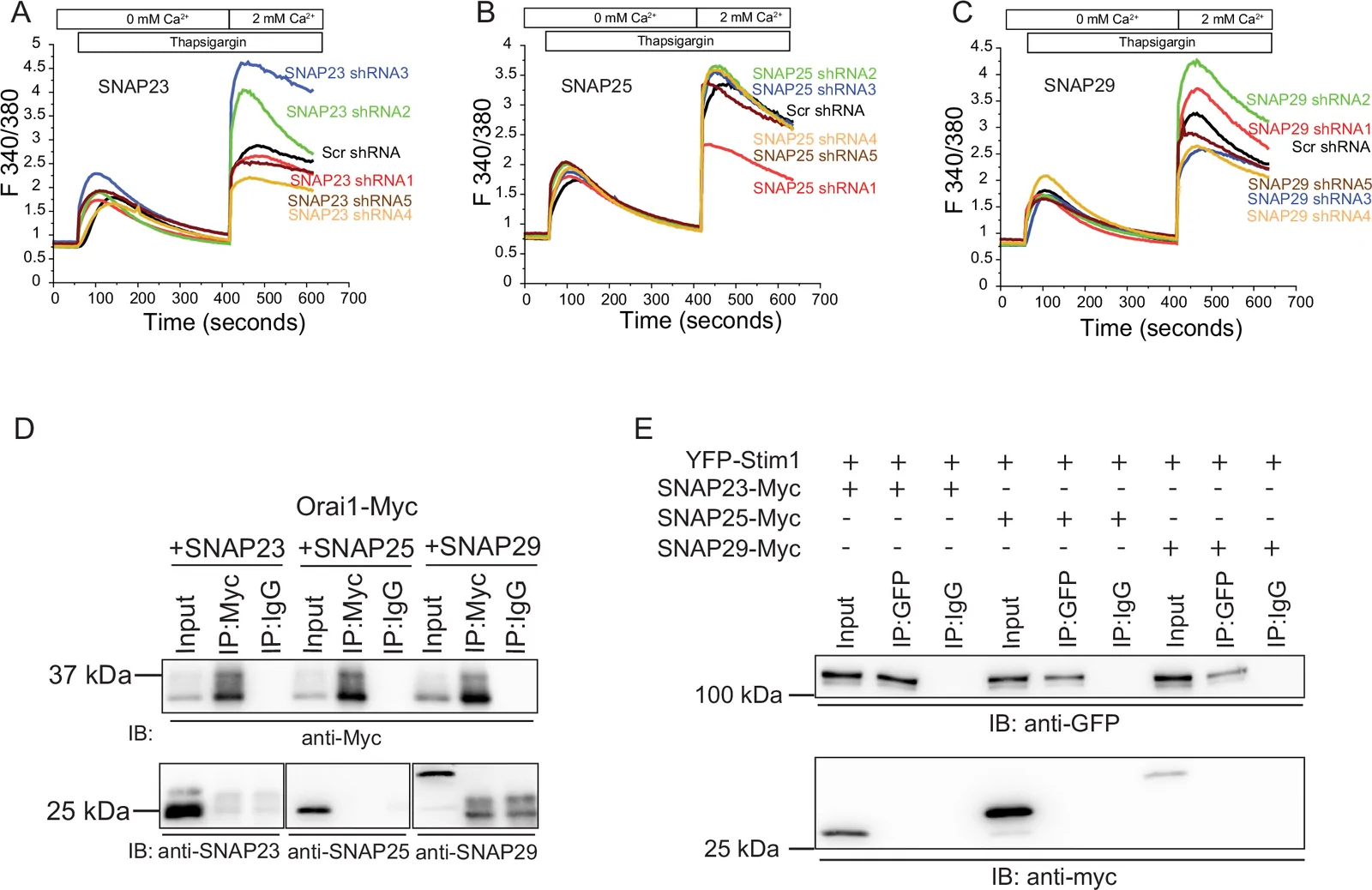
SOCE in RNAi-treated Jurkat T cells to assess the role of SNAPs. (**A–C**) Fura-2 calcium imaging assay measuring SOCE in cells treated with Scr shRNA or different shRNA sequences against SNAP23 (**A**) SNAP25 (**B**) SNAP29 (**C**). Average traces from 30 to 40 cells per group are shown. (**D**) Western blot images of co-IPs to test the association of Orai1 with SNAP23/SNAP25/SNAP29. HEK293 cells were co-transfected with Orai1-Myc and untagged SNAP23/SNAP25/SNAP29, store-depleted, lysed, and subjected to co-IP followed by western blot as indicated. (N=2) (**E**) Western blot images of co-IP to test the association of Stim1 with SNAP23/SNAP25/SNAP29. HEK293 cells were co-transfected with YFP-Stim1 and Myc-tagged SNAP23/SNAP25/SNAP29, store-depleted, lysed, and subjected to co-IP followed by western blot as indicated. (N=2). Figure 2—figure supplement 3—source data 1.Numerical data underlying the quantitative analyses represented in Figure 2—figure supplement 3A–C. Figure 2—figure supplement 3—source data 2.Folder containing labeled PDF files of the original western blots for Figure 2—figure supplement 3D–E. Figure 2—figure supplement 3—source data 3.Folder containing the original images of western blots shown in Figure 2—figure supplement 3D–E.

To determine whether STX11-deficient cells harbored a general defect in vesicle trafficking and fusion, we used the Transferrin receptor (TfR) endocytosis and recycling assay, which internalizes iron bound holo-transferrin via endocytosis of cell surface TfR, followed by exocytosis and extracellular release of iron free apo-Transferrin (McGraw and Maxfield, 1990). RNAi-treated HEK293 (Figure 2E–F) and Jurkat T cells (Figure 2G) were incubated with Alexa-647 conjugated Transferrin (A647-TfR) for a 30-min endocytic pulse. Excess A647-Transferrin was washed off and the emptying of internalized A647-Transferrin via recycling of TfRs over time was quantified at various time points (chase). Levels of A647-Transferrin after endocytosis (30 min pulse, 0 min chase) as well as its exocytosis during 5-, 10-, and 20-min chase times were found to be identical between STX11 or Scr shRNA-treated cells. Non-receptor mediated fluid-phase endocytosis of FITC-conjugated Dextran was also found to be identical between Scr and STX11 shRNA-treated HEK293 cells (Figure 2H). In addition, we transfected HEK293 cells with a plasmid containing alkaline phosphatase (AP) enzyme fused to Ig-κ chain secretion signal peptide at N-terminus, which allowed us to measure AP secreted into the culture supernatant via the biosynthetic-exocytosis pathway. Scr and STX11-depleted cells showed comparable levels of AP secretion (Figure 2I). Overall, these experiments did not indicate any generalized defects in the membrane recycling or secretory pathways upon depletion of STX11 in multiple cell types.

Using a dominant-negative mutant of N-ethylmaleimide-sensitive factor (NSF), which blocked TfR recycling but not SOCE, we have previously shown that CRAC channels are not dependent on vesicle trafficking for their activation (Miao et al., 2013). Yet, Q-SNAREs are often found in complex with other SNAREs such as SNAP23/25 in target membranes and some previous studies have implicated the complex of SNAP23/25 and STX1A in the regulation of specific ion channels (Cormet-Boyaka et al., 2002; Toft-Bertelsen et al., 2016). Each of the three SNAPs, SNAP23/SNAP25/SNAP29 are capable of contributing two SNARE domains to the *trans-*SNARE complex. RNAi-mediated depletion of SNAP23/SNAP25/SNAP29 did not show a significant reduction in SOCE of HEK 293 cells (Figure 2—figure supplement 3A-C). To determine whether SNAP23/SNAP25/SNAP29 might still form a part of the STX11:Orai1 complex to regulate SOCE, we co-expressed myc-tagged Orai1 with untagged SNAPs and performed respective co-IPs. We did not find any interaction between either of the three SNAPs and Orai1 (Figure 2—figure supplement 3D). Similar studies were performed by co-expressing YFP-Stim1 and the three myc-tagged SNAPs. Again, no interaction was found between any of the SNAPs and Stim1 (Figure 2—figure supplement 3E). Collectively, these data established that Orai1 is constitutively expressed in the PM, and not vesicles, of diverse cell types, and STX11 depletion does not lead to a general defect in vesicle fusion or PM targeting of Orai1.

Labeling of STX11-depleted cells with ER, Golgi organelle-specific markers also showed no abnormalities (Figure 2J–K), suggesting that the overall health and function of ER and Golgi were not adversely affected by STX11 depletion. To assess ER calcium content, we ectopically expressed ER-targeted CEPIA in control and STX11-depleted cells and measured the mean intensity. Both groups showed comparable ER calcium content (Figure 2L).

**Figure 3.**
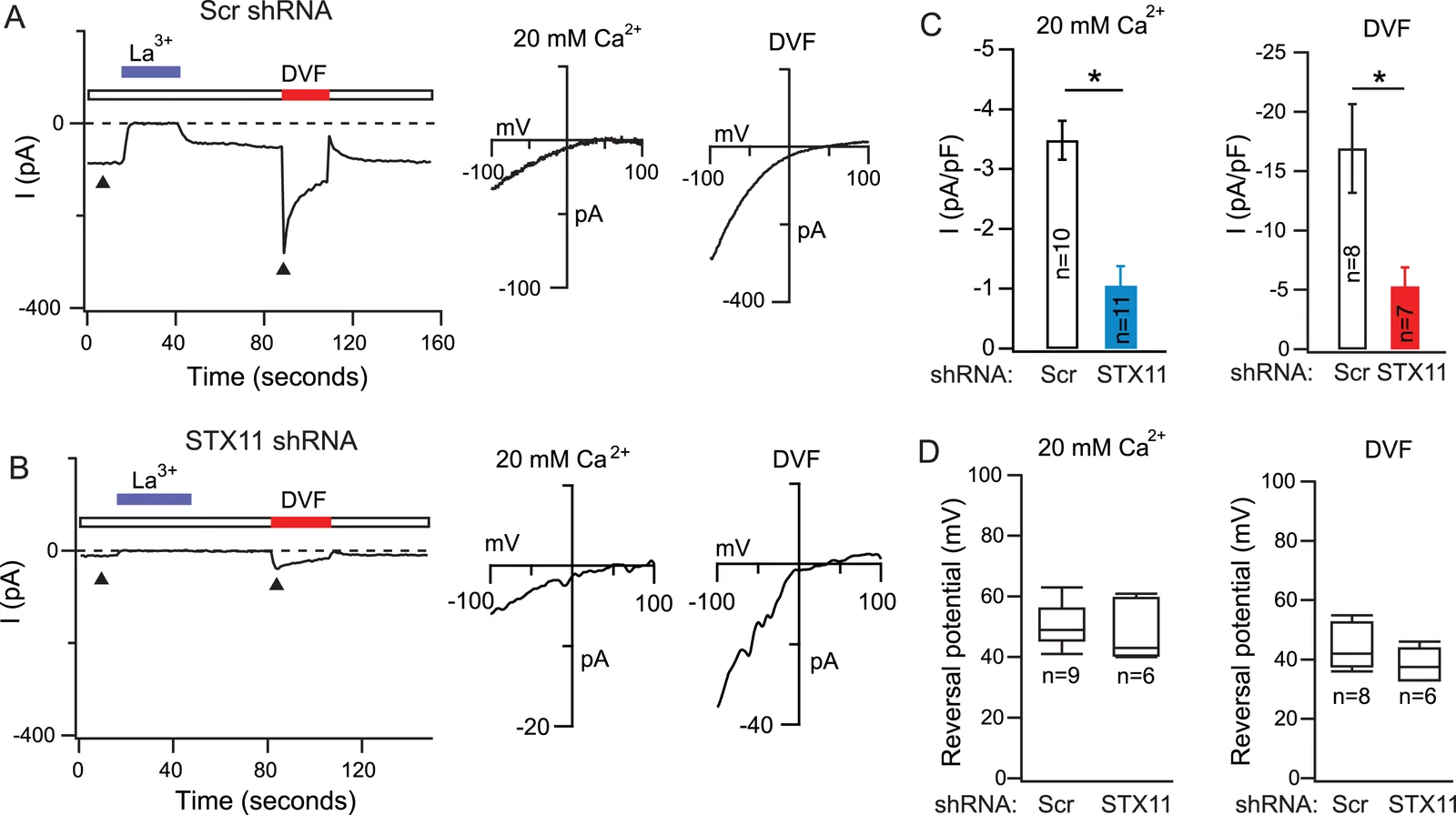
STX11 regulates I_CRAC_. (**A–D**) Measurement of I_CRAC_ in STX11-depleted Jurkat T cells in the whole-cell recording configuration in 20 mM extracellular Ca^2+^ Ringer’s solution. I_CRAC_ was induced by passive depletion of intracellular Ca^2+^ stores by dialyzing 8 mM BAPTA into the cell via the patch-pipette. (**A–B**) Representative CRAC current (I_CRAC_) at –100 mV in Jurkat T cells treated with Scr shRNA (**A**) and STX11 shRNA (**B**) construct. The current is blocked by extracellular La^3+^ (10 µM) and replacing the 20 mM Ca^2+^ Ringer’s solution with a divalent-free solution (DVF) evokes a large Na^+^ current which depotentiates over tens of seconds. The current-voltage (I-V) relationship of the Ca^2+^ and DVF currents are shown on the right. Both Ca^2+^ and Na^+^ current amplitudes are reduced in the STX11 shRNA-treated group relative to Scr control cells. The I–V relationships (right plots) show no change in ion selectivity. (**C–D**) Summary of the current amplitudes of Ca^2+^ and Na^+^ currents (**C**) and current reversal potentials (**D**) in Scr and STX11 knockdown cells. Figure 3—source data 1.Numerical data underlying the quantitative analyses represented in Figure 3.

Previous studies have shown that SOCE in T cells is mediated by CRAC channels formed by Orai proteins. To test whether CRAC current (I_CRAC_) was affected by knockdown of STX11 in T cells, we performed whole cell patch clamp recordings on Scr and STX11 shRNA-treated Jurkat T cells (Figure 3A–B). Leak-subtracted I_CRAC_ was recorded in 20 mM Ca^2+^ and a Na^+^-based divalent cation-free (DVF) solution. These recordings showed that both the Ca^2+^ and DVF CRAC currents were significantly decreased in the STX11 shRNA-treated cells, with the normalized currents shown in Figure 3C. The electrophysiological properties of the residual current in STX11 shRNA-treated cells were indistinguishable from I_CRAC_ in Scr shRNA-treated cells in terms of blockade of the Ca^2+^ current by La^3+^ and depotentiation of the DVF current over tens of seconds. Moreover, the reversal potential of the Ca^2+^ and DVF currents was similar between Scr and STX11 shRNA-treated Jurkat T cells, suggesting that STX11 depletion does not affect the calcium selectivity of CRAC channels (Figure 3D). Together, these results demonstrate that STX11 works by specifically modulating I_CRAC_ in T cells.

**Figure 4.**
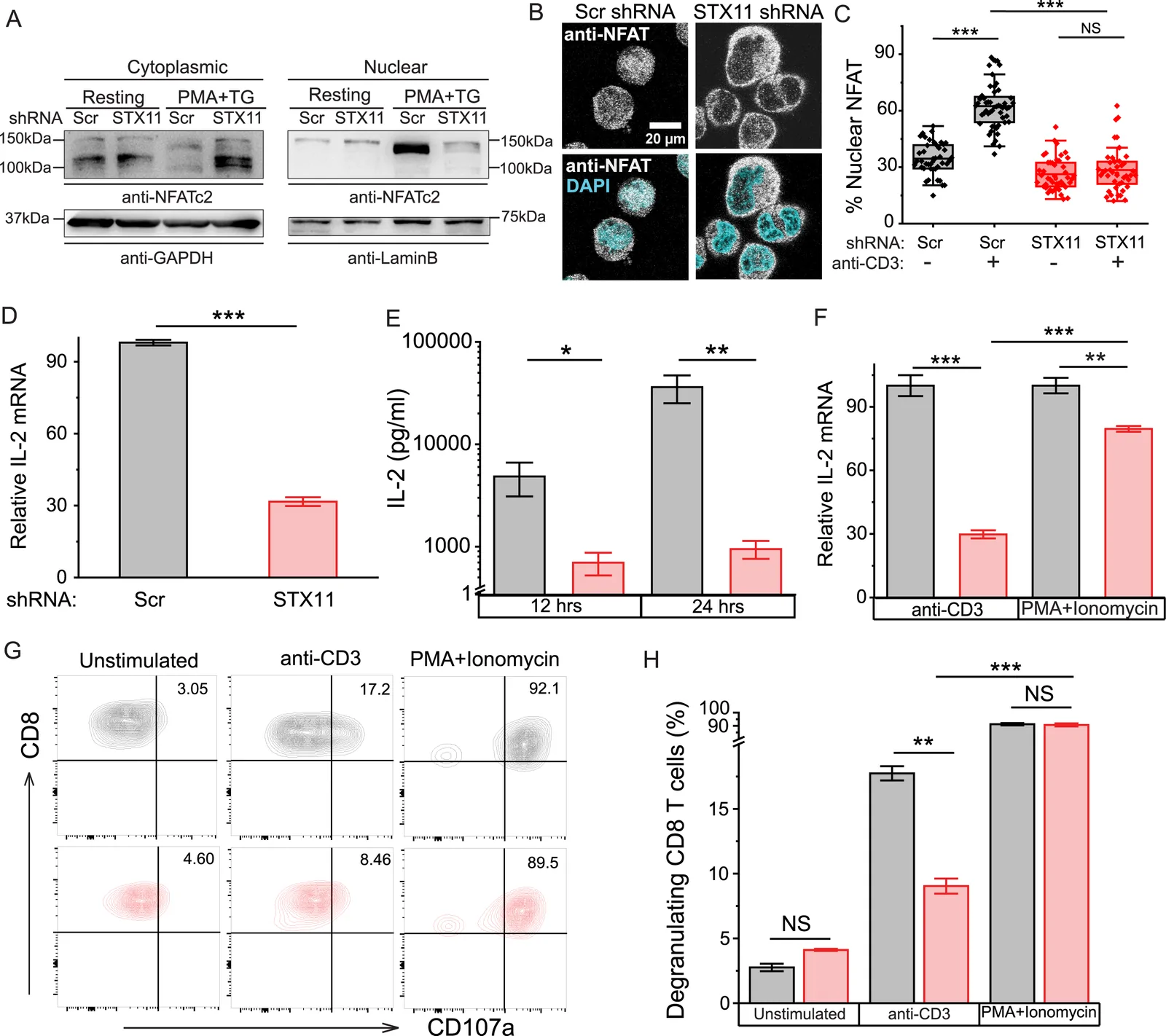
STX11 regulates NFAT activation, IL-2 gene expression, and degranulation in FHLH4 patient T cells. (**A–C**) Estimation of nuclear translocation of NFAT. (**A**) Western blot showing nuclear translocation of NFAT in Jurkat T cells treated with Scr or STX11 shRNA and stimulated with PMA +TG. N=3. (**B**) Representative confocal images of control and STX11-depleted Jurkat T cells stimulated with anti-CD3, immunolabeled with anti-NFAT1 antibody, and counter-stained with DAPI. (N=3) (**C**) Box and whisker plot showing quantification of nuclear NFAT from cells populating 10 randomly chosen fields per group in (**B**). (**D**) Quantification of IL-2 mRNA in anti-CD3 stimulated Jurkat T cells treated with Scr or STX11 shRNA using qPCR. The bars show mean ± SE of relative IL-2 mRNA. N=3. (**E**) Quantification of IL-2 EIA on supernatants of Scr (black) or STX11 (red) shRNA-treated Jurkat T cells stimulated with anti-CD3. (**F**) qPCR analysis of IL-2 mRNA expression in various stimulated HD (black) and FHLH4 T cells (red). (**G–H**) Flow cytometry contour plot showing granule release assay performed on HD (black) and FHLH4 CD8 T (red) cells (**G**) and its quantification (**H**). Figure 4—source data 1.Folder containing labeled PDF files of original western blots for Figure 4A. Figure 4—source data 2.Folder containing the original images of western blots shown in Figure 4A. Figure 4—source data 3.Numerical data underlying the quantitative analyses represented in Figure 4C–F and H. Figure 4—source data 4.Folder containing the original flow cytometry files and gating strategy for Figure 4G.

CRAC channel-mediated sustained calcium influx is crucial for the activation of a key calcium-dependent transcription factor known as nuclear factor of activated T cells (NFAT) (Vig and Kinet, 2007; Vig and Kinet, 2009). We stimulated control and STX11-depleted Jurkat T cells with TG+phorbol myristate acetate (PMA), prepared nuclear, cytosolic extracts, and subjected them to western blot using anti-NFAT1 antibody (Figure 4A). Nuclear translocation of NFAT was severely compromised in STX11-depleted T cells. In addition, immunofluorescence imaging and quantification of total *versus* nuclear NFAT from anti-CD3 stimulated Jurkat cells showed a defect in nuclear translocation of NFAT1 in STX11-depleted cells (Figure 4B–C). To determine whether NFAT-induced gene expression was affected (Vig and Kinet, 2007; Vig and Kinet, 2009), we quantified interleukin-2 (IL-2) mRNA by qPCR and secreted IL-2 levels from culture supernatants of anti-CD3 stimulated Jurkat T cells. STX11-depleted cells showed a significant decrease in IL-2 gene expression as well as secretion (Figure 4D and E). We stimulated HD and FHLH4 patient T cells either through T cell receptor using anti-CD3 or with PMA +Ionomycin and assessed IL-2 gene expression. Importantly, induction of IL-2 expression was significantly defective in FHLH4 patient T cells but could be largely restored with Ionomycin (Figure 4F). We next performed a granule release assay on the in vitro cultured HD and FHLH4 patient CD8 T cells in response to anti-CD3 mediated stimulation and observed a significant defect in the FHLH4 patient CD8 T cell degranulation (Figure 4G and H). To determine whether the reduced granule release results from a direct defect in vesicle fusion or SOCE, we stimulated cells with Ionomycin along with PMA. Remarkably, CD8 T cell degranulation was fully restored in FHLH4 T cells suggesting that defective SOCE primarily causes cytolytic defects in FHLH4 T cells. Of note, the patient exhibited all symptoms of FHLH4 disease, and we observed strong suppression of SOCE (Figure 1I), IL-2 gene expression, and secretion (Figure 4D–F). However, in vitro culture of FHLH4 patient CD8 T cells with IL-2 has been previously shown to partially or fully restore cytolytic defects by other groups (Bryceson et al., 2007; Hackmann et al., 2013). Therefore, considering previous reports, a relatively lower (50%) defect in CD8 T cell degranulation was not surprising (Bryceson et al., 2007; Hackmann et al., 2013). Taken together, the data show that defects in FHLH4 T cell degranulation as well as IL-2 gene expression originate from suppressed SOCE and not vesicle trafficking.

**Figure 5.**
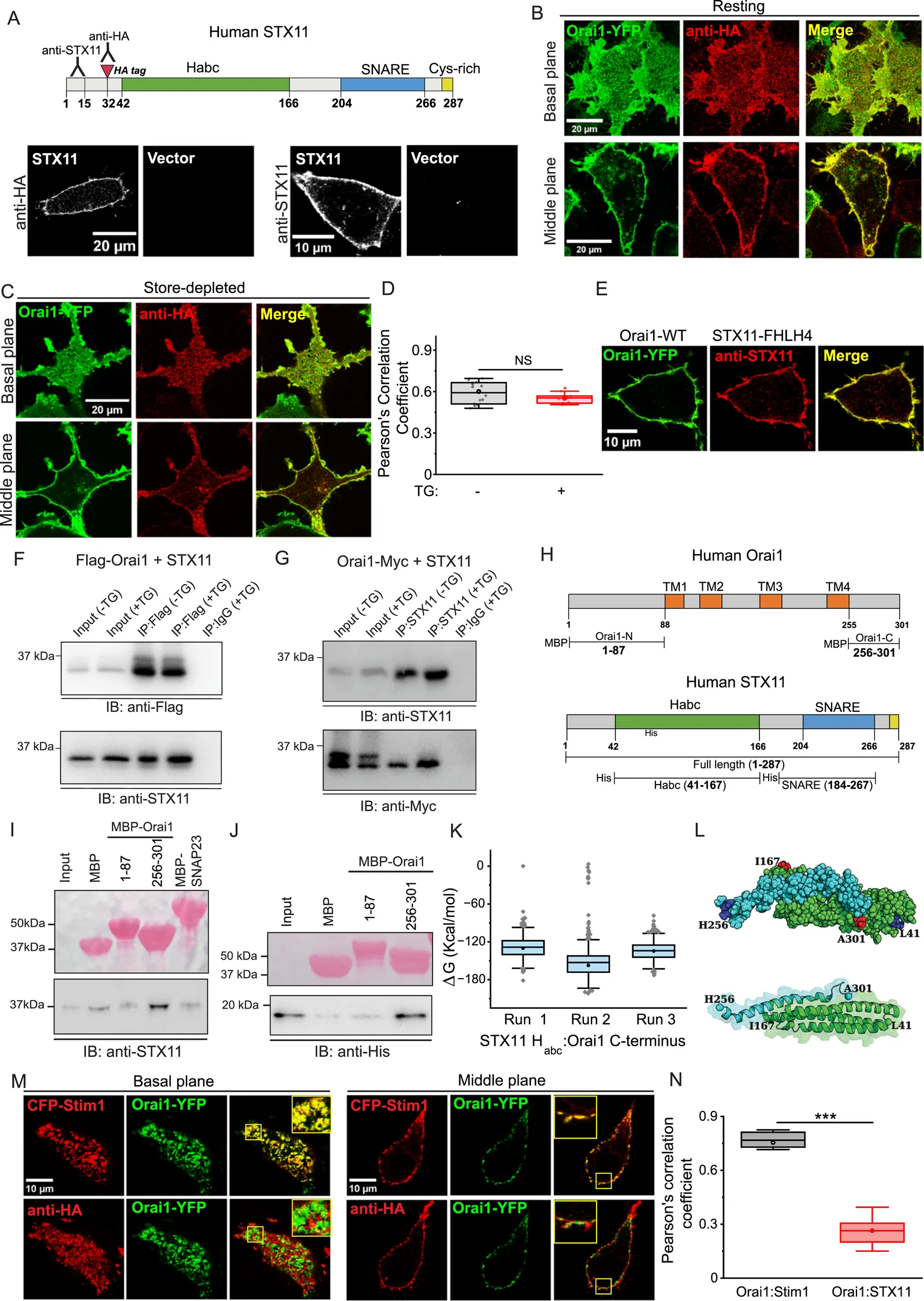
STX11 directly binds resting Orai1 in the plasma membrane. Intracellular localization of STX11 and its association with Orai1. (**A**) (Upper panel) Schematic showing epitopes for anti-STX11 and anti-HA antibodies used in the lower panel. (Lower panel) Representative confocal images of HEK293 cells transfected with either HA-tagged STX11 or untagged STX11 and stained using anti-HA antibody or anti-STX11 antibody, respectively. N=3 (**B–D**) Localization of HA-tagged STX11 with respect to Orai1-YFP in the basal as well as middle plane of resting (**B**) and store-depleted (**C**) HEK293 cells. (**D**) Pearson’s correlation coefficient showing Orai1 and STX11 colocalization from (**B**) and (**C**). N=3. (**E**) Representative confocal images of FHLH4 mutant STX11 expressing HEK293 cells. (**F–G**) Co-IP to assess STX11 binding to Orai1. Whole cell lysates of resting and store-depleted HEK293 cells co-expressing either Flag-Orai1 and STX11 (**F**) or Orai1-Myc and STX11 (**G**) were subjected to IP and western blot using anti-Myc, anti-Flag, or anti-STX11 antibodies, as indicated. (N=3) (**H**) Schematic showing key domains of STX11 and Orai1 used for in vitro pull-down assays. (**I**) Pull-down assay showing in vitro binding of His-tagged STX11 to Orai1. (Top) Ponceau S staining showing the input of MBP alone or MBP-tagged Orai1 fragments. (Bottom) Western blot using anti-STX11 antibody. Input: 1/5^th^ of the protein. N=4. (**J**) Pull-down assay showing in vitro binding of His-tagged H_abc_ domain of STX11 to Orai1 fragments. (Top) Ponceau S staining showing the input of MBP alone or MBP-tagged Orai1 fragments. (Bottom) Western blot using anti-His antibody. Input: 1/5^th^ of the protein. N=3. (**K**) Binding free energy distribution of STX11-H_abc_ and Orai1 C-terminus interactions. (**L**) Sphere and cartoon representation of the structure of STX11 H_abc_ (green) and Orai1 C-terminus (cyan) complex after MD simulation. The N-termini are highlighted in blue, and C-termini in red. (**M**) Representative confocal images showing localization of STX11 with respect to Orai1:Stim1 puncta in store-depleted cells. HEK293 cells expressing Orai1-YFP, CFP-Stim1 were transfected with HA-tagged STX11, store-depleted, and stained using anti-HA antibody. (N=3) (**N**) Pearson’s correlation coefficient showing quantification of the localization of STX11 with respect to Stim1:Orai1 puncta in store-depleted cells from (**M**). Figure 5—source data 1.Numerical data underlying the quantitative analyses represented in Figure 5D, K and N. Figure 5—source data 2.Folder containing labeled PDF files of the original western blots for Figure 5F, G, I and J indicating the relevant bands. Figure 5—source data 3.Folder containing the original images of western blots shown in Figure 5F, G, I and J.

Unlike most Q-SNAREs, STX11 lacks a transmembrane domain but harbors a stretch of cysteines close to the C-terminus and several basic residues, spread throughout the sequence (Figure 5—figure supplement 1A; Lang et al., 2001; Hellewell et al., 2014; van den Bogaart et al., 2011). The cellular localization of STX11 is not established because, unlike primary T cells, native STX11 is undetectable in Jurkat T and HEK293 cell lines using available commercial antibodies. Tagging STX11 on either N- or C-terminus results in partial degradation (Figure 5—figure supplement 1B) and complete mis-localization of the protein (Figure 5—figure supplement 1C). Therefore, we inserted an HA tag inside the N-terminal unstructured region of human STX11. The internal HA tagged STX11 as well as un-tagged STX11 localized in the plasma membrane of most HEK293 cells (Figure 5A) and co-localized with Orai1-YFP in resting (Figure 5B) as well as store-depleted cells (Figure 5C). Figure 5D shows quantification of Orai1 and STX11 co-localization using Pearson’s correlation coefficient. Notably, despite loss of all terminal cysteines and a short terminal part of the SNARE domain, the FHLH4 mutant STX11 did not lose the ability to localize to PM (Figure 5E); therefore, terminal cysteines do not appear to target STX11 to the PM.

**Figure 5—figure supplement 1.**
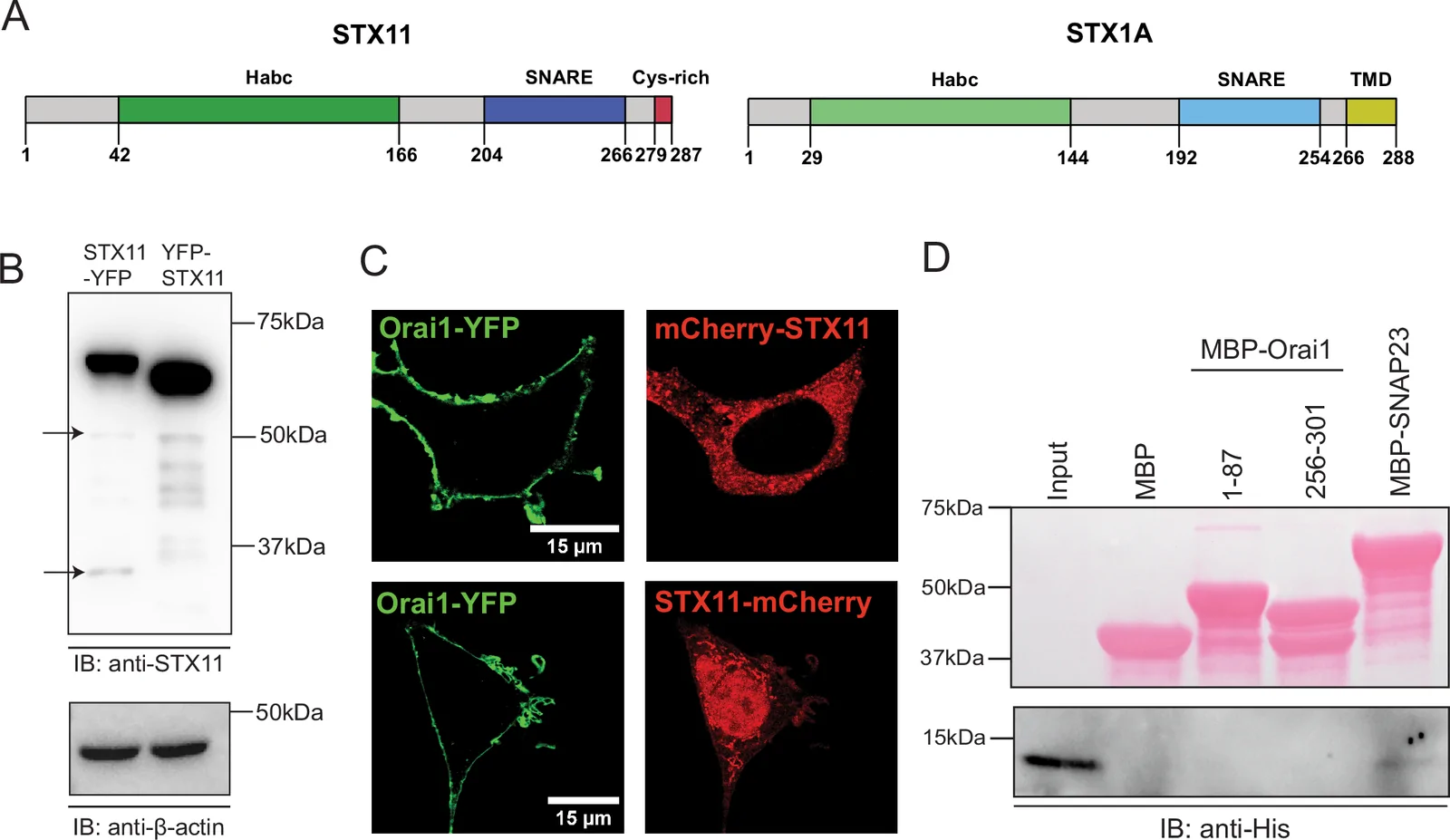
Degradation and mis-localization of N- and C-terminally tagged STX11 and interaction of STX11-SNARE domain with MBP-Orai1. (**A**) Schematic showing a comparison of the key domains in STX11 and STX1A. (**B**) Western blot of whole cell lysates prepared from HEK293 cells expressing YFP-STX11 and STX11-YFP. Note the mismatch in molecular weight of the N- versus C-terminally tagged STX11 and the degradation products (arrows). N=3. (**C**) Confocal images showing localization of ectopically expressed mCherry-STX11 or STX11-mCherry in HEK293 cells. N=3. (**D**) Pull-down assay showing in vitro binding of His-tagged SNARE domain of STX11 to MBP-tagged Orai1 N- and C-termini. (Top panel) Ponceau S staining showing the input of MBP alone or MBP-tagged Orai1 cytosolic tails. (Bottom panel) Western blot using anti-His antibody. Figure 5—figure supplement 1—source data 1.Folder containing labeled PDF files of the original western blots for Figure 5—figure supplement 1B, D. Figure 5—figure supplement 1—source data 2.Folder containing the original images of western blots shown in Figure 5—figure supplement 1B, D.

**Figure 5—figure supplement 2.**
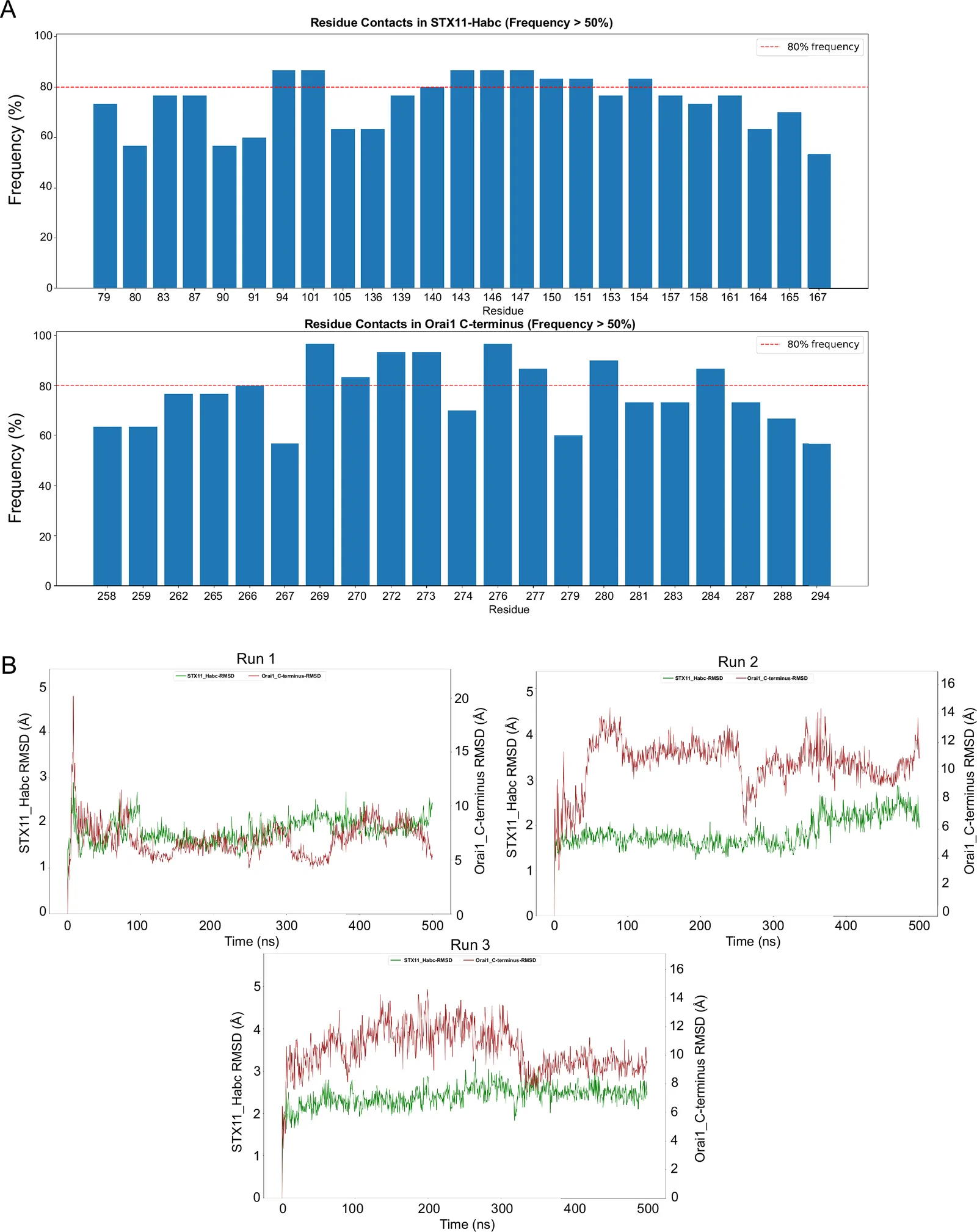
Contact frequency and RMSD of STX11-Habc and Orai1 C-terminus from AF3 predicted models. (**A**) Frequency of interface residues of STX11-Habc and Orai1 C-terminus across AF3 predicted models. Residues with more than 50% frequency are shown. (**B**) RMSD of STX11 Habc and Orai1 C-terminus upon superposition on the 0th ns frame. The STX11-Habc RMSD indicates the backbone RMSD upon superposition of all frames on the STX11-Habc backbone of 0th ns. The Orai1 C-terminus RMSD suggests the stability of Orai1 with respect to STX11 and is calculated for the Orai1 C-terminus after superposition of all frames on the STX11-Habc backbone of 0th ns. Figure 5—figure supplement 2—source data 1.Numerical data underlying the quantitative analyses represented in Figure 5—figure supplement 2. Figure 5—figure supplement 2—source data 2.Folder containing the custom script used in Figure 5—figure supplement 2A.

**Figure 5—figure supplement 3.**
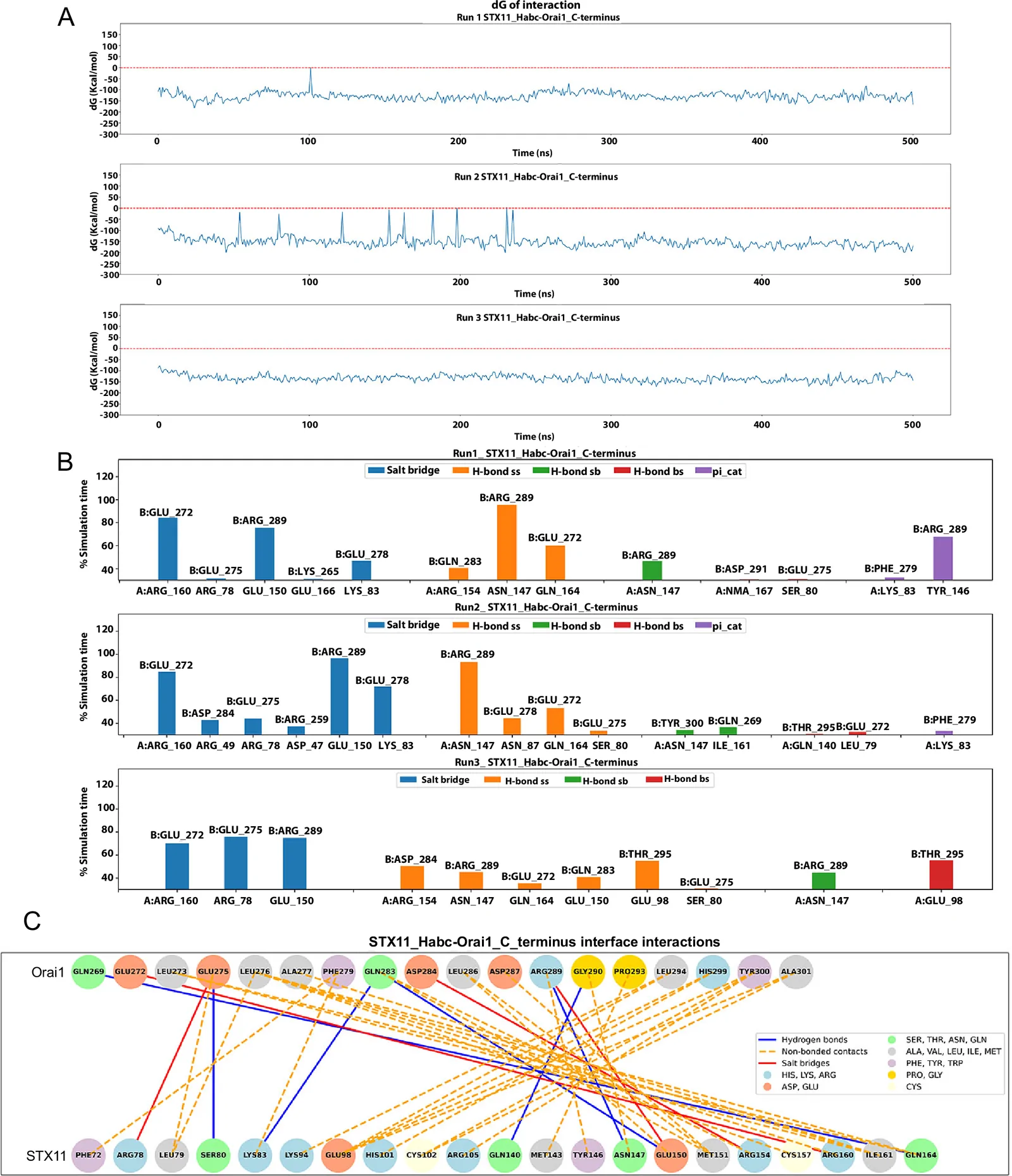
Binding free energy, types of interactions and interacting residues of STX11-Habc and Orai1 C-terminus complex. (**A**) Binding energy (ΔG) of STX11-Habc and Orai1 C-terminus complex across the simulation time for all three replicates. (**B**) Interactions between STX11-Habc and Orai1 C-terminus across the simulation time in three replicates. The interactions that persist for more than 30% of simulation time are shown and highlighted in different colors. H-bond has been categorized as sidechain-sidechain (ss), sidechain-backbone (sb), and backbone-sidechain (bs). (**C**) Residues predicted to be involved in interaction between STX11 Habc and the Orai1 C-terminus identified from all-atom MD simulations. Figure 5—figure supplement 3—source data 1.Numerical data underlying the quantitative analyses represented in Figure 5—figure supplement 3.

**Figure 5—figure supplement 4.**
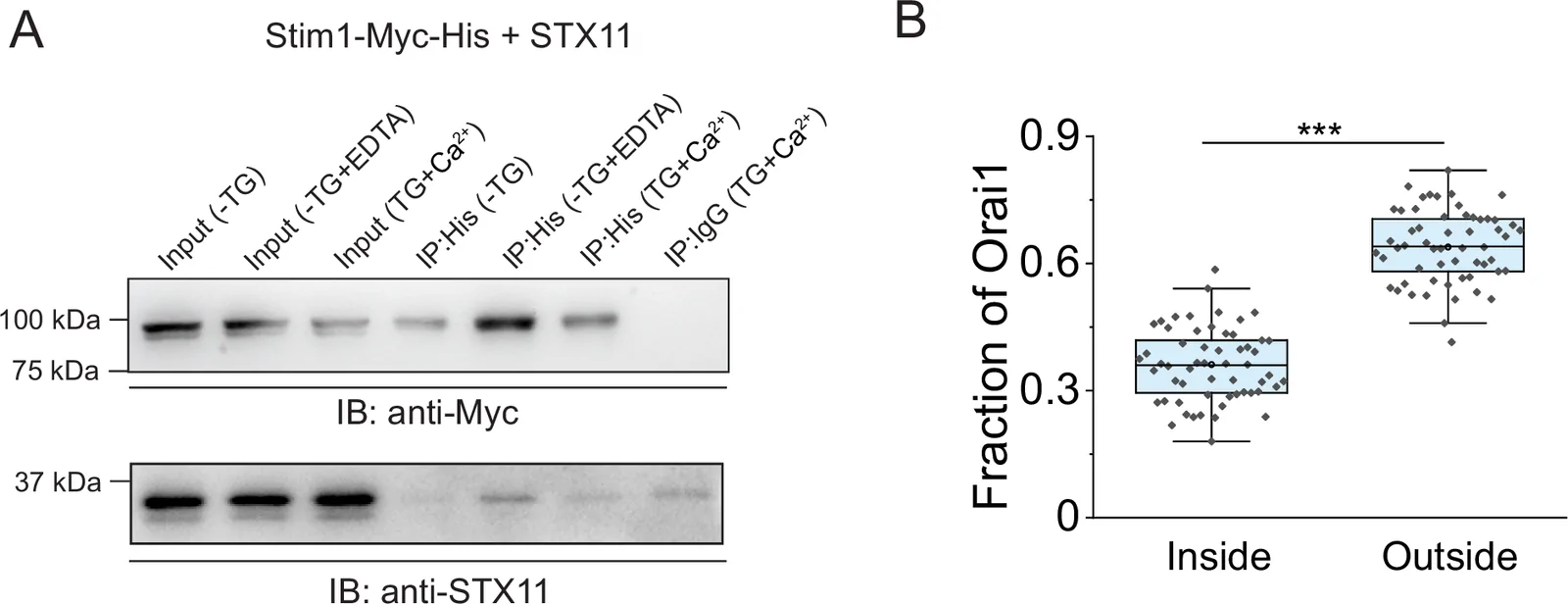
Co-IP of STX11 with Stim1 and measurement of Orai1 inside Stim1:Orai1 clusters. (**A**) Co-immunoprecipitation (co-IP) to test the association of Stim1 with STX11. HEK293 cells were co-transfected with Stim1-Myc-His and STX11, store-depleted, lysed, and subjected to co-IP followed by western blot as indicated. (N=2) (**B**) Quantification of the fraction of Orai1 inside and outside Stim1:Orai1 clusters in store-depleted HEK293 cells expressing CFP-Orai1 and Stim1-YFP. Figure 5—figure supplement 4—source data 1.Folder containing labeled PDF files of the original western blots for Figure 5—figure supplement 4A. Figure 5—figure supplement 4—source data 2.Folder containing the original images of western blots shown in Figure 5—figure supplement 4A. Figure 5—figure supplement 4—source data 3.Numerical data underlying the quantitative analyses represented in Figure 5—figure supplement 4B.

**Figure 5—figure supplement 5.**
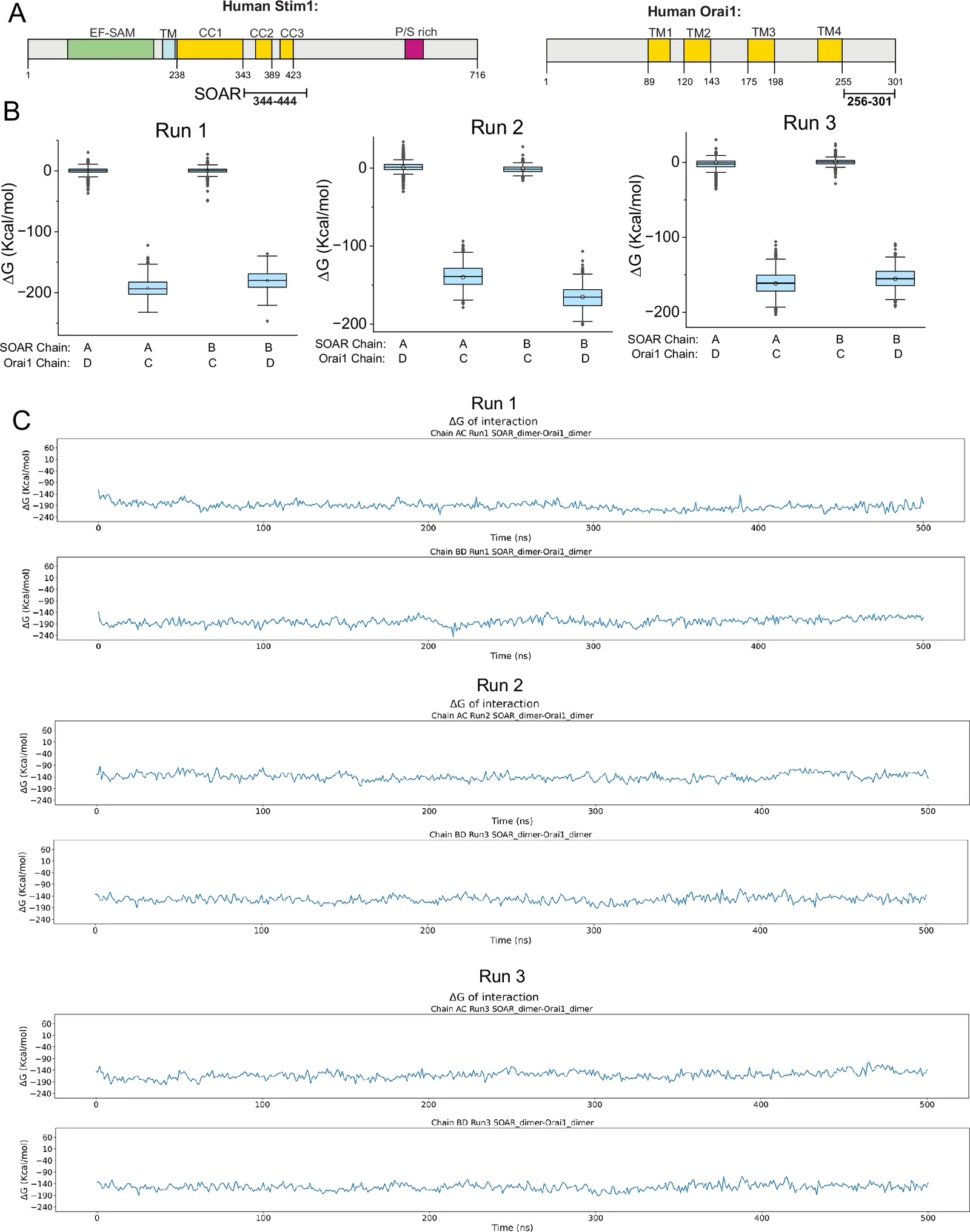
Binding free energy (ΔG) of Orai1 C-termini and SOAR dimer complex. (**A**) Schematic of human Stim1 and Orai1 showing the domains used for MD simulation. TM: Transmembrane, P/S: Proline/Serine rich, and CC: represent Coiled coil domains. (**B**) ΔG values of the three runs across all chains of SOAR dimer and Orai1 C termini. (**C**) ΔG values of chains AC and BD of SOAR dimer and Orai1 C-termini complex across the simulation time for all three runs. Figure 5—figure supplement 5—source data 1.Numerical data underlying the quantitative analyses represented in Figure 5—figure supplement 5B–C.

**Figure 5—figure supplement 6.**
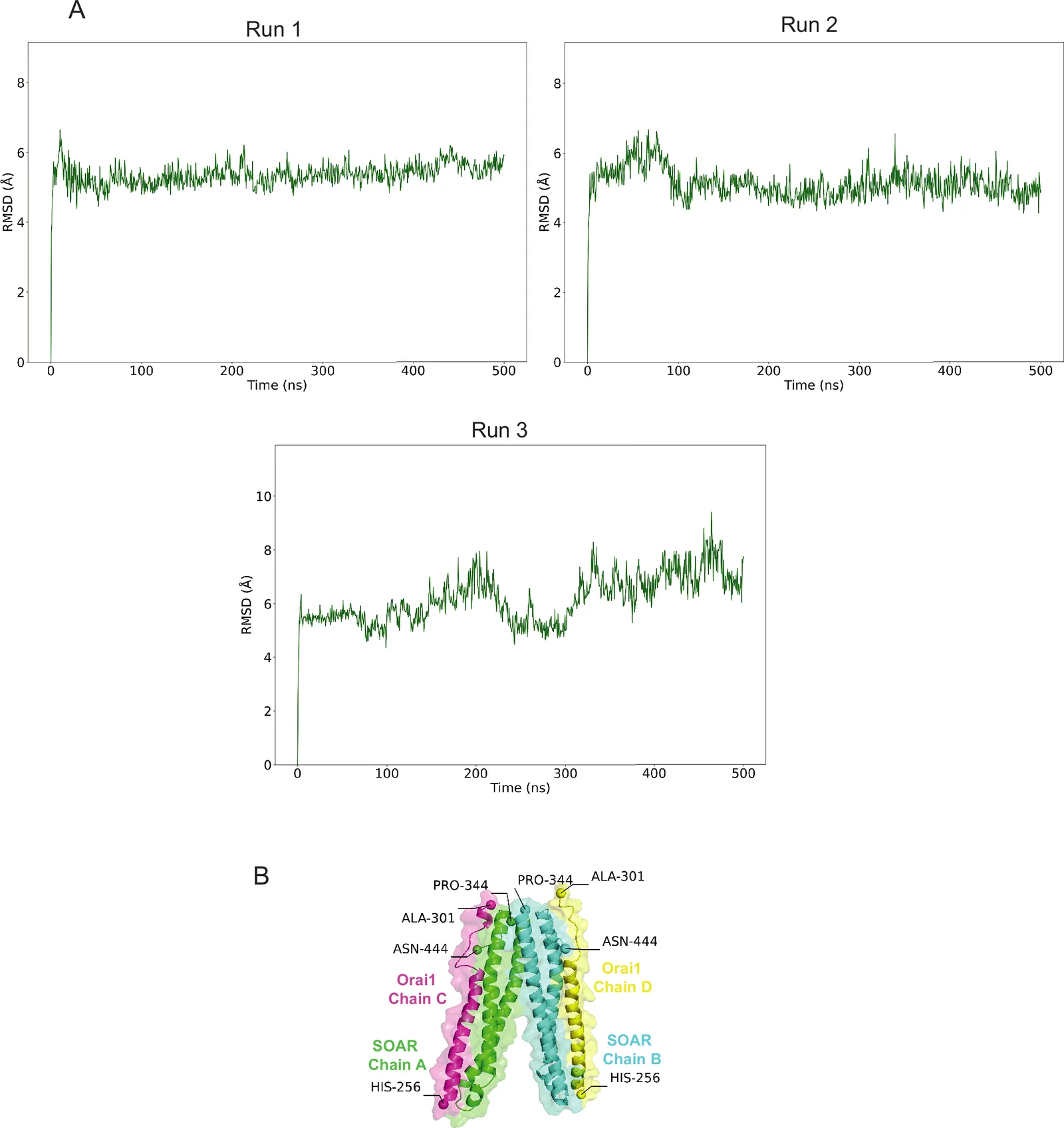
RMSD and cartoon representation of SOAR and Orai1 C-termini complex. (**A**) RMSD of SOAR dimer and Orai1 C-termini complex. (**B**) Cartoon representation of the structure of Stim1-SOAR (344-444) dimer in complex with Orai1 C-termini. Figure 5—figure supplement 6—source data 1.Numerical data underlying the quantitative analyses represented in Figure 5—figure supplement 6A.

**Figure 5—video 1.** Molecular dynamic simulation of interaction of Orai1 C-terminus with the STX11_H_ABC_ domain, run 1.

**Figure 5—video 2.** Molecular dynamic simulation of interaction of Orai1 C-terminus with the STX11_H_ABC_ domain, run 2.

**Figure 5—video 3.** Molecular dynamic simulation of interaction of Orai1 C-terminus with the STX11_H_ABC_ domain, run 3.

**Figure 5—video 4.** Molecular dynamic simulation of interaction of Orai1 C-terminus with the Stim1 SOAR domain, run 1.

**Figure 5—video 5.** Molecular dynamic simulation of interaction of Orai1 C-terminus with the Stim1 SOAR domain, run 2.

**Figure 5—video 6.** Molecular dynamic simulation of interaction of Orai1 C-terminus with the Stim1 SOAR domain, run 3.

To test whether STX11 and Orai1 formed a complex, we co-expressed STX11 with either Flag- or Myc-tagged Orai1 in HEK293, prepared WCLs, and subjected them to co-immunoprecipitation (co-IP) followed by western blot using either anti-Flag, anti-Myc, or anti-STX11 antibodies. Both Orai1 and STX11 could co-IP each other (Figure 5F–G). To further assess whether STX11 directly bound Orai1, we expressed and purified from *E. coli*, MBP-tagged N- and C-terminal cytosolic tails of Orai1 (Figure 5H). In vitro pull-down assay performed by incubating MBP-tagged Orai1 cytoplasmic tails with full-length soluble His-tagged STX11 showed that the C-terminus of Orai1 directly bound STX11 (Figure 5I). SNAREs typically utilize their SNARE domain for associating with other SNAREs (Jahn and Scheller, 2006). To identify the domain of STX11 involved in the regulation of SOCE, we expressed His-tagged H_abc_ and SNARE domains of STX11 (Figure 5H) in *E. coli*, purified, and assessed their binding to the MBP-tagged Orai1 tails using pull-down assay. We found that the H_abc_ domain of STX11 bound to the C-terminus of Orai1 (Figure 5J), but the SNARE domain of STX11 did not show any binding to Orai1 and only a faint binding to SNAP23 (Figure 5—figure supplement 1D). We next used AlphaFold3 (AF3) to obtain deeper insights into the domain interactions of STX11 and Orai1. Of all the possible combinations, the complex of STX11 H_abc_ and Orai1 C-terminus resulted in a significantly high prediction score (Table 1). We, therefore, executed AF3 predictions with different initial seeds to generate multiple models of STX11 H_abc_ with Orai1 C-terminus. We calculated the contact frequency of interface residues in different models and found that multiple residues showed frequency greater than 80% (Figure 5—figure supplement 2A). For further analysis, we considered the predicted model with the best score (Table 2). We next performed all-atom MD simulation in aqueous environment (Figure 5—videos 1–3) to assess the conformation, interaction stability of the STX11 H_abc_ and Orai1 C-terminus complex, which was stable throughout the simulation time as suggested by RMSD (Figure 5—figure supplement 2B), and binding free energy (ΔG) values (Figure 5—figure supplement 3; Figure 5K). The individual trajectories were concatenated and clustered to obtain a centroid structure for this complex. There were 46 clusters, and the largest cluster, with 52 members, showed an elaborate protein-protein interface where STX11 H_abc_ was found to be oriented in an anti-parallel orientation to the Orai1 C-terminus (Figure 5L).

**Table 1.** AF3 predictions for STX11:Orai1 complex formation.

Proteins/domains	ipTM	pTM
Full-length Orai1 - Full-length STX11	0.11	0.35
STX11-Habc_41–167 and Orai1_N-term_1–87	0.27	0.59
**STX11-Habc_41–167 and Orai1_C-term_256–301**	**0.53**	**0.79**
STX11-SNARE_183–267 and Orai1_N-term_1–87	0.08	0.34
STX11-SNARE_183–267 and Orai1_C-term_256–301	0.18	0.45
STX11-Habc_41–167 and full-length Orai1	0.24	0.38
STX11-SNARE_183–267 and full-length Orai1	0.08	0.37
Orai1_N-term_1–87 and full-length STX11	0.15	0.5
Orai1_C-term_256–301 and full-length STX11	0.16	0.54

**Table 2.** AF3 predictions for STX11-H_abc_ and Orai1 C-terminus with different seeds.

Seed values for STX11-H_abc_ and Orai1 C-terminus	ipTM	pTM
111	**0.69**	**0.85**
236	0.51	0.78
174	0.5	0.77
802	0.49	0.76
455	0.56	0.78

Because STX11 binds the C-terminus of Orai1 and the region largely overlaps with the Stim1 binding domain, we next sought to determine whether STX11 clusters with Orai:Stim puncta. We over-expressed HA-tagged STX11 in an Orai1-YFP and CFP-Stim1 expressing stable HEK293 cell line, store-depleted with TG, and stained using anti-HA antibody. We did not observe co-clustering of STX11 in Orai:Stim puncta even 8 min post store-depletion (Figure 5M–N). Accordingly, STX11 did not co-IP with Stim1 in store-depleted cells (Figure 5—figure supplement 4A). These data suggest that STX11 comes off Orai1 C-terminus prior to Stim1-dependent clustering of Orai1. Since co-IP studies suggested sustained interaction between STX11 with Orai1 (Figure 5F–G), we quantified the fraction of Orai1 that resides inside Stim:Orai puncta with respect to total Orai1 in PM and found that post TG addition, nearly 60% of Orai1 remains outside of Stim1 puncta even in unmanipulated cells (Figure 5—figure supplement 4B). We next used AF3 to generate the complex of Stim-Orai activating region (SOAR) domain of Stim1 and Orai1 (Figure 5—figure supplement 5A). Given that SOAR is a dimer under resting state, we used two copies each of SOAR and Orai1 (Li et al., 2011; McNally et al., 2012). The prediction scores for their complex are shown in Table 3. The best scoring model among multiple runs of AF3 was considered for MD simulations on the complex of Orai1 C-terminus (256-301) and SOAR. The ΔG values for the complex are shown in (Figure 5—figure supplement 5B–C). Three independent MD simulations were run for the complex (Figure 5—videos 4–6) which showed an initial conformational change but stabilized thereafter (Figure 5—figure supplement 6A). All three simulation trajectories were concatenated and clustered to obtain a centroid structure for this complex (Figure 5—figure supplement 6B). There were 43 clusters, and the largest cluster had 77 structures. Collectively, these data suggest that STX11 directly binds Orai1 with an affinity that is physiological but relatively poorer when compared to the binding of SOAR dimers to Orai1. Therefore, the presence of a high density of SOAR dimers in ER-PM junctions of store-depleted cells could potentially outcompete STX11:Orai1 interactions by progressively allowing formation of SOAR:Orai1 oligomers. It is also possible that another, yet to be characterized, molecular step and/or protein actively catalyzes the removal of STX11 from Orai1 prior to its entrapment in ER-PM puncta. Future studies will be able to systematically assess these possibilities.

**Table 3.** AF3 predictions for SOAR and Orai1 in a 2:2 ratio.

Two copies of SOAR and 2 copies of Orai1_C-term_256–301	ipTM	pTM
Seed_101	0.73	0.78
Seed_102	0.73	0.78
Seed_103	0.73	0.78
Seed_104	0.73	0.78
**Seed_105**	**0.74**	**0.79**

**Figure 6.**
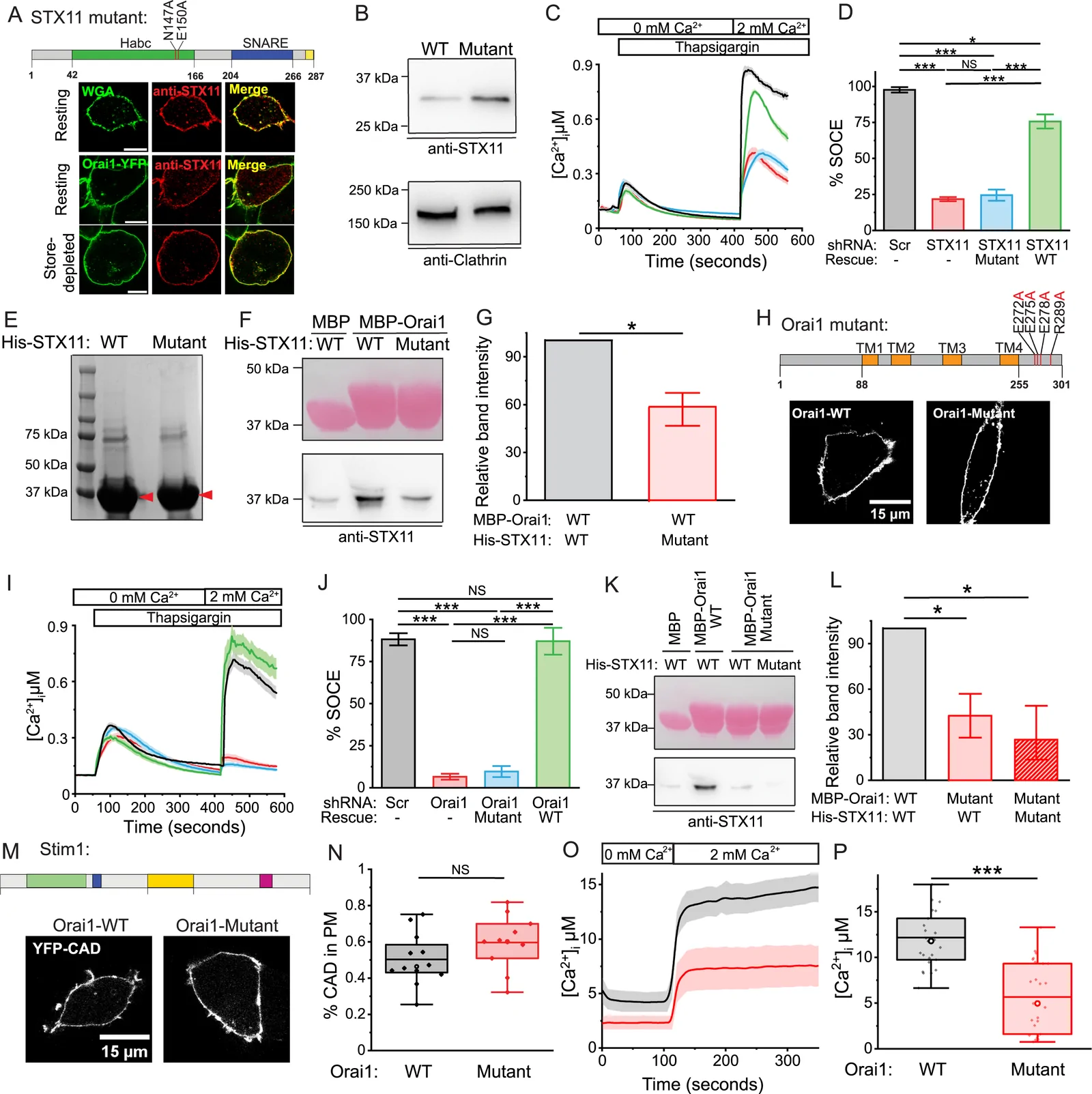
Residues involved in Orai1:STX11 complex formation are crucial for SOCE. Analysis of STX11 and Orai1 mutants. (**A**) Schematic showing STX11 mutations (Top). STX11 N147A_E150A mutant expressing HEK293 co-labeled with Alexa Fluor-488 conjugated wheat germ agglutinin (WGA) or co-transfected with Orai1-YFP and immunolabeled with anti-STX11 to assess co-expression in PM of resting and store-depleted cells (Bottom). Scale bar 10 μm. (**B**) Western blot to compare total expression of mutant and wildtype STX11 using anti-STX11 antibody. (**C–D**) Representative Fura-2 calcium imaging assay (**C**) and quantification of repeats (**D**) measuring rescue of SOCE in Jurkat cells treated either with Scr shRNA (black) or STX11 shRNA expressing empty vector (red), wildtype STX11 (green), or N147A_E150A mutant STX11 (blue). (**E**) Coomassie blue stained SDS-PAGE showing expression and purification of His-tagged wildtype or N147A_E150A mutant STX11 (red arrow) from *E. coli*. (**F–G**) Pull-down assay measuring their binding to MBP-tagged Orai1 C-terminus. (Top) Ponceau S staining showing the input of MBP-tagged Orai1 fragments. (Bottom) Western blot using anti-STX11 antibody. N=4 (**G**) Quantification of STX11 band intensities in (**F**). (**H**) Schematic showing Orai1 mutations (Top). Confocal images of HEK293 cells expressing YFP-tagged wildtype or R289A_E272A_E275A_E278A mutant Orai1 (Bottom). (**I–J**) Representative Fura-2 calcium imaging assay (**I**) and quantification of repeats (**J**) measuring rescue of SOCE in HEK293 cells treated either with Scr (black) or Orai1 shRNA expressing empty vector (red), wildtype Orai1 (green), or R289A_E272A_E275A_E278A mutant Orai1 (blue). (**K–L**) Pull-down assay measuring the binding of MBP-tagged wildtype or R289A_E272A_E275A_E278A mutant Orai1 C-terminus to wildtype or N147A_E150A mutant STX11. (Top) Ponceau S staining showing the input of MBP-tagged Orai1 fragments. (Bottom) Western blot using anti-STX11 antibody. N=4. (**L**) Quantification of STX11 band intensities in (**K**). (**M–N**) Schematic showing CAD domain in Stim1 (top panel) and representative confocal images (lower panel) (**M**) and quantification (**N**) of PM localized YFP-CAD in CFP-tagged wildtype or R289A_E272A_E275A_E278A mutant Orai1 expressing HEK293 cells. (**O–P**) Representative Fura-2 calcium imaging assay (**O**) and quantification of repeats (**P**) of constitutive calcium flux from cells imaged in panels (**M–N**). Figure 6—source data 1.Folder containing labeled PDF files of the original gels and western blots for Figure 6B, E, F, K. Figure 6—source data 2.Folder containing the original images of gels and western blots shown in Figure 6B, E, F and K. Figure 6—source data 3.Numerical data underlying the quantitative analyses represented in Figure 6C–D, G, I–J, L and N–P.

The major interactions predicted between STX11 and Orai1 from all-atom MD simulations are highlighted in (Figure 5—figure supplement 3B–C). To assess the significance of these interactions, we performed site-directed mutagenesis of two key residues of STX11 predicted to be involved in binding to Orai1 (Figure 6A). Ectopic expression of untagged N147A_E150A STX11 showed PM localization (Figure 6A) as well as stable expression (Figure 6B) but failed to rescue SOCE in STX11-depleted Jurkat T cells (Figure 6C–D). We next expressed His-tagged wildtype and N147A_E150A mutant STX11 in *E. coli*, purified (Figure 6E), and assessed their ability to bind MBP-tagged Orai1 C-terminal tail in an in vitro pull-down assay (Figure 6F–G). Compared to wildtype STX11, N147A_E150A mutant STX11 showed reduced binding to Orai1 C-terminus. Further, we introduced four mutations, R289A_E272A_E275A_E278A, in the Orai1 C-terminus (Figure 6H), tagged with YFP and expressed in HEK293 cells. The Orai1 mutant localized to PM (Figure 6H) but failed to rescue SOCE in Orai1-deficient cells (Figure 6I–J). We expressed and purified MBP-tagged R289A_E272A_E275A_E278A mutant Orai1 C-terminus from *E. coli* and assessed its ability to bind purified wildtype as well as N147A_E150A mutant STX11 in a pull-down assay (Figure 6K–L). Binding of Orai1 mutant to both was significantly compromised. Because Stim1 binds an overlapping region of the Orai1 C-terminus, we next tested whether mutation of Orai1 residues affected Stim1 binding. We co-expressed YFP-tagged soluble CRAC activation domain (CAD) of Stim1 (YFP-CAD; Figure 6M) with either CFP-tagged wildtype or mutant Orai1 in HEK293 cell line. YFP-CAD localizes to the cytosol in HEK293 cells but in cells overexpressing Orai1, a significant fraction of it localizes to the plasma membrane due to its association with Orai1 (Prakriya and Lewis, 2015). We quantified relative YFP-CAD levels in the plasma membrane of both groups but found no difference in the PM recruitment of CAD (Figure 6N), suggesting that the inability of R289A_E272A_E275A_E278A mutant Orai1 to support SOCE does not result from defective binding to CAD/SOAR. However, CAD was unable to stimulate constitutive calcium influx from R289A_E272A_E275A_E278A mutant Orai1 in the above experiment (Figure 6O–P). Collectively, these data demonstrate a direct role of STX11 in binding and regulation of Orai1.

**Figure 7.**
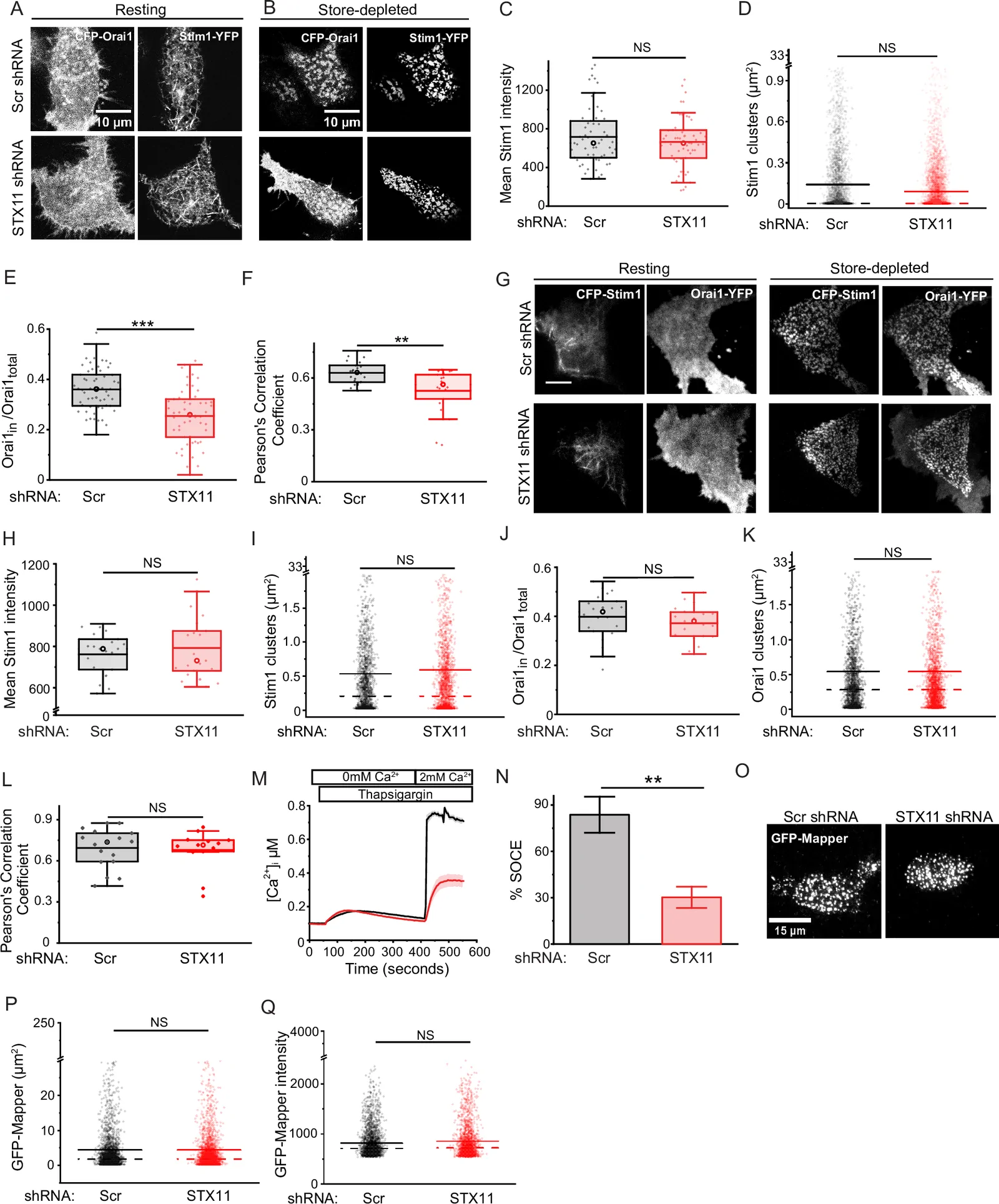
Non-functional clustering of Orai1 in STX11-depleted cells. (**A–L**) Quantification of Orai1 and Stim1 intensities in the ER-PM puncta of store-depleted cells. (**A–B**) Representative images of resting (**A**) and store-depleted (**B**) shRNA-treated HEK293 cells expressing N-terminal CFP-tagged Orai1 (CFP-Orai1) and C-terminal YFP-tagged Stim1 (Stim1-YFP) from confocal microscopy. (**C–F**) Mean Stim1-YFP intensity (**C**) and area (**D**) of Stim1 clusters. (**E**) Fraction of CFP-Orai1 intensities inside Stim1-YFP clusters of shRNA-treated cells, post store-depletion. (**F**) Pearson’s correlation coefficient of Stim1 and Orai1 co-localization inside Stim1 puncta. N=3. (**G–L**) Quantification of Orai1 and Stim1 intensities in the ER-PM puncta of C-terminal YFP-tagged Orai1 (Orai1-YFP) and N-terminal CFP-tagged Stim1 (CFP-Stim1) expressed in HEK293 cells and imaged using TIRF microscopy. (**G**) Representative TIRF images of resting and store-depleted shRNA-treated HEK293 cells. Scale bar 10 μM. (**H–K**) Mean CFP-Stim1 intensity (**H**) and area (**I**) of Stim1 clusters. Fraction of Orai1-YFP intensities (**J**) and area (**K**) of Orai1 clusters in control and STX11-depleted cells. (**L**) Pearson’s correlation coefficient of Stim1 and Orai1 co-localization inside Stim1 puncta. N=2. (**M–N**) Representative Fura-2 calcium imaging assay (**M**) and quantification of repeats (**N**) measuring TG-induced SOCE in Orai1-YFP and CFP-Stim1 expressing HEK293 cells treated with Scr (black) or STX11 (red) shRNA. (**O–Q**) Representative TIRF images of GFP-MAPPER in store-depleted Scr and STX11 shRNA-treated HEK293 cells (**O**). Quantification of area (**P**) and intensity (**Q**) of GFP-MAPPER puncta. n=15. Figure 7—source data 1.Numerical data underlying the quantitative analyses represented in Figure 7C–F, H–N and P–Q.

Upon store depletion, Stim1 localizes to ER-PM junctional regions where it traps and co-clusters with Orai1. To further understand the mechanism, using confocal microscope we first imaged HEK293 cells expressing CFP-Orai1, Stim1-YFP under resting conditions and quantified the levels of Orai1 and Stim1 in ER-PM junctions (puncta) of store-depleted control and STX11-depleted cells. The distribution of Orai1 and Stim1 was normal in resting cells (Figure 7A). However, in store-depleted cells, clustering of CFP-Orai1 was visibly defective in STX11 RNAi-treated cells (Figure 7B), even though the intensities (Figure 7C) and area of Stim1-YFP puncta were normal (Figure 7D). Quantification of Orai1 intensities confirmed a partial defect in entrapment with relatively lower fraction of Orai1 present inside Stim1 puncta in STX11-depleted cells (Figure 7E–F). To determine whether reduced clustering of Orai1 resulted from structural defects within ER-PM junctions, we reversed the direction of fluorescent tags on Orai1 as well as Stim1 and repeated the experiment from Figure 7A–F in Figure 7G–L, using TIRF microscopy. Interestingly, the defect in Stim1-mediated entrapment of Orai1 could be rescued (Figure 7G–L) but SOCE remained significantly inhibited despite co-overexpression of Orai and Stim (Figure 7M–N; Peinelt et al., 2006; Mercer et al., 2006; Soboloff et al., 2006), suggesting ‘nonfunctional’ co-clustering of Orai1 and Stim1. We further assessed binding of full-length YFP-Stim1 to mutant R289A_E272A_E275A_E278A Orai1-CFP and found that Stim1 was able to co-cluster with mutant Orai1 (Figure 7—figure supplement 1A–D).

**Figure 7—figure supplement 1.**
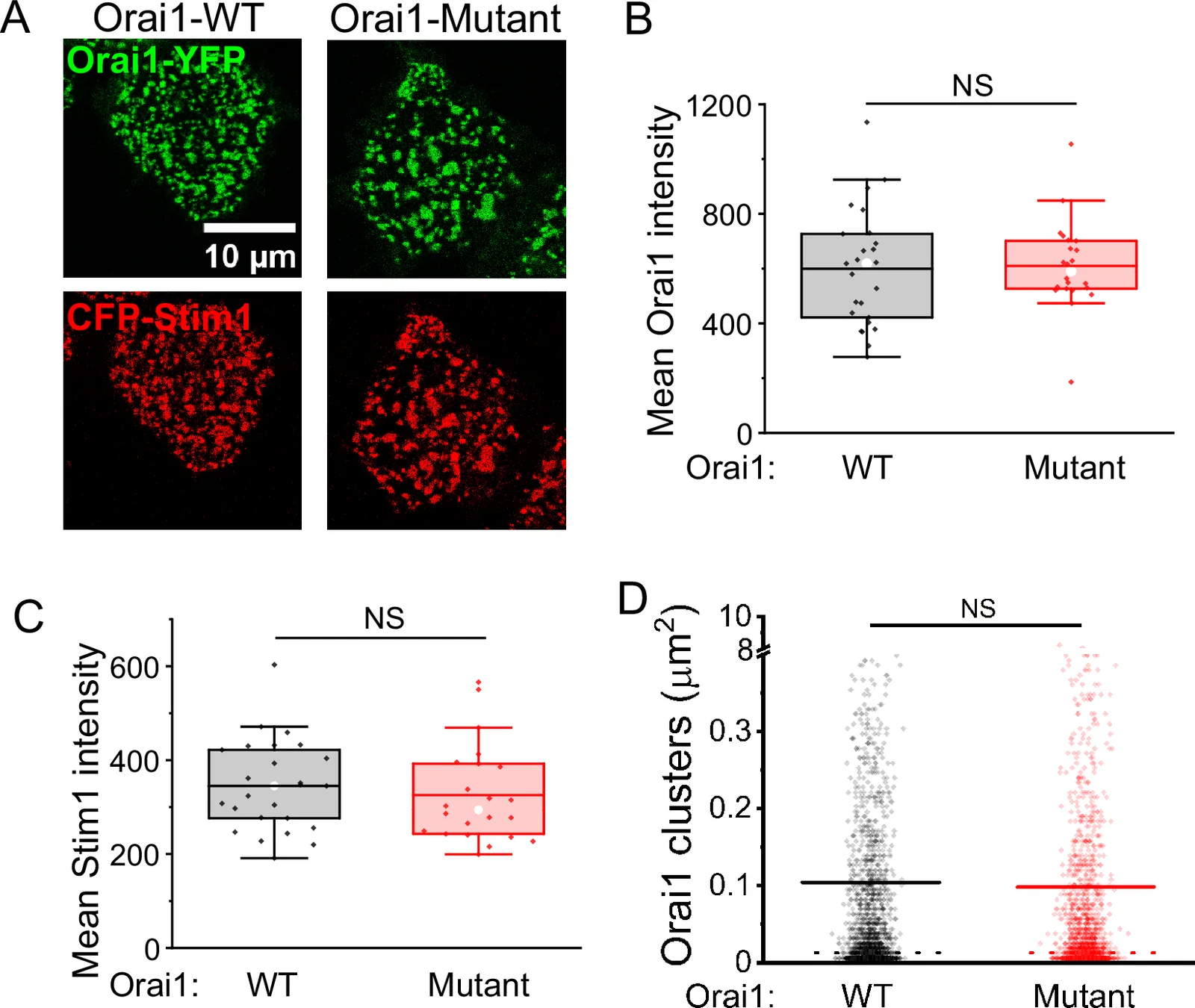
Measurement of co-clustering of mutant Orai1 with Stim1. (**A–D**) Representative confocal images (**A**) and quantification (**B–D**) of C-terminally YFP-tagged wild-type or R289A_E272A_E275A_E278A mutant Orai1 and CFP-Stim1 intensities and area of clusters in store-depleted HEK293 cells. n=20. Figure 7—figure supplement 1—source data 1.Numerical data underlying the quantitative analyses represented in Figure 7—figure supplement 1B–D.

Stim1 can itself serve as a marker of ER-PM junctions in store-depleted cells and quantification of area and mean intensity of Stim1-YFP or CFP-Stim1 puncta showed no defect in STX11-depleted cells imaged using confocal (Figure 7C and D) or TIRF microscopy (Figure 7H and I). To still assess whether STX11 depletion altered the structure and/or proximity of junctional ER to PM, we expressed an alternate marker of ER-PM junctions, GFP-MAPPER, in STX11-depleted cells (Chang et al., 2017). Quantification of the area and intensity of GFP-MAPPER puncta imaged using TIRF microscopy also did not show any significant difference in store-depleted, STX11 shRNA-treated HEK293 cells (Figure 7O–Q). Collectively, these data show that STX11 binding to resting Orai1 has implications for CRAC channel function beyond mere co-entrapment by Stim1.

**Figure 8.**
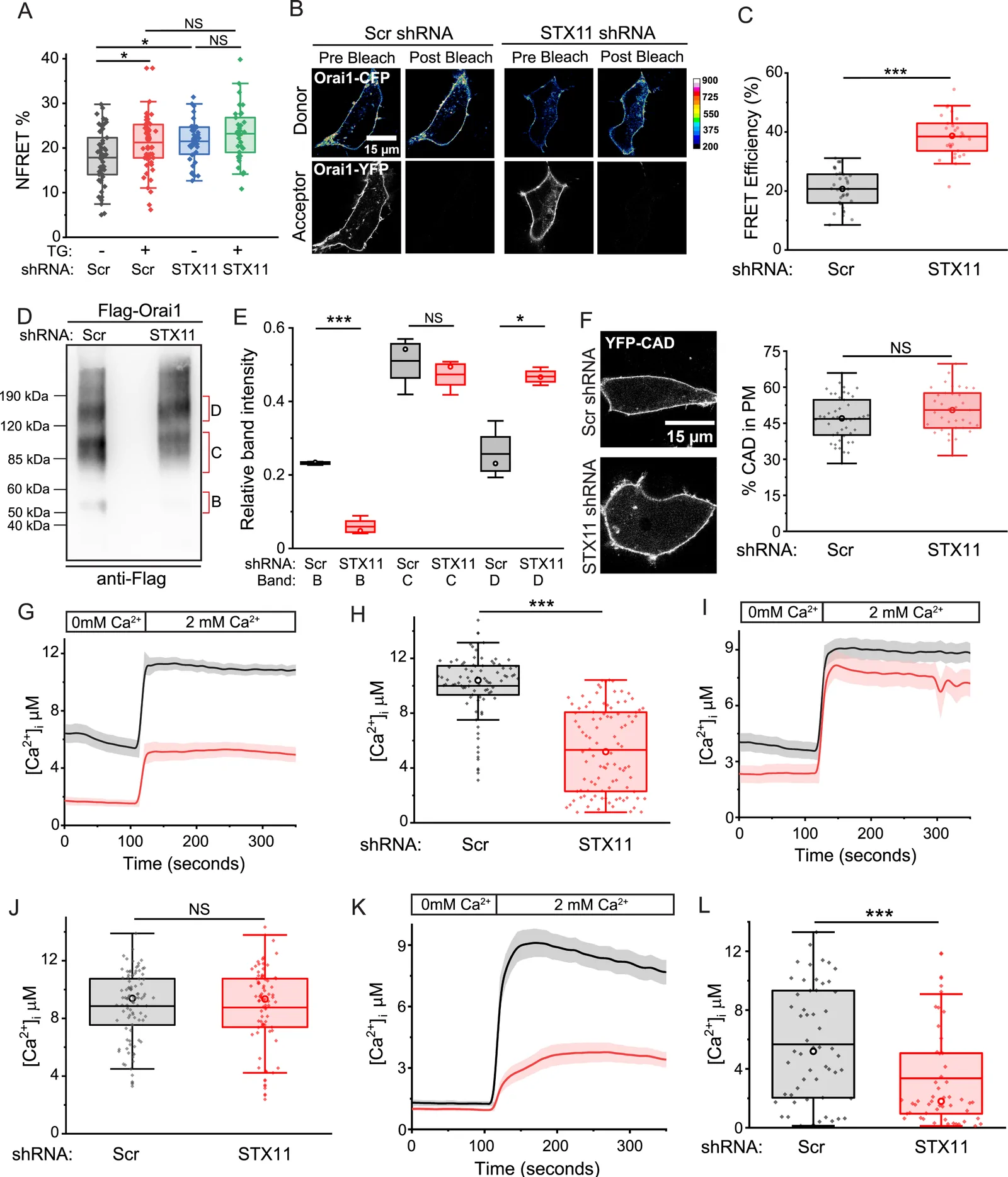
STX11 primes Orai1 for optimal on-site assembly. Oligomerization of Orai1. (**A**) N-FRET analysis of Scr and STX11 shRNA-treated HEK293 cells co-expressing Orai1-CFP and Orai1-YFP before and after store-depletion. (**B–C**) Representative images (**B**) and quantification (**C**) of FRET analysis using acceptor photobleaching of Scr and STX11 shRNA-treated Orai1, Stim1 double knockdown resting HEK293 cells co-expressing Orai1-CFP (donor) and Orai1-YFP (acceptor). (**D–E**) Representative western blot (**D**) and quantification of band intensities from repeats (**E**) showing distribution of BS3 crosslinked Flag-Orai1 oligomeric bands B,C,D in control and STX11-depleted HEK293 cells. N=3. (**F**) Representative confocal images and quantification (right) of YFP-CAD localization in Scr and STX11 shRNA-treated HEK293 cells expressing Orai1-CFP. (**G–H**) Fura-2 calcium imaging assay (**G**) and quantification of repeats (**H**) to measure constitutive calcium influx in Orai1-CFP and YFP-CAD expressing, control (black) or STX11-depleted (red) HEK293 cells. n=80–90, N=3 (**I–J**) Fura-2 calcium imaging assay (**I**) and quantification of repeats (**J**) to measure constitutive calcium influx in Orai1-H134S mutant expressing control (black) and STX11-depleted (red) HEK293 cells. n=80–100, N=3 (**K–L**) Fura-2 calcium imaging assay (**K**) and quantification of repeats (**L**) to measure constitutive calcium influx in Orai1-ANSGA mutant expressing control (black) and STX11-depleted (red) HEK293 cells. n=50–60, N=3. Figure 8—source data 1.Numerical data underlying the quantitative analyses represented in Figure 8A, C and E–L. Figure 8—source data 2.Folder containing labeled PDF files of the original western blots for Figure 8D. Figure 8—source data 3.Folder containing the original images of western blots shown in Figure 8D.

Defects in ‘functional’ entrapment of Orai1 suggest absence of a necessary molecular transition within Orai1 which precedes its interaction and gating by Stim1. To test whether STX11 induced structural changes within the CRAC channel pore subunit, Orai1, we co-expressed Orai1-CFP along with Orai1-YFP in Scr control or STX11-depleted HEK293 cells and measured Orai1:Orai1 FRET. We found significantly higher Orai1:Orai1 FRET in STX11-depleted resting cells in the absence of ectopically expressed Stim1 (Figure 8A). We have previously shown that store-depletion induces on-site assembly of Orai1 dimers into oligomers (Li et al., 2016). Notably, store-depletion induced a similar level of increase in Orai1:Orai1 FRET in Scr control cells. To confirm these findings, we performed acceptor photobleaching analysis on Orai1-CFP (donor), Orai1-YFP (acceptor) co-expressing HEK293 resting cells where we had stably knocked down the expression of endogenous Orai1 and Stim1. Although the trends were largely similar, the differences in Orai1:Orai1 FRET were much more significant in these cells (Figure 8B–C). These data demonstrate a previously unsuspected, novel molecular transition in Orai1 necessary for CRAC channel activation, which we define as ‘priming’. Further, we show that priming precedes the interaction of Orai1 with Stim1 in ER-PM junctions. To determine whether ‘priming’ simply induced a structural change in Orai1 protomers or facilitated correct assembly of Orai1 multimers, we expressed Flag-Orai1 in control or STX11-depleted resting cells, mechanically lysed to isolate membranes, crosslinked Orai1 oligomers using BS3 and resolved using SDS-PAGE (Figure 8D; Figure 8—figure supplement 1). We found a greater percentage of higher-order oligomers in STX11-depleted cells suggesting stoichiometric changes (Figure 8E).

**Figure 8—figure supplement 1.**
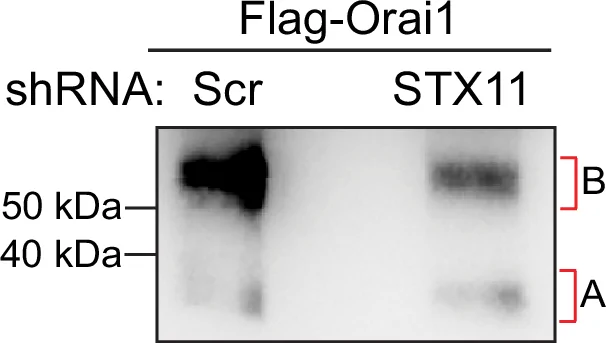
Lower half of the western blot of BS3 cross-linked Flag-Orai1 acquired at a higher exposure showing the monomeric Flag-Orai1 band (labeled as A). Figure 8—figure supplement 1—source data 1.Folder containing labeled PDF files of the original western blots for Figure 8—figure supplement 1A. Figure 8—figure supplement 1—source data 2.Folder containing the original images of western blots shown in Figure 8—figure supplement 1A.

**Figure 8—figure supplement 2.**
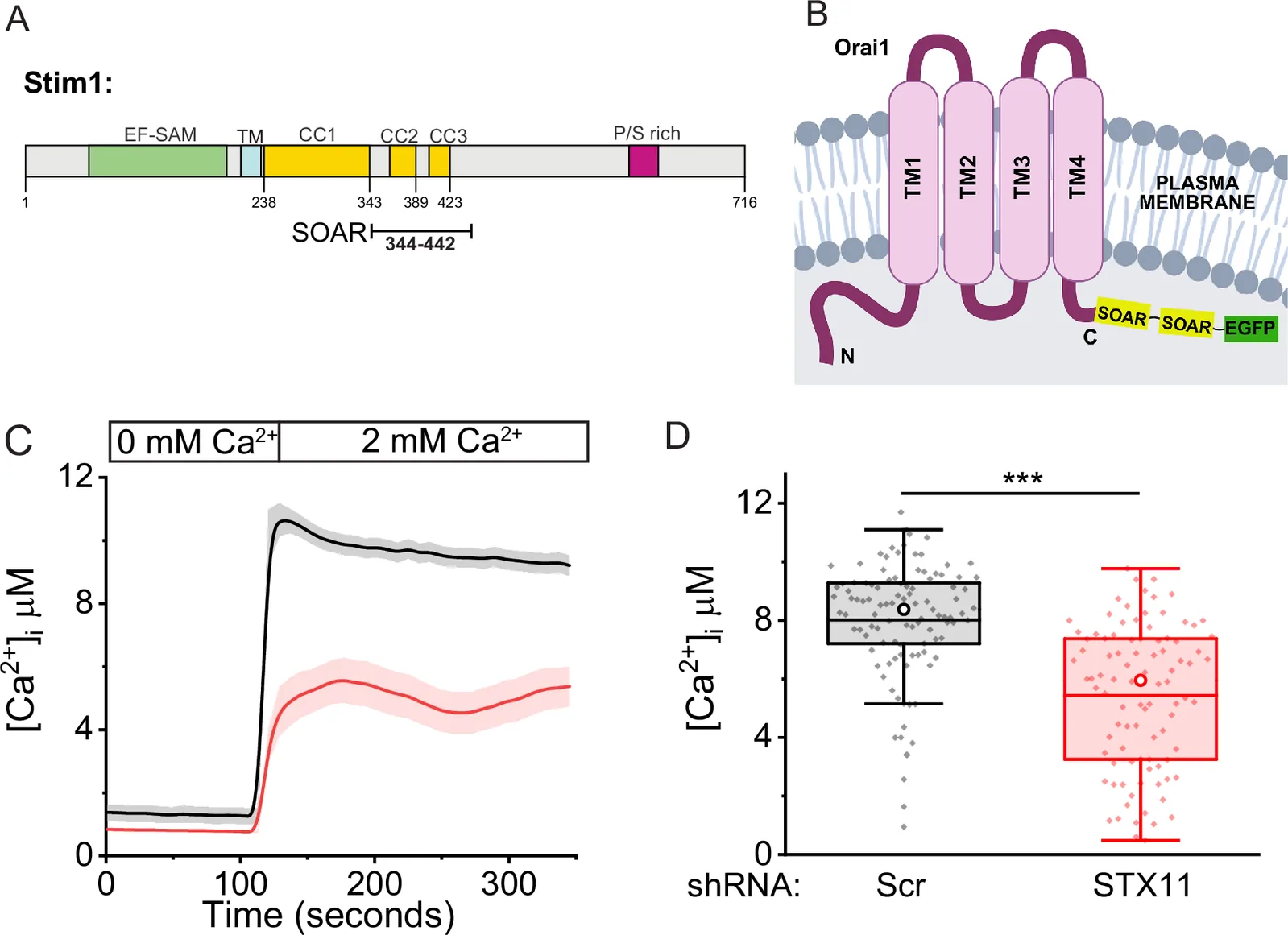
Measurement of activation of constitutive calcium influx by Orai1-SOAR-SOAR in STX11 depleted cells. (**A**) Schematic representation of SOAR domain in full-length Stim1. (**B**) Cartoon showing the design of the Orai1-SOAR-SOAR-EGFP (Orai1-S-S-GFP) construct. Created with BioRender.com. (**C**) Representative Fura-2 calcium imaging assay to measure constitutive calcium influx in Orai1-S-S-GFP expressing control (black) or STX11-depleted (red) HEK293 cells. Cells were imaged in Ringer’s buffer containing 0 mM followed by 2 mM extracellular Ca^2+^. (**D**) Quantification of constitutive calcium influx across experiments as shown in (**C**). n=90–100, N=3. Figure 8—figure supplement 2—source data 1.Numerical data underlying the quantitative analyses represented in Figure 8—figure supplement 2.

**Figure 8—figure supplement 3.**
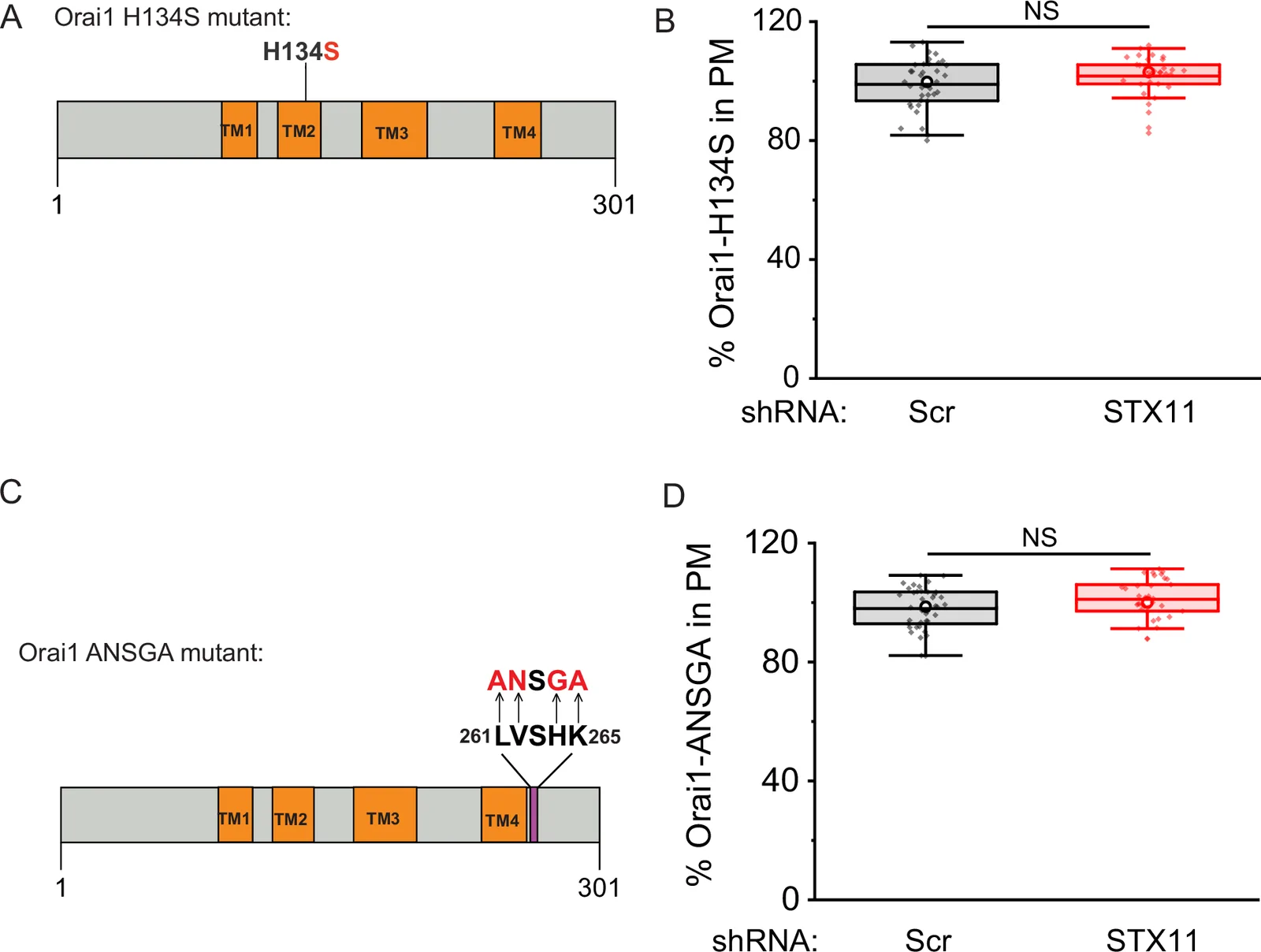
Schematic representation and quantification of constitutively active Orai1 mutants in the PM of STX11 depleted cells. (**A**) Schematic representation of H134S mutation in full-length Orai1. (**B**) Quantification showing percent Orai1-H134S-YFP intensity normalized to Orai1-WT-YFP in the PM of Scr and STX11 shRNA-treated HEK293 cells. n=30–40. (**C**) Schematic representation of ANSGA mutation in full-length Orai1. (**D**) Quantification showing percent Orai1-ANSGA-YFP intensity normalized to Orai1-WT-YFP in the PM of Scr and STX11 shRNA-treated HEK293 cells. n=30–40. Figure 8—figure supplement 3—source data 1.Numerical data underlying the quantitative analyses represented in Figure 8—figure supplement 3.

**Figure 9.**
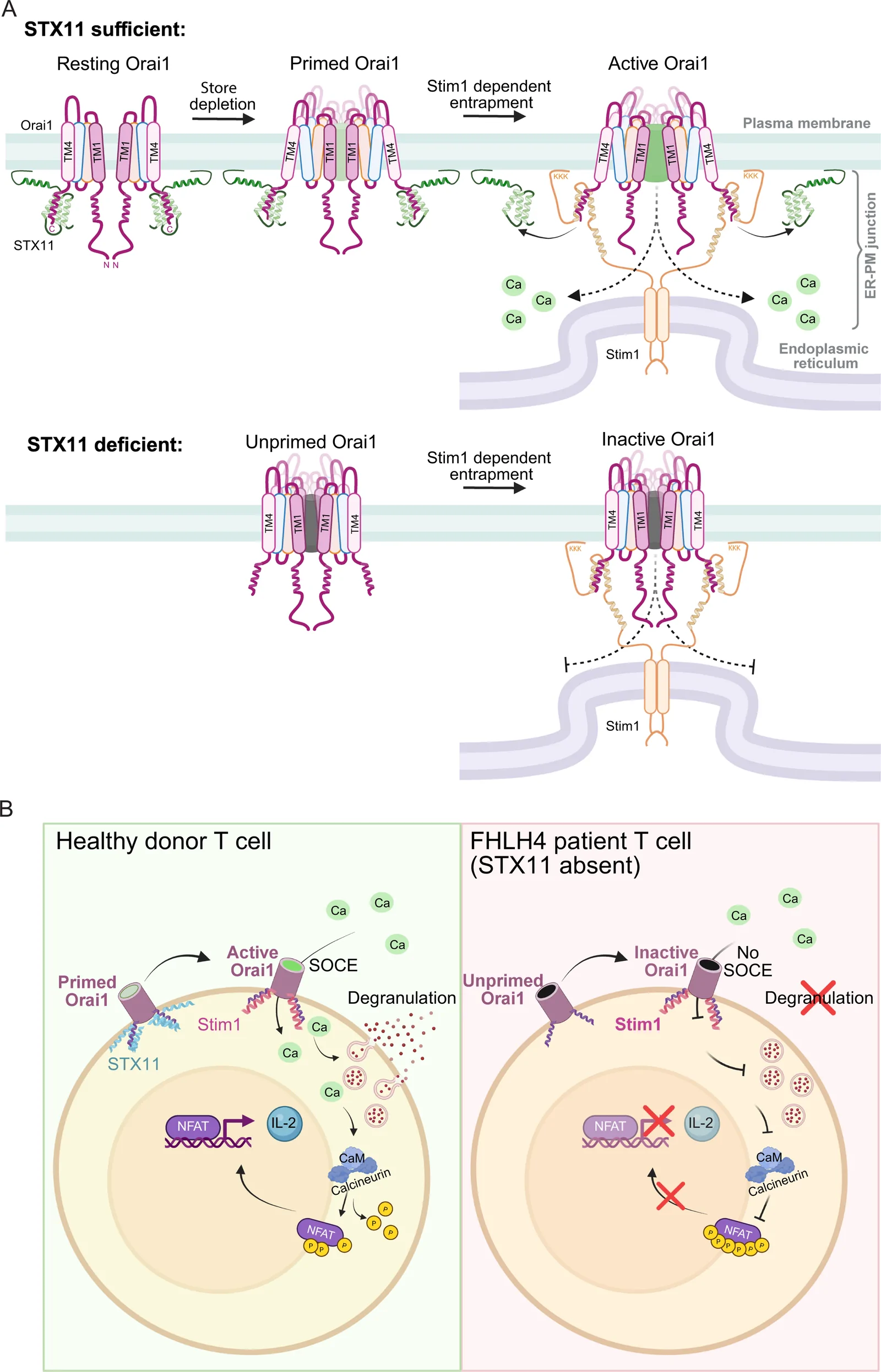
Models depicting STX11-mediated priming of Orai1 and its role in T cell signaling and function. (**A**) STX11 deficient (unprimed) and sufficient (primed) Orai1 in resting and store-depleted cells. For simplicity, STX11 interaction with only two subunits of Orai1 has been shown. Created with BioRender.com. (**B**) Regulation of T cell SOCE, NFAT activation, and degranulation by STX11 in healthy donor and FHLH4 patient T cell. Created with BioRender.com.

Increasing the density of Stim1 has been earlier proposed to bypass the requirements for accessory factors in SOCE. To determine whether increasing the levels of Stim1 could compensate for STX11 depletion, we expressed Orai1 tethered to two Stim-Orai activating region (SOAR) domains of Stim1 and GFP (Orai1-S-S-GFP; Figure 8—figure supplement 2A–B), which has been previously shown to constitutively activate Orai1 in HEK293 cells (Li et al., 2011). In STX11-depleted Orai1-S-S-GFP expressing cells, the magnitude of calcium entry was significantly smaller (Figure 8—figure supplement 2C–D). To determine whether reduced calcium entry resulted from a defect in the ability of SOAR to bind Orai1, we expressed YFP-CAD, in control or STX11-depleted Orai1-CFP expressing HEK293 cell line (Figure 8F). Quantification of PM localized YFP-CAD as a fraction of total CAD showed no defect in STX11-depleted cells. Yet, as seen with Orai1-S-S-GFP, calcium entry was significantly reduced (Figure 8G–H). We next expressed a constitutively active H134S mutant of Orai1 (Figure 8—figure supplement 3A) as it harbors an open pore and the C-terminal cytosolic tails of Orai1 are unlatched, pointing towards the cytosol (Hou et al., 2018). Remarkably, expression of H134S Orai1 completely restored constitutive calcium influx in STX11-depleted cells (Figure 8I–J) and showed similar PM expression when compared to wildtype Orai1 in all the groups (Figure 8—figure supplement 3B). To further establish whether binding with STX11 induces straightening of Orai1 tails or molecular transitions within transmembrane helices, we expressed in STX11-depleted cells the constitutively active ANSGA (261–265) mutant of Orai1 (Figure 8—figure supplement 3C), which harbors four consecutive mutations in the nexus region close to the Orai1 C-terminus resulting in the straightening of the C-terminal tail along with constitutive activation of CRAC currents (Zhou et al., 2016). The constitutive calcium entry in STX11-deficient Orai1 ANSGA mutant expressing cells was significantly suppressed (Figure 8K–L) even though the PM expression of the ANSGA Orai1 mutant was unchanged in STX11-depleted cells (Figure 8—figure supplement 3D). Taken together, these data show that STX11 induces a molecular switch in the pore-forming subunit, Orai1, which precedes and primes it for optimal on-site assembly into multimers and subsequent gating by Stim1. Such SNARE-dependent priming of Orai1 is crucial and cannot be compensated by merely increasing the density of Stim proteins bound to Orai or by introducing mutations that alter the orientation of Orai1 tails. Figure 9A and B show the proposed models depicting unprimed and primed Orai1 and its role in T cell function.

FHLH is a heterogeneous immune disorder where diverse genes such as *Prf1*, *STX11*, *UNC13D*, *Munc18-2*, *MAGT1, XIAP, LYST, AP3B1, RAB27A*, *ITK*, amongst several others, are broadly grouped together based on disease symptoms, although the age of onset and severity varies across the spectrum (Jessen et al., 2013; Chinn et al., 2018). Congenital immunodeficiency involving defects in the CTL and NK cell cytotoxicity appear to be the common factors underlying the development of FHLH, although the individual pathways involved are highly diverse. Using shRNA, we screened 6 additional genes, *Perforin1*, *RAB27A*, *UNC13D,* and *Munc18-2*, *Synaptotagmin11* and *13*, that have been either associated with vesicle fusion or routinely grouped together with STX11 in diagnosing FHLH. SOCE was not affected by depletion of most genes (Figure 1—figure supplement 3). Future studies will evaluate the role of others.

## Discussion

CRAC channels conduct a small, highly specific calcium current in response to the depletion of intracellular calcium stores. According to the prevailing view, Orai multimers reside in the PM and depend on the ER resident store sensor, Stim, for all structural transitions leading up to their activation (Prakriya and Lewis, 2015). Unlike other ion channels, the process of activation of CRAC currents is exceptionally slow. For instance, following break-in or store-depletion, on average, it takes ~5 min for Stims to cluster in the ER-PM junctions and for measurable CRAC currents to flow from Orai1. During this time, Stims undergo intramolecular transitions and slowly trap and gate freely diffusing Orai. It is, therefore, believed that no molecular transitions take place within the pore-forming subunit, Orai, until its entrapment by Stim in the ER-PM junctions.

We have shown that in STX11 deficient cells, the basal Orai1:Orai1 FRET is significantly higher, even in the absence of Stim1 or store-depletion. These data establish that Orai1 undergoes a hitherto unrecognized SNARE-dependent molecular transition, which precedes trapping and gating by Stim1. We have earlier shown that store-depletion induces dynamic on-site assembly of Orai1 dimers into oligomers (Li et al., 2016). In STX11-depleted resting cells, the ~25–50% increase over basal Orai:Orai FRET largely results from a change in the oligomerization of Orai1 because the proportion of BS3 crosslinked Orai1 oligomers was also found to be shifted toward higher molecular weight. It is very likely that a change in the overall conformation of Orai1 protomers facilitated this change in store-dependent Orai assembly and contributed to the overall change in Orai:Orai FRET. This conclusion is also supported by the observation that STX11 depletion induced a change in Orai:Orai FRET, which not only persisted but increased in Orai1, Stim1 double-deficient cells.

Furthermore, STX11 bound a region of Orai1 C-terminus that has been previously shown to bind Stim1. Therefore, STX11 induced molecular transitions in Orai are independent of, and precede its association and clustering by Stims. Because tagging of STX11 with fluorescent tags on either end results in mislocalization and degradation, our study could not demonstrate the real-time removal of STX11 from the Orai1- C-terminus to allow for Stim1 binding in live cells. It is also unclear whether binding or removal of STX11 induced the proposed molecular transition defined as ‘priming’ of Orai1. Further, it remains to be determined whether STX11 removal is actively induced by an additional chaperone or passive displacement due to formation of relatively higher avidity oligomeric associations between Orai1:Stim1 in ER-PM junctions. Future studies will be able to systematically evaluate each of these possibilities.

We have shown that the C-terminal cytosolic tail of Orai1 directly couples with STX11 in resting cells. Although binding appeared reasonably strong in co-IPs, the STX11:Orai1 interaction appeared somewhat weak in pull-downs. This is likely due, in part, to the instability of purified His-STX11 in solution, which necessitated shorter co-incubation times during pull-down assays to prevent precipitation of STX11. Instability could arise from the absence of a stabilizing co-chaperone, such as one of the known binding partners of SNAREs (Dulubova et al., 2007; Lee et al., 2020) or membranes to which STX11 has been proposed to attach in live cells. Together, these factors appear crucial for the stabilization of purified STX11 in vitro.

The individual relevance of Orai1 N- *versus* C-terminus in the trapping *versus* gating of Orai1 remains unclear (Park et al., 2009). Though crucial for trapping and gating, Orai1 tails were truncated in the early structures of *Drosophila* Orai (Hou et al., 2018). A previous NMR structure of truncated Orai1 C-terminal tails suggested that the tails of two adjacent Orai1 subunits bend, pair with each other in an antiparallel fashion, and sit closely apposed to PM (Stathopulos et al., 2013). However, in recent structures of constitutively active H134 mutant Orai1, the C-terminal tails were found to orient away from the membrane (Hou et al., 2018). Although C-terminal tails in ANSGA Orai1 mutant are also proposed to be unlatched, STX11 depletion inhibited ANSGA Orai1 induced constitutive calcium influx (Zhou et al., 2016). In the absence of direct structural evidence that establishes conformational similarity between constitutively active H134 *versus* ANSGA mutant Orai1, Orai1 +CAD and Orai1-S-S, it is likely that Orai1 can assume several different open conformations/assemblies (Li et al., 2016; Hou et al., 2018; Zhang et al., 2026; Lu et al., 2024; Augustynek et al., 2022). Since most mutants showed sensitivity to STX11 depletion, it reinforces our conclusions that STX11 induced conformational shifts precede gating and involve Orai1 TMs.

Using Fura-2 imaging assays in STX11-depleted cells, we have conclusively established that STX11 is necessary as well as limiting not only in live and unperturbed HEK293, U2OS, Jurkat, and primary T cells but also HEK293 cells co-overexpressing Orai1 and Stim1. Previous whole cell patch clamp analysis of HEK293 cells co-overexpressing Orai1 and Stim1 showed up to 50–100-fold amplification of CRAC currents, whereas Fura-2 based imaging of live cells routinely shows only twofold amplification (Peinelt et al., 2006; Mercer et al., 2006; Soboloff et al., 2006). The patch clamp studies performed on Stim, Orai over-expressing cells are often extrapolated to conclude that all other accessory factors that have since been identified are unnecessary for native CRAC currents. A major problem with this interpretation is that even though amplification of CRAC currents was shown, none of the previous patch clamp studies established whether higher currents resulted from a greater number of active channels or unchecked conductance per channel by performing single-channel recordings. Direct single-channel recordings of CRAC are challenging. As a result, estimates of open probability and channel number often rely on fluctuation (noise) analysis rather than direct measurements of single-channel events (Prakriya and Lewis, 2006). The fact that recording of I_CRAC_ in the whole cell configuration involves use of 20 mM extracellular calcium and dialysis of cells with high concentrations of BAPTA, a fast calcium chelator, the calcium-dependent inactivation of CRAC channels, a key mechanism of regulation of I_CRAC_ magnitude in live cells, is disrupted. Furthermore, hyperpolarizing potentials and voltage clamping used to record CRAC currents prevent the progressive depolarization of cells due to influx of cations which would otherwise dampen the driving force for CRAC currents in intact cells. Given these limitations, Fura-2 assays offer a more realistic and physiological reflection of SOCE amplitudes in live cells as they allow for these regulatory mechanisms, which are necessary for the regulation of native currents, to be still operational. While patch clamp is a direct method of verification of CRAC currents, exercising logic in the extrapolation of amplitudes of I_CRAC_ using patch clamp is imperative.

Most human mutations in the *STX11* coding region result in the complete loss or severe depletion of STX11 protein levels (Hellewell et al., 2014). The FHLH4 disease, therefore, results from reduced STX11 expression in most reported patients. Targeted mutagenesis of the STX11:Orai1 interface lining residues based on the AlphaFold 3 model, however, did not destabilize or mis-localize either N147A_E150A STX11 or R289A_E272A_E275A_E278A Orai1, and yet both mutants showed reduced binding to their respective wildtype counterparts. Importantly, the R289A_E272A_E275A_E278A mutant of Orai1 retained the ability to bind and cluster with Stim1.

Most importantly, we have shown that deficiency of a Q-SNARE, STX11, causes a vesicle trafficking independent, severe defect in SOCE, IL-2 gene expression, and degranulation in FHLH4 T cells. Mechanistically, STX11 directly binds and induces a molecular switch in Orai1 necessary for its assembly into ‘functional’ multimers which we call a ‘primed’ state. We suggest that ion channel priming is a novel, direct, and primary role of specific SNAREs, independent of vesicle trafficking. In accordance with this hypothesis, early SNARE-like proteins have been found in bacteria, which lack an endomembrane system but express a variety of ion channels (Delevoye et al., 2004; Paumet et al., 2009; Barocchi et al., 2005). SNAREs are now thought to have evolved from a common archaeal precursor found in the genomes of Asgard and members of *Legionella* (Neveu et al., 2020).

Analyses of FHLH4 patient T cells suggest that SOCE defects significantly contribute to the pathogenesis of FHLH4 disease. We, therefore, propose that pharmacological induction or promotion of the primed intermediate state of Orai1 could potentially provide an alternative to immunosuppressants and bone marrow transplants for FHLH4 as well as SCID patients, with minimal risk of autoimmunity.

## Materials and methods

### Plasmid constructs and transfection

Stim1 CAD was subcloned in eYFPC1 vector after amplification from YFP-Stim1 plasmid to generate YFP-CAD. pLKO.1, psPAX2, and pMD2.G were purchased from Addgene. pLKO.1 cloned shRNA sequences targeting the genes of interest were either purchased from Horizon Discovery, UK, or designed and cloned in-house using the Broad Institute portal (https://portals.broadinstitute.org/gpp/public/gene/search). Full-length human STX11 was cloned from human cDNA prepared from HEK293 cells and subcloned into pcDNA3.1(+), pcDNA/4TO/Myc-HisA (Invitrogen, Grand Island, NY), pMSCV-IRES-mcherry (Addgene), pEF1alpha-IRES (Clontech), and pET28b vector (Ramanagoudr-Bhojappa et al., 2021) with an N-terminal 6XHis-tag. The fragments of STX11 were cloned in pET28b vector with an N-terminal 6xHis tag for expression in *E. coli*. HA tag was inserted between H30 and G31 in STX11 cloned in pMSCV-IRES-mCherry construct using PCR. H134S and ANSGA mutants of Orai1 were generated using site-directed mutagenesis and PCR, respectively. The fragments of Orai1 (1–87 and 256–301) were amplified from full-length constructs and cloned into pMAL-c5X vector (New England Biolabs), in-frame with MBP protein coding sequence, as described previously (Ramanagoudr-Bhojappa et al., 2021). SNAP23, SNAP25, and SNAP29 cDNA cloned in pCMV-Sport6.1 vectors were purchased from Dharmacon and subcloned into pcDNA4/TO/Myc-HisA. SNAP23 was subcloned in pMAL-c5X vector in-frame with MBP. The lists of all the primers, shRNAs, and plasmids used in this study can be found in Supplementary files 1 and 2. All plasmid DNA transfections in human cell lines and primary cells were done using Lipofectamine 2000 (Invitrogen)/ Lipofectamine 3000 (Invitrogen) or Amaxa nucleofection kit (Lonza, Basel, Switzerland), respectively, as per manufacturer’s protocol.

### Cell lines

HEK293-FT cells were cultured in high glucose DMEM with 10% FBS and 10 mM HEPES, 1 X Penicillin-Streptomycin, 1 X GlutaMax, and 1 X non-essential amino acid and transfected with the appropriate plasmids to generate viral supernatants. Lentiviral shRNA transduction experiments were performed in HEK293, U2OS, or Jurkat (ATCC, Manassas, VA) cell lines cultured in low glucose DMEM (Hyclone, Logan, UT) or RPMI (Hyclone), respectively, with 5–10% fetal bovine serum (Hyclone), 1 X Penicillin-Streptomycin, and GlutaMax (Gibco, Grand Island, NY). Stable cell lines generated using HEK293 and U2OS parent lines have been described previously (Miao et al., 2013). To generate HEK293 stable cell line expressing Orai1-BBS-YFP construct, the cells were nucleofected (SE Cell Line 4D-Nucleofector X kit_Amaxa _V4XC) and YFP positive cells were sorted. The identity of cell lines was verified using STR profiling from ATCC. They were tested for mycoplasma contamination twice every year and found to be negative.

### Lentiviral transductions

For the generation of lentiviral supernatants, shRNAs cloned in pLKO.1 were co-transfected with psPAX2 packaging and pMD2.G envelope plasmids into HEK293-FT cells using the calcium phosphate method of transfection. 48- and 72 hr post-transfection, viral supernatants were collected, pooled, and stored at –80 °C till further use. For transduction of HEK293 and Jurkat cells, 0.1 million cells were plated in 6- or 24-well plates either the day before (HEK293) or the same day (Jurkat). Viral supernatants were added to the cells along with 8 μg/ml polybrene, and cells were spun at 2500 RPM, 30 °C for 90 min. 24 hr or 48 hr post-spinfection, puromycin was added at a final concentration of 1 μg/ml to HEK293 and Jurkat cells, respectively. Cells were analyzed 3–4 days post transduction.

### Immunocytochemistry and confocal imaging

For STX11 localization, resting or store-depleted (1 μM TG) WT HEK293 or HEK293 cells stably expressing Orai1-YFP were either plated in 6 well plates and spinfected with viral supernatants generated from pMSCV-STX11(HA)-IRES-mCherry, pMSCV-IRES-mCherry (empty vector control), or plated in carbon-coated, glow-discharged 35 mm glass-bottom dishes and transfected with pEF1alpha-STX11-IRES-mCherry. 24 hr post-spinfection, cells were plated in 35 mm glass-bottom dishes. For immunolabeling, cells were washed with Ringer’s buffer, fixed with 4% PFA, and blocked with 3% BSA containing 0.1% NP40 for 1.5 hrs. Post-fixation and permeabilization, cells were incubated either with anti-HA (CST) or rabbit anti-STX11 (SySy/ Proteintech) primary antibodies O/N at 4 °C, washed, and incubated with either Alpaca VHH anti-rabbit AF488, Donkey anti-rabbit A647, or Goat anti-rabbit A647 secondary antibodies for 1 hr at room temperature (RT). Cells were counterstained with DAPI for 10 min at RT and imaged using an Olympus FV3000 laser scanning confocal microscope. Images were acquired sequentially using the following parameters: DAPI (DM405/488 dichroic, 405 nm excitation, 430–470 nm emission); anti-HA Alexa488 (DM405/488, 488 nm excitation, 500–590 nm emission); anti-STX11 (DM405/488/561/640, 640 nm excitation, 650–750 nm emission); Orai1-YFP (DM405/488/561/640, 488 nm excitation, 521–591 nm emission); CFP-Stim1 (DM405-445/514/594, 405 nm excitation, 448–510 nm emission). For STX11-N147A-E150A mutant, HEK293 cells were spinfected with viral supernatants and imaged as described above, where WGA (Wheat germ agglutinin) conjugated with Alexa 488 was used to mark the plasma membrane. For immunolabeling of ER and Golgi, HEK293 cells were treated with Scr or STX11 shRNA, washed, fixed, blocked, and incubated with anti-KDEL and anti-GM130 primary antibodies overnight at 4 °C. Following incubation, the cells were washed with 1 X PBS containing 0.1% NP40, incubated with Donkey anti-mouse A647 secondary antibody for 30 min at RT, washed, and counterstained with DAPI for 10 min. Images were acquired using an Olympus FV3000 laser scanning microscope as mentioned above.

### Quantification of Orai1 and Stim1 intensities inside/outside ER-PM puncta

HEK293 cells stably or transiently expressing wildtype Orai1-YFP or E272A-E275A-E278A-E289A Orai1-YFP with CFP-Stim1 or CFP-Orai1 with Stim1-YFP were plated in 6 well plates and transduced with scramble or STX11 shRNA. 48 hr post transduction, cells were trypsinized and plated in carbon-coated 35 mm dishes. Images were acquired using the TIRF microscope setup described before (Miao et al., 2013). Briefly, resting cells were imaged first, and positions were marked. Store-depleted images were acquired after incubation with 1 μM TG and 10 mM EGTA for ~6 mins. Stim1 and Orai1 clusters were defined using Otsu local thresholding, and Pearson’s correlation coefficient was measured using colocalization finder in ImageJ software. Stim1 and Orai1 intensity, area in µm^2^, and Pearson’s correlation coefficient values obtained from the analysis were plotted using Origin software.

### Orai1:Orai1 FRET

Scramble and STX11 shRNA transduced HEK293 cells were plated on freshly carbon coated, glow discharged 35 mm glass bottom dishes and transfected with 0.5 μg each of Orai1-CFP and Orai1-YPF plasmid DNA using Lipofectamine 3000. 16 hr post transfection; co-transfected cells were imaged using PlanApoN 60XO/1.42 NA objective with Olympus FV3000 laser scanning microscope. Orai1-CFP, Orai1-YFP, and FRET images were acquired with the following excitation laser and emission bandwidth parameters using DM405-445/514 dichroic mirror: CFP Ex-405nm Em-460–630 nm; YFP Ex-514nm Em-530–630 nm; FRET Ex-405nm, Em-530–630 nm. ROIs representing the plasma membrane rim were drawn around each cell, followed by local thresholding using Bernsen algorithm in Fiji software to generate an image mask which was applied to CFP, YFP, and FRET images to extract mean intensity values. The background subtracted mean intensities of FRET (I_FRET_), CFP (I_Donor_), and YFP(I_acceptor_) images were measured for each cell. Cells singly expressing Orai1-CFP or Orai1-YFP were used as CFP donor or YFP acceptor channel bleed-through (BT) controls. The BT was calculated using the following formula:

$\rm BT_{Donor}=\frac{I_{FRET}}{I_{Donor}}$ when the Orai1-CFP donor alone is expressed,

$\rm BT_{acceptor}=\frac{I_{FRET}}{I_{acceptor}}$ when the Orai1-YFP acceptor alone is expressed.

FRET and NFRET percentage was calculated by subtracting the contributions of CFP and YFP bleed-through from the FRET signal using the following:

$$\displaystyle FRET=I_{FRET}-\left (BT_{Donor}*I_{Donor}\right)-\left (BT_{Acceptor}*I_{Acceptor}\right)$$

$$\displaystyle NFRET\%=\left (\frac{FRET}{\sqrt{\left (I_{Acceptor}*I_{Donor}\right)}}\right)*100$$

### Acceptor photobleaching

Scramble and STX11 shRNA transduced, Orai1, Stim1 double knockdown HEK293 cells were plated on Poly-D-Lysine coated glass bottom dishes (Cellvis) and transfected with 0.5 μg DNA each of Orai1-CFP and Orai1-YPF using Lipofectamine 2000. 16 hr post transfection; the cells were imaged using PlanApoN 60XO/1.42 NA objective with Olympus FV3000 laser scanning microscope. Images were acquired with the following excitation laser and emission bandwidth parameters using DM405-445/514 dichroic mirror: CFP Ex-405nm Em-460–508 nm; YFP Ex-514nm Em-530–630 nm, with 4% offset. The pre-bleach images of Orai1-CFP (the donor) and Orai1-YFP (the acceptor) were captured sequentially. This was followed by bleaching of the acceptor, Orai1-YFP, using 100% 514 laser power for 2 min and then acquiring the donor and acceptor images post-bleach. ROIs were drawn around the cell periphery using the Orai1-CFP image. Local thresholding was performed using Bernsen algorithm to generate binary masks, which were then applied to the CFP images to measure mean intensities. The background subtracted mean intensities of CFP images were measured for each cell, and the FRET efficiencies were calculated as follows:

$$\displaystyle E\%=\left (1-\frac{I_{pre}}{I_{post}}\right)*100$$

I_pre_ and I_post_ denote the average fluorescence intensity of the donor in the plasma membrane before and after acceptor photobleaching.

### Alpha-bungarotoxin binding assay

U2OS or HEK293 cell lines stably expressing Orai1-BBS-YFP construct were either transduced with Scr or STX11 shRNA and harvested 3 days post-transduction. Jurkat cells treated with Scr and STX11 shRNA were transiently transduced with pMSCV-Orai1-BBS-YFP 48 hr prior to harvest. For surface labeling, cells were incubated on ice with AF647 conjugated alpha-bungarotoxin (BTX-A647) (1 μg/ml) dissolved in 1 X HBSS with 2% FBS for 30 min, washed, and fixed with 4% PFA (~20 min at RT). For flow-cytometry, U2OS and HEK293 cells were analyzed on a Cytoflex FACS analyzer (Beckman Coulter), and data were analyzed using Flow Jo software. For microscopy, the shRNA-treated HEK293 cells were plated in 35 mm glass-bottom dishes 1 day prior to harvest, whereas labeled Jurkat cells were plated on poly-D-lysine coated coverslips, mounted onto glass slides, and sealed. Imaging was performed using an Olympus FV3000 confocal microscope.

Line profiles of Orai1-YFP and BTX-A647 were drawn on raw confocal images of HEK293 cells. Quantifications of Orai1-YFP and BTX-A647 were calculated using the following formula:

$$\displaystyle \%Orai1-YFP\,or\, BTX-A647\,in\, the\,PM=\left (\frac{PM\,Intensity}{Total\,Intensity}\right)*100$$

### Alkaline phosphatase (AP) assay

HEK293 cells were transduced with Scr and STX11 shRNA, and 48 hr post-transduction, the cells were transfected with pAP tag plasmid (Genhunter) in low glucose DMEM without phenol red. Supernatants from control and transfected cells were collected at 48 hr post-transfection, and triplicates of undiluted supernatant or those diluted at 1:1, 1:2, 1:4 using DMEM without phenol red were added to 96-well plates and mixed in a 1:1 ratio with 1 mg/ml pNPP (Disodium-4-Nitrophenyl Phosphate Hexahydrate) substrate in AP Assay Buffer (100 mM Tris-Cl pH-9.0, 100 mM NaCl, and 5 mM MgCl_2_) to obtain final AP:pNPP ratios of 1:1, 1:2, 1:4, and 1:8. Supernatants from untransfected cells were used as blank controls. After 25 min of incubation at 37 °C in the dark, the assay was terminated by adding 0.4 M NaOH, and absorbance at 405 nm was measured using Softmax Pro version 5.4 in FlexStation 3.

### Measurement of GFP-Mapper cluster size and intensity

HEK293 cells treated with Scr and STX11 shRNA were transfected with GFP-Mapper plasmid 48 hr post-transductions. The cells were store-depleted with 1 μM TG and 10 mM EGTA for ∼6 min, fixed with 4% PFA, washed, and imaged using TIRF microscopy. To analyze the images, local thresholding was performed in ImageJ using the Bernsen algorithm, and the resulting mean intensity and area (μm^2^) of the GFP-Mapper clusters were plotted using Origin software.

### Validation of STX11 knockdown using qPCR

Scramble and STX11 shRNA-treated HEK293 and Jurkat T cells were counted, and an equal number of cells were used for lysis. The lysates were homogenized using QIA shredder columns (Qiagen), and RNA isolation was done using RNeasy Plus Mini kit (Qiagen). Total RNA isolated from both groups was used for cDNA synthesis using oligo dT primers and Superscript IV as per manufacturer’s guidelines. The concentration of synthesized cDNA was estimated using Qubit ssDNA assay kit, and STX11 TaqMan probes were used to perform the qPCR using LightCycler 96 (Roche). Beta-actin and RPL30 were used as housekeeping controls.

### Single cell Ca^2+^ imaging

Cells were plated in carbon-coated glass bottom dishes (one day prior or 30 mins before the assay for HEK293, U2OS, and Jurkat T cells respectively) and loaded with 1 μM Fura-2 AM dye in HBSS (CaCl_2_ 1.8 mM, KCl 5.36 mM, MgSO_4_ 0.81 mM, NaCl 136.89 mM, Na_2_HPO4 0.335 mM, D-Glucose 5.55 mM) for 30 mins, at 37 °C in the dark. After incubation, cells were washed and incubated in Ringer’s buffer (135 mM NaCl, 5 mM KCl, 1.8 mM CaCl_2_, 1 mM MgCl_2_, 5.6 mM Glucose, 10 mM HEPES) for an additional 10 mins, washed, and imaged in Ringer’s buffer or Calcium-free Ringer’s buffer (135 mM NaCl, 5 mM KCl, 1 mM MgCl_2_, 5.6 mM Glucose, 10 mM HEPES at pH 7.5), as indicated. Olympus IX-71 inverted microscope equipped with a Lamda-LS illuminator (Sutter Instrument), Fura-2 (340/380) filter set (Chroma, Bellows Falls, VT), a 10X0.3 NA objective lens (Olympus, UPLFLN), and a Photometrics Coolsnap HQ2 CCD camera was used to capture images at a frequency of ∼1 image pair every 2- or 4 s interval. Data were acquired, analyzed, and plotted using MetaFluor (Molecular Devices), Microsoft Excel, and Origin software. Approximately 30–40 cells were imaged per group in each experiment unless otherwise stated. In-vitro calibration of Fura-2 was performed using a calibration kit (Molecular Probes_F6774) according to the manufacturer’s protocol. Briefly, calcium calibration buffers spanning a range of free calcium concentrations (0, 0.017, 0.038, 0.065, 0.1, 0.15, 0.225, 0.351, 0.602, 1.35, and 39 µM) and containing 15 µm microsphere beads were loaded onto cleaned glass slides, sealed with a coverslip, and imaged. A standard linear calibration curve was constructed by plotting the 340/380 ratios (y-axis) against the known free calcium concentrations (x-axis) and used to quantify the absolute intracellular free calcium concentrations using a simple linear regression equation y=mx + c.

### Quantification of SOCE

Quantification of mean SOCE ± SE from three independent experiments was performed as follows. The mean of 5 peak amplitude values after the addition of 2 mM calcium from one of the Scr shRNA groups, amongst the three experiments, was normalized to the baseline values (5 values just before the addition of 2 mM calcium) and was set at 100%. The relative percent responses of all other Scr and test shRNA groups were normalized with respect to this group.

### Constitutive calcium influx assay

For constitutive calcium influx assay, shRNA-treated HEK293 cells were either transfected or nucleofected with Orai1-S-S-EGFP, Orai1(H134S)-YFP, or Orai1(ANSGA)-YFP and ~12–14 hr post transfection (~4–5 hr post nucleofection), cells were loaded with Fura-2 AM, washed, and imaged in Ringer’s buffer with 0 mM calcium to acquire baselines and 2 mM calcium thereafter. To measure CAD induced constitutive calcium influx, Orai1-CFP expressing stable HEK293 cells were nucleofected with YFP-CAD and analyzed ~4 hr later as described above. To measure CAD induced calcium influx in E272A-E275A-E278A-R289A mutant Orai1-YFP expressing cells, Orai1-shRNA-treated HEK293 cells were transfected with wildtype or mutant Orai1-YFP constructs along with YFP-CAD. To identify cells expressing Orai1 mutants and fusion proteins in the plasma membrane, cells were imaged using a 20X0.7 NA water objective lens (Olympus, UApoN340, Japan). Images were acquired at a frequency of ∼1 image pair every 10 s interval to avoid photobleaching and analyzed as described above. Quantification of constitutive calcium influx using Orai1 mutants was done by plotting the average of five consecutive data points immediately following the addition of 2 mM calcium across three independent repeats. Fura 2 calibration was done separately for these experiments as described above.

### Transferrin recycling assay

HEK293 and Jurkat T cells treated with Scr or STX11 shRNA were plated on 35 mm glass-bottom dishes and washed three times with serum-free DMEM to remove residual FBS. The cells were then incubated with Alexa Fluor 647-conjugated human holo transferrin (20 μg/ml) in serum-free DMEM for 30 min at 37 °C (pulse), placed on ice for 5 min and subjected to three rapid washes with acidified ice-cold serum-free DMEM (pH 3.5–5.5) to remove transferrin bound to cell surface receptors. Cells were then chased for indicated time points at 37 °C, washed with HBSS, fixed with 4% PFA, washed again with PBS, and imaged using an IX83 widefield microscope equipped with a 60 X/1.25 NA objective lens (Olympus UPFLN). Fluorescence images were collected on a Hamamatsu Orca-Fusion camera using the same settings across all conditions. Brightfield images were used to define cell boundaries, and per-cell mean fluorescence intensities were quantified using ImageJ software.

### Dextran-FITC uptake assay

HEK293 cells treated with Scr or STX11 shRNA were plated on 35 mm glass-bottom dishes, washed with serum-free DMEM, and incubated with FITC-conjugated dextran (1 mg/ml) in serum-free DMEM for 30 min at 37 °C. The cells were then washed with HBSS, fixed with 4% PFA, and imaged. To preserve FITC fluorescence post-fixation, the cells were treated with 20 mM ammonium chloride prior to imaging. Images were acquired and analyzed as described above for Transferrin recycling assay.

### Measurement of NFAT nuclear localization by western blot

Jurkat T cells were transduced with scramble or STX11 shRNA to knockdown STX11 as described above. On day 4 post-transduction, cells were collected, spun down, and resuspended in plain RPMI media and rested for 1 hr at 37 °C. Following this, cells were counted and divided into two equal groups. One group was resuspended in RPMI (unstimulated), and the other in RPMI media containing 1 μM TG +10 ng/ml PMA (Phorbol Myristate Acetate) (stimulated) and incubated at 37 °C for 30 mins. Following incubation, cells were pelleted and the nuclear and cytosolic protein fractions were separated using NE-PER kit according to the manufacturer’s guidelines and subjected to SDS-PAGE and western blot using the mouse anti-NFATc2 primary antibody (Santa Cruz) followed by HRP-conjugated Donkey anti-mouse secondary antibody.

### Estimation of nuclear translocation of NFAT by immunolabeling

Control and STX11-depleted Jurkat T cells were rested in plain RPMI for ~1 hr prior to the assay. ~100,000 cells per group were plated on freshly carbon-coated coverslips for 40 min and stimulated for 1 hr at 37 °C with 5 μg/ml anti-CD3 antibody, diluted in plain RPMI. Following this, cells were washed and fixed using 4% PFA diluted in 1 X PBS for 20 min at RT, washed twice and incubated with 30 mM Glycine for 10 min at RT. For permeabilization and blocking, cells were incubated with 0.1% Saponin, 3% BSA diluted in 1 X PBS for 1 hr at RT, washed and incubated with anti-NFAT primary antibody (anti-NFAT1, CST) at 4 °C, overnight. Following primary antibody application, cells were washed with 1 X PBS and incubated with anti-Rabbit AF647 secondary antibody for 1 hr, washed and stained with DAPI for 5 min followed by additional washes. Images were acquired in the FV3000 confocal microscope using a ×100 objective lens. DAPI was used to identify the nuclear area. Nuclear *versus* whole cell (total) NFAT mean intensity ratio was plotted across different groups.

### Whole cell lysates, western blot and co-immunoprecipitation

HEK293 cells transfected with the desired plasmids were lysed using buffer containing 50 mM Tris-Cl (pH 8.0), 150 mM NaCl, 1% NP-40, 1 mM PMSF, and protease inhibitor cocktail. The WCLs were centrifuged at 21,000 × *g* for 15 min and supernatants were subjected to SDS-PAGE and western blot. For immunoprecipitations, lysates were divided into two equal parts. To the first tube, the appropriate anti-mouse or anti-rabbit primary antibody was added and to the second, the same amount of the respective IgG control antibody was added. The antibody-lysate mixtures were incubated overnight at 4 °C. Following this, Protein A/G Mag Sepharose beads were added and incubated with the antibody-lysate mixtures for 4 hr, washed with the lysis buffer containing 0.1% NP-40, boiled with 1 X Laemmli buffer +120 mM DTT and subjected to SDS-PAGE and western blot analysis. Typically, 1/10^th^ of the WCL was loaded in the input lane of the co-IP blots.

### *E. coli* expression and in vitro binding assays

Full-length His_6_-tagged STX11 and truncation mutants were cloned in pET28b, expressed in Lemo21 (DE3) *E. coli* cells and induced with 1 mM IPTG (Isopropyl β- d-1-thiogalactopyranoside) for 14–18 hr at 18 °C. The cell pellets were lysed in buffer containing 50 mM Tris-Cl (pH 8.0), 150 mM NaCl, 10% Glycerol, 1 mM PMSF, 1% Sarkosyl, 0.1 mg/ml Lysozyme, protease inhibitor cocktail and sonicated on ice. DNase I was added after sonication and the lysates were further incubated for ~60 min before centrifugation at 21,000 × *g* for 40 min. The supernatants were subjected to SDS-PAGE to confirm expression by Coomassie staining and subsequently used for pull-down assays. MBP-tagged Orai1 constructs were expressed in Lemo21 (DE3) *E. coli* cells, induced with 0.3 mM IPTG (Isopropyl β- d-1-thiogalactopyranoside) for 14–18 hr at 18 °C. The cell pellets were lysed in buffer containing 50 mM Tris-Cl (pH 8.0), 150 mM NaCl, 5% Glycerol, 1 mM PMSF, 0.1 mg/ml Lysozyme and protease inhibitor cocktail (1:200) and sonicated on ice. DNase I was added after sonication and the lysates were further incubated for ~60 min before centrifugation at 21,000 × *g* for 40 min. The supernatants were collected and subjected to SDS-PAGE to confirm expression by Coomassie staining and used for pull-down assays. Following lysis, the His_6_-tagged STX11 proteins were purified using Talon Beads and MBP-tagged Orai1 proteins using Dextrin Sepharose/Amylose resin according to the manufacturer’s guidelines. For in vitro binding assays, lysates prepared from cells expressing MBP or MBP-tagged Orai1 fragments were incubated with 25 μl Dextrin Sepharose Beads and incubated for 2 hr at 4 °C. After incubation, beads were washed thrice and ~50–125 ng of purified His-tagged full-length STX11, His-tagged SNARE or His-tagged H_abc_ domains were diluted in buffer containing 50 mM Tris-Cl (pH 8), 150 mM NaCl, 5% Glycerol, and 0.1% NP-40 and added to the beads. Following 1 hr of incubation at 4 °C, the beads were washed thrice, re-suspended and boiled in the binding buffer containing 1 X Laemmli and 120 mM DTT. The eluate was subjected to SDS-PAGE and western blot. MBP and MBP-tagged proteins were detected with Ponceau staining, Full-length STX11 using Rabbit anti-STX11 primary antibody (Thermo) followed by Donkey anti-rabbit HRP and His-tagged STX11 fragments were detected using mouse anti-6XHis primary antibody (Invitrogen) followed by Donkey anti-mouse HRP secondary antibody. Densitometric quantification of the relative band intensities was performed using ImageJ software and plotted in Origin software. To control for variation in experimental repeats, the band intensity of the control complex for wild-type (MBP-Orai1-WT:His-STX11-WT) for individual repeats was set to 100% and relative intensities of bands from other test groups were calculated with respect to this.

### BS3 crosslinking of Flag-Orai1 oligomers

Scramble and STX11 shRNA transduced HEK293 cells were transfected with Flag-Orai1, harvested, washed, pelleted and subjected to one cycle of snap freeze-thaw. The pellet was resuspended in ice-cold lysis buffer (1 X HBSS, 1 mM PMSF and protease inhibitor cocktail), sonicated for 30 s (Branson 2510) and mechanically homogenized using dounce homogenizer. The homogenized supernatant was spun at 1500 × *g* for 10 mins at 4 °C, to pellet the cell debris. The supernatant was ultracentrifuged at 105,000 × *g* for 1 hr at 4 °C (Optima MAX-XP ultracentrifuge, Beckman Coulter). The membrane fraction pellet was resuspended in lysis buffer and re-homogenized using dounce homogenizer. The protein concentration was estimated using BCA assay, and BS3 crosslinker was added to the resuspended membranes at a final concentration of 1 mM and incubated for 30 min at RT. BS3 was quenched using 50 mM Tris-HCl pH 7.5 for 15 min at RT, followed by the addition of 1 X Laemmli, 100 mM DTT, and incubation at 37 °C for 10 min. The samples were resolved using 4–15% gradient SDS-PAGE and subjected to western blot using anti-Flag antibody.

### Quantification of Flag-Orai1 oligomers using western blot

Equal size ROIs were drawn over corresponding protein bands of the same size using ImageJ. The total intensity of each band was measured by calculating the area under the intensity curve, and its relative proportion was calculated by dividing the raw intensity of each band by the sum of intensities of all the bands in the respective lanes.

### qPCR to estimate gene expression in Jurkat T cells

Scr and STX11 shRNA-treated Jurkat T cells were rested for ~1 hr prior to the assay, stimulated with soluble anti-CD3 (5–10 μg/ml) for 3 hr, and washed with HBSS. Total RNA was isolated using RNeasy Mini kit as per manufacturer’s instructions. cDNA was synthesized using random hexamers and Invitrogen Superscript IV kit and quantified using Qubit ssDNA assay. Taqman probes for IL-2 and beta-actin housekeeping control were used for performing the qPCR in triplicates. Data analysis was done by calculating the double delta C_t_ values.

### EIA to measure IL-2 levels in supernatants of Jurkat T cells

Scr and STX11 shRNA-treated Jurkat T cells were stimulated with plate-coated anti-CD3 (10 μg/ml), and the culture supernatants were collected at 12-and 24 hr post-stimulation. EIA plates were coated with purified Mouse anti-human IL-2 capture antibody (1 μg/ml) overnight at 4 °C, washed with 0.05% PBST, and blocked with 2% BSA for 2 hr. After blocking, the plates were incubated with the culture supernatants for 2 hr at RT, washed, and incubated with the detection antibody (Biotin Mouse anti-human IL-2, 1 μg/ml) for 2 hr. After washing, Streptavidin HRP (0.1 μg/ml) followed by TMB reagent were added. 250 mM HCl was added to stop the reaction, and absorbance was measured at 450 nm.

### Isolation and culture of human PBMCs

Whole blood freshly collected in Heparin or Citrate Phosphate Dextrose Adenine (CPDA) solution was subjected to density gradient centrifugation using Ficoll-Paque PLUS media. The buffy coat was separated, washed twice with HBSS, and cultured with IL-2 (50 ng/ml). Cells were stimulated once per week with PHA (2 μg/ml) for 72 hr and rested in IL-2 for the remaining 72–96 hr. Unless specified, all the assays were performed following 24–48 hr of rest post-stimulation.

### Isolation of genomic DNA and sequencing

Genomic DNA was extracted from PBMCs using Phenol-Chloroform-Isoamyl alcohol. STX11 genomic region flanking the mutation was PCR amplified using primers: 5’ Forward -CATGCACGACTACAACCAGGC and 3’ Reverse -GGGACAGCAGAAGCAGCAGAGGG. The resulting PCR products were separated on 2% agarose gel, excised, and extracted using the Macherey-Nagel Nucleospin columns and subjected to Sanger sequencing using the 5’ Forward PCR primer to confirm the mutation.

### Measurement of SOCE in human PBMCs

PBMCs in culture were washed, rested, and allowed to adhere to freshly carbon-coated glass bottom dishes for 1 hr in plain RPMI at 37 °C. The cells were washed with HBSS and incubated with 1 ml of 1 μM Fura-2-AM diluted in Ringer’s buffer for 40 min, washed, and incubated for an additional 10 min to allow de-esterification of the dye. The assay was started with 1 ml of Calcium-free Ringer’s buffer in the imaging dish, and images were captured every 4 s. After capturing the baseline for ~60 s, stores were depleted by the addition of 1 μM TG. In other assays, 10 μg/ml of anti-CD3 and 5 μg/ml of the secondary antibody were used to cross-link the TCRs and thereby induce store depletion. ~5 min post-store-depletion, Calcium Chloride (CaCl_2_) was added back to the dish at a final concentration of 2 mM to estimate the magnitude of SOCE.

### Degranulation assay

PBMCs were cultured in RPMI containing 10% FBS and 50 ng/ml IL-2 and stimulated with PHA (2 μg /ml) 48 hr before the assay. 24 hr prior to the assay, IL-2 was washed off, but PHA was re-added. On the day of the assay, cells were washed twice to remove PHA and any growth factors. To measure degranulation, cells were either left unstimulated, stimulated with a combination of anti-CD3 (10 μg/ml plate-coated), anti-CD28 (2 μg/ml soluble), and anti-CD49d (2 μg/ml soluble), or a combination of Ionomycin (1 μM) and PMA (50 ng/ml) for 3.5 hr. CD107a-PE antibody (1:50 dilution) was added to each of the three groups at the start of stimulation. Following stimulations, cells were transferred to ice, washed with cold HBSS containing 2% FBS, and incubated with anti-CD8 APC for 20 min, washed twice with cold HBSS, and analyzed using Cytoflex (Beckman Coulter) flow cytometer.

### qPCR to estimate gene expression in human PBMCs

PBMCs were taken off IL-2 48 hr prior to the assay. On the day of the assay, cells were washed twice, left unstimulated, and either stimulated with anti-CD3 (10 μg/ml, plate-coated), anti-CD28 (2 μg/ml), and anti-CD49d (2 μg/ml) or with Ionomycin (1 μM) and PMA (50 ng/ml) for 6 hr. To end the stimulation, cells were washed with cold HBSS, pelleted, and used for RNA extraction. Total RNA was isolated using Qiagen RNeasy mini kit, and cDNA was synthesized using random hexamers and Superscript IV (Invitrogen). cDNA was quantified using Qubit and subjected to qPCR analysis using Taqman probes for IL-2 and beta-actin housekeeping gene control in triplicates. Analysis was performed by calculating the double delta C_t_ values.

### Transduction of human PBMCs with pMSCV-STX11

For viral transduction of PBMCs, polystyrene non-TC treated 24-well plates were coated with retronectin (20 μg/ml) overnight at 4 °C, blocked with 2% BSA for 30 min at RT, and washed twice with HBSS. The retroviral supernatants were added onto the coated wells, and the plates were spun at 1800 × *g* for 2 hr at 30 °C. Following spin, the wells were washed with the blocking solution. PBMCs cultured in RPMI containing 10% FBS were stimulated with PHA (2 μg/ml) and IL-2 (50 ng/ml) for 48 hr prior to transduction. Stimulated PBMCs were transferred to coated plates at a density of 0.25 million cells/well, centrifuged at 400 × *g* for 40 min at 30 °C. The cells were analyzed 48–72 hr post transduction.

### Patch clamp measurements

Patch clamp recordings were performed by using an Axopatch 200B amplifier (Axon Instruments, Foster City, CA) interfaced to an ITC-18 input/output board (Instrutech, Port Washington, NY) and an iMac G5 computer. Currents were filtered at 1 kHz with a four-pole Bessel filter and sampled at 5 kHz. Recording electrodes were pulled from 100 µl pipettes (VWR), coated with Sylgard, and fire-polished to a final resistance of 2–5 MΩ. Stimulation and data acquisition and analysis were performed by using in-house routines developed by RS Lewis (Stanford University) on the Igor Pro platform (Wavemetrics, Lake Oswego, OR). All data were corrected for the liquid junction potential of the pipette solution relative to Ringer’s in the bath (–10 mV) and for leak currents collected in 20 mM extracellular free calcium plus 10 µM La^3+^. The holding potential was +30 mV. The standard voltage stimulus consisted of a 100 ms step to –100 mV followed by a 100 ms ramp from –100 to +100 mV applied at 1 s intervals. I_CRAC_ was typically activated by passive depletion of ER Ca^2+^ stores by intracellular dialysis of 8 mM BAPTA.

### Solutions and chemicals for patch clamp

The standard extracellular Ringer solution contained 130 mM NaCl, 4.5 mM KCl, 20 mM CaCl_2_, 10 mM tetraethylammonium chloride (TEA-Cl), 10 mM D-glucose, and 5 mM HEPES (pH 7.4 with NaOH). The standard divalent-free (DVF) Ringer solutions contained (in mM) 150 NaCl, 10 HEDTA, 1 EDTA, and 10 HEPES (pH 7.4). The internal solution contained: 135 mM Cs aspartate, 8 mM MgCl_2_, 8 mM Cs-BAPTA, and 10 mM HEPES (pH 7.2 with CsOH).

### Patch clamp data analysis

Analysis of current amplitudes was typically performed by measuring the peak currents during the –100 mV pulse. Averaged results are presented as the mean value ± SEM. Reversal potentials were measured from the average of several leak-subtracted sweeps (4–6) in each cell. For datasets with two groups, statistical analysis was performed with a two-tailed t test to compare between control and test conditions with a confidence level of 95%, and results with p<0.05 were considered statistically significant.

### STX11:Orai1 and SOAR:Orai complex prediction

Full-length STX11 and its two domains, (H_abc_ 41–167 and SNARE 183–267) and Orai1 (N-terminus 1–87 and C-terminus 256–301) were used to generate the complex of STX11 and Orai1 using AlphaFold3 (AF3) in all combinations. For Stim1 SOAR, residues 344–444 were used along with Orai C-terminus 256–301. The resultant models were assessed using ipTM and pTM scores. The best scoring combinations were further considered to generate more models by changing the seed values. The best model in terms of highest ipTM and pTM values was considered for further analysis. A custom script was used to analyze the contact frequency of interacting residues of STX11-H_abc_ and Orai1 C-terminus complex as well as SOAR and Orai1 C-terminus, across all predicted models.

### Molecular dynamics (MD) simulation

The STX11 H_abc_:Orai1 C-terminus and SOAR:Orai1 C-terminus complexes were subjected to all-atom MD simulations. Initially, the complexes were prepared using the protein-preparation module of Schrödinger, which involves H-bond network optimization and restrained minimization of the initial structure. The prepared structures were solvated using the TIP3P water model in an orthorhombic water box and neutralized by adding counter ions. The OPLS4 force field was used, and the simulation system was generated by specifying 150 mM salt (NaCl). A buffer distance of 10 Å beyond the solute in each direction was used. The solvated system was subjected to the default relaxation protocol of Desmond followed by a production run for 500 ns at 300 K and 1 atm pressure in the NPT ensemble. The default relaxation protocol includes several short simulation steps. (1) Brownian dynamics simulation for 100 ps at 10 K temperature in NVT with restraints on solute heavy atoms (2) Simulation in NVT ensemble at 10 K for 12 ps with restraints on solute heavy atoms (3) 12 ps simulation in NPT ensemble at 10 K with restraints on solute heavy atoms (4) Simulation in NPT ensemble for 12 ps with restraints on solute heavy atoms (5) Simulation in NPT ensemble for 24 ps without restraints. Three independent runs were executed with different initial seeds for both structures.

### Analysis of MD simulation

The MD runs were analyzed for the stability of the complex and interactions between the subunits. Simulation stability was assessed based on the protein backbone RMSD over simulation time. The RMSD was calculated using the simulation interaction diagram (SID) module of Schrödinger. The first frame was used as the reference frame for RMSD calculation. The script ‘analyze_trajectory_ppi.py’ was used to calculate the interactions between STX11:Orai1 and Stim1:Orai1. The binding energy (ΔG) at each nanosecond across simulation was calculated using the ‘thermal_mmgbsa.py’ script of Schrödinger. The representative structure was generated through trajectory clustering using the trajectory clustering tool in Schrödinger, which applies affinity propagation to the pairwise backbone RMSD-based similarity matrix. Within each affinity propagation run, convergence was defined as no change in the set of exemplar frames for 15 consecutive iterations, with a maximum of 400 iterations per run. If convergence was not reached, the damping factor was increased from 0.5 in increments of 0.01 until convergence. The last 200 ns frames from each run were combined and clustered using the scripts ‘trj_merge.py’ and ‘trajectory cluster’ of Schrödinger. The cluster representative of the largest cluster was used to analyze the protein-protein interactions. PDB Sum was used to calculate the interactions in the cluster representative. The respective PDB files have been deposited as Supplementary files 3 and 4. Figures were generated using Pymol and MD simulation videos using Maestro.

### Statistical analysis

Statistical analysis was done using unpaired two-tailed Student’s *t* test where only two groups were being compared and ANOVA where more than two groups were present. Normality of distribution was checked in Origin. Mann Whitney U Test was used for datasets with non-normal (or skewed) distribution. p Values for all significance tests were *p<0.05; **p<0.01; ***p<0.001.

## Data Availability

All data generated or analyzed during this study are included in the manuscript and supporting files; source data files have been provided for all figures. The custom script used for analysis of MD simulation and resulting videos has been included as source data or rich media files. PDB files have been included as Supplementary files 3 and 4.

## Additional files

Supplementary file 1. List of primers and shRNAs used in this paper.

Supplementary file 2. List of plasmids, including the newly generated constructs used in this paper.

Supplementary file 3. Folder containing the PDB file for Figure 5L.

Supplementary file 4. Folder containing the PDB file for Figure 5—figure supplement 6B.

MDAR checklist

## Appendix 1

**Appendix 1—key resources table:**

Reagent type (species) or resource	Designation	Source or reference	Identifiers	Additional information
Antibody	Mouse monoclonal anti-myc (9E10)	Abcam	ab32 (RRID:AB_303599)	WB ( 1:1000)
Antibody	Rabbit polyclonal anti-SNAP23	SYSY	111203	WB (1:1500)
Antibody	Mouse monoclonal anti-myc (9E10)	Culture supernatant, in house (this paper)	-	WB (0.25 μg/ml)
Antibody	Mouse monoclonal anti-SNAP25	SYSY	111011	WB (1:1500)
Antibody	Rabbit polyclonal anti-SNAP29	SYSY	111303	WB (1:1500)
Antibody	Rabbit polyclonal anti-GFP	Molecular Probes, Invitrogen	A11122 (RRID:AB_221569)	WB (1:2500)
Antibody	Rabbit polyclonal anti-flag	Sigma-Aldrich	F7425 (RRID:AB_439687)	Co-IP (1:1600)
Antibody	Mouse monoclonal anti-flag (M2)	Sigma-Aldrich	F3165 (RRID:AB_259529)	WB (1:1500)
Antibody	Mouse monoclonal anti-6X His	Thermo Fisher Scientific	MA1-135 (RRID:AB_2536841)	WB (1:2000)
Antibody	Rabbit polyclonal anti-STX11	Sigma-Aldrich	SAB4301593	IF, WB (1:1000)
Antibody	Rabbit polyclonal anti-STX11	SYSY	110113	IF (1.67 μg/ml)
Antibody	Rabbit polyclonal anti-STX11	Thermo Fisher Scientific	PA5-50800 (RRID:AB_2636252)	WB (1:2000)
Antibody	Rabbit polyclonal anti-STX11	Proteintech	13301–1-AP (RRID:AB_2198214)	IF (0.3 μg/ml)
Antibody	Mouse monoclonal anti-NFATc2 (4G6-G5)	Santa Cruz Biotechnology	sc-7296 (RRID:AB_628012)	WB (1:500)
Antibody	Rabbit polyclonal anti-NFAT1	Cell Signaling Technology	4389 (RRID:AB_1950418)	IF (1:200)
Antibody	Mouse monoclonal anti-Clathrin	BD Biosciences	610499 (RRID:AB_397865)	WB (1:3000)
Antibody	Goat polyclonal anti-Lamin B (M-20)	Santa Cruz Biotechnology	sc-6217 (RRID:AB_648158)	WB (1:500)
Antibody	Rabbit monoclonal mAb HA-tag	Cell Signaling Technology	3724 S (RRID:AB_1549585)	IF (1:1000)
Antibody	Alpaca polyclonal VHH anti-rabbit AF488	Jackson	611-544-152	IF (1.375 μg/ml)
Antibody	A647 conjugated Goat polyclonal anti-rabbit	Molecular Probes	A-21245 (RRID:AB_2535813)	IF (1:800)
Other	DAPI	Thermo Fisher Scientific	D1306 (RRID:AB_2629482)	Nuclear staining in Figures 2 and 4 (2 μg/ml)
Antibody	Donkey polyclonal anti-rabbit A647	Jackson	711-605-152	IF (1:800)
Antibody	HRP conjugated Mouse monoclonal anti-GAPDH	Sigma-Aldrich	G9295 (RRID:AB_1078992)	WB (1:40,000)
Antibody	HRP conjugated Donkey Polyclonal anti-Rabbit	Jackson	711-035-152	WB (1:50,000)
Antibody	HRP conjugated Donkey polyclonal anti-Mouse	Jackson	715-035-151	WB (1:50,000)
Antibody	Alexa fluor 647 conjugated Donkey polyclonal anti-mouse	Jackson	715-705-150	IF (1:500)
Other	Alexa fluor 647 conjugated Alpha Bungarotoxin	Thermo Fisher Scientific	B35450	Labelling Orai1-BBS-YFP in Figure 2, Figure 2—figure supplements 1 and 2 (1 μg/ml)
Antibody	HRP conjugated rabbit polyclonal anti-GFP	Thermo Fisher Scientific	A10260	WB (1:1000)
Antibody	HRP conjugated Goat polyclonal anti-rabbit Fc	Jackson	111-035-008	WB (1:50,000)
Other	HRP conjugated Streptavidin	Jackson	016-030-084	EIA in Figure 4 (0.1 μg/ml)
Antibody	Purified Mouse monoclonal anti-human IL-2	BD Biosciences	BD #555051 (RRID:AB_395672)	EIA (1 μg/ml)
Antibody	Biotin conjugated Purified mouse monoclonal anti-human IL-2	BD Biosciences	555040 (RRID:AB_395666)	EIA (1 μg/ml)
Antibody	Ultra-LEAF Purified mouse monoclonal anti-human CD3	BioLegend	317326 (RRID:AB_11150592)	Stimulation for Degranulation assay (5–10 μg/ml)
Antibody	Ultra-LEAF Purified mouse monoclonal anti-human CD28	BioLegend	302934 (RRID:AB_11148949)	Stimulation for Degranulation assay (2 μg/ml)
Antibody	Ultra-LEAF Purified mouse monoclonal anti-human CD49d	BioLegend	304340 (RRID:AB_2810444)	Stimulation for Degranulation assay (2 μg/ml)
Antibody	PE mouse monoclonal anti-human CD107a (LAMP-1)	BioLegend	328608 (RRID:AB_1186040)	FACS (1:50)
Antibody	APC mouse monoclonal anti-human CD8 antibody	BioLegend	344722 (RRID:AB_2075388)	FACS (0.5 μg/ml)
Antibody	FluoTag-X4 conjugated camelid monoclonal Anti-Rabbit IgG sdAb	SYSY	N2404-At488-S	IF (1:400)
Antibody	Mouse monoclonal Anti-rat GM130	BD Biosciences	610823 (RRID:AB_398142)	IF (0.5 μg/ml)
Antibody	Mouse monoclonal Anti-rat KDEL	Santa Cruz Biotechnology	sc-58774 (RRID:AB_784161)	IF (1:100)
Other	Fura-2-AM	Thermo Fisher Scientific	F1221	Single cell calcium imaging in Figures 1, 6—8 (1 μM)
Other	Thapsigargin (TG)	Sigma-Aldrich	T9033-1MG	Store depletion in Figures 1, 2, 4—8 (1 μM)
Other	Polybrene (Hexadimethrine-bromide)	Sigma-Aldrich	107689–10 G	Viral transduction in Figures 1—8 (8 μg/ml)
Other	Alexa Fluor 647 ChromPure Human Transferrin	Jackson	009-600-050	Transferrin recycling assay in Figure 2 (20 μg/ml)
Other	Disodium 4-Nitrophenyl Phosphate Hexahydrate	TCI	N0241	Substrate for AP assay in Figure 2 (1 mg/ml)
Other	Dextran-FITC	TCI	F0918	Dextran-FITC uptake assay in Figure 2 (1 mg/ml)
Other	NE-PER kit	Thermo Fisher Scientific	78833	Nuclear and cytosolic extracts in Figure 4
Other	Protein A/G Mag Sepharose xtra	Cytiva	28967056/66	Co-IPs in Figure 5, Figure 2—figure supplement 3 and Figure 5—figure supplement 4
Other	Protein A/G 4 Fast Flow Sepharose	Cytiva	17528001	Co-IPs in Figure 5—figure supplement 4, Figure 2—figure supplement 3
Other	Mammalian Protease Inhibitor Cocktail	Sigma-Aldrich	539134	Cell lysis buffer in Figure 5, Figure 2—figure supplement 3 and Figure 5—figure supplement 4, Figures 1, 6 and 8 (1:200)
Other	Protease Inhibitor Cocktail (Bacterial)	Calbiochem	539132	In vitro binding assay in Figures 5 and 6 and associated figure supplements (1:200)
Other	Talon Beads	Takara	635502	In vitro binding assay in Figures 5 and 6 and associated figure supplements
Other	Dextrin Sepharose Beads	Cytiva	28935597	In vitro binding assay in Figures 5 and 6 and associated figure supplements
Other	HEPES	Sigma-Aldrich	H3375-100G	Ringer’s buffer, and HEK293FT culture media
Other	Lipofectamine 2000	Thermo Fisher Scientific	11668019	Transfection reagent in Figures 1, 2, 5—8
Other	Lipofectamine 3000	Thermo Fisher Scientific	L3000001	Transfection reagent in Figures 1, 2, 5—8
Strain, strain background (*Escherichia coli*)	Lemo21(DE3)	New England Biolabs	C2528J	Chemically competent cells
Strain, strain background (*Escherichia coli*)	DH5α	New England Biolabs	C2987H	Chemically competent cells
Other	IPTG	Sigma-Aldrich	16758–10 G	Induction of His-STX11 expression in Figures 5 and 6 and associated figure supplements
Other	Sarkosyl (N-lauroylsarcosine sodium salt)	Sigma-Aldrich	L9150-50G	Bacterial cell lysis in Figures 5 and 6 and associated figure supplements
Other	Lysozyme	Sigma-Aldrich	62970–5 G-F	Bacterial cell lysis in Figures 5 and 6 and associated figure supplements
Other	DNase I Recombinant RNase-free	Roche	04716728001	Bacterial cell lysis in Figures 5 and 6 and associated figure supplements
Other	PMA (Phorbol Myristate Acetate)	Sigma-Aldrich	P8139	Cell stimulation in Figure 4 (20–50 ng/ml)
Other	Ionomycin	Sigma-Aldrich	407952	Cell stimulation in Figures 2 and 4 (1 μM)
Other	Human IL-2 protein	Acro Biosystems	H5215	Primary T cell culture in Figures 1 and 4 (50 ng/ml)
Other	Lectin from *Phaseolus vulgaris* (PHA)	Sigma-Aldrich	L8754	Primary T cell culture in Figures 1 and 4 (2 μg/ml)
Other	Retronectin	Takara	T100B	Primary T cell transduction in Figure 1 (20 μg/ml)
Other	RNeasy Plus Mini kit	Qiagen	74134	RNA isolation for all qPCR assays in Figure 4 and figure supplements associated with Figures 1 and 2
Other	QIAshredder	Qiagen	79654	Cell disintegration before RNA isolation in Figure 4 and figure supplements associated with Figures 1 and 2
Other	Superscript IV	Thermo Fisher Scientific	18091300	cDNA synthesis for qPCR assays in Figure 4 and figure supplements associated with Figures 1 and 2
Other	Qubit ssDNA Assay kit	Thermo Fisher Scientific	Q10212	cDNA quantification for qPCRs in Figure 4 and figure supplements associated with Figures 1 and 2
Other	Human STX11 TaqMan probe (FAM-MGB)	Thermo Fisher Scientific	Hs00186823_m1	qPCR in figure supplements associated with Figures 1 and 2
Other	Human Beta Actin TaqMan probe (FAM-MGB)	Thermo Fisher Scientific	Hs01060665_g1	qPCR in Figure 4 and figure supplements associated with Figure 1
Other	Human IL2 Taqman probe (FAM-MGB)	Thermo Fisher Scientific	Hs00174114_m1	qPCR in Figure 4
Other	Human RPL30 Taqman probe (FAM-MGB)	Thermo Fisher Scientific	Hs00265497_m1	qPCR in figure supplements associated with Figures 1 and 2
Other	Paraformaldehyde	Electron Microscopy Sciences	157–8	Cell fixation for IF in Figures 2, 4—7
Other	Bovine Serum Albumin	Jackson	001000173	Blocking agent for IF in Figures 2, 4—7
Other	Luria Bertini Broth	Himedia	M1245	Bacterial cell culture in Figures 5 and 6
Other	HBSS	Sigma-Aldrich	H8264	Cell washing and incubation in Figures 1, 2, 4—8
Other	Terrific Broth	Sigma-Aldrich	T9179	Lemo21 cell culture in Figures 5 and 6
Other	Ampicillin	Sigma-Aldrich	A0166-5G	Antibiotic for bacterial selection (100 μg/ml)
Other	Kanamycin	Sigma-Aldrich	K1876-1G	Antibiotic for bacterial selection (50 μg/ml)
Other	Chloramphenicol	Sigma-Aldrich	C0378-5G	Antibiotic for bacterial selection (100 μg/ml)
Other	30% Acrylamide:Bis Solution (29:1)	Biorad	1610156	SDS-PAGE gels in Figures 1, 4—6 and 8 and their associated figure supplements
Other	DTT (Dithiothreitol)	Biorad	1610611	1 X Laemmli buffer for SDS-PAGE in Figures 1, 4—6 and 8 and associated figure supplements
Other	0.05% Trypsin-EDTA	Gibco	25300054	Trypsinization of HEK293, and HEK293FT cells
Other	0.25% Trypsin-EDTA	Gibco	25200072	Trypsinization of U2OS cells
Other	100 X Penicillin Streptomycin	Sigma-Aldrich	P4333-100ML	Supplement in cell culture medium in Figures 1—8
Other	Puromycin	Gibco	A11138-03	Antibiotic for selection of shRNA treated cells in Figures 1—4, 6—8 and their associated figure supplements
Cell line (*Homo sapiens*)	HEK293	ATCC	CRL-1573 (RRID:CVCL_0045)	Figures 1, 2, 5—8 and associated figure supplements
Cell line (*Homo sapiens*)	Jurkat T cell line [Clone E6-1]	ATCC	TIB-152 (RRID:CVCL_0367)	Figures 1—4 and 6 and associated figure supplements
Cell line (*Homo sapiens*)	U2OS cell line	ATCC	HTB-96 (RRID:CVCL_0042)	Figure 2—figure supplement 2
Cell line (*Homo sapiens*)	HEK293FT	Thermo Fisher Scientific	R70007	Virus generation in Figures 1—8
Other	Universal Mycoplasma Detection Kit	ATCC	30–1012 K	Mycoplasma testing of cell lines
Biological sample (*Homo sapiens*)	Human PBMCs	Dr. Shahrooei Lab	-	Figures 1 and 4
Software, algorithm	Schrödinger Suite (Protein Preparation Wizard, Desmond, Simulation Interaction Diagram, thermal_mmgbsa.py, trj_merge.py, trajectory clustering, analyze_trajectory_ppi.py)	Schrödinger, LLC	-	System preparation, MD simulation, MM/GBSA, and trajectory analysis in Figure 5 and associated figure supplements
Software, algorithm	PyMOL	Schrödinger, LLC	-	Figure generation in Figure 5 and associated figure supplements
Software, algorithm	PDBSum	EMBL-EBI	-	Interaction analysis in Figure 5 and associated figure supplements

## References

[bib1] Augustynek B, Gyimesi G, Dernič J, Sallinger M, Albano G, Klesse GJ, Kandasamy P, Grabmayr H, Frischauf I, Fuster DG, Peinelt C, Hediger MA, Bhardwaj R (2022). Discovery of novel gating checkpoints in the Orai1 calcium channel by systematic analysis of constitutively active mutants of its paralogs and orthologs. Cell Calcium.

[bib2] Barocchi MA, Masignani V, Rappuoli R (2005). Opinion: cell entry machines: a common theme in nature?. Nature Reviews. Microbiology.

[bib3] Bryceson YT, Rudd E, Zheng C, Edner J, Ma D, Wood SM, Bechensteen AG, Boelens JJ, Celkan T, Farah RA, Hultenby K, Winiarski J, Roche PA, Nordenskjöld M, Henter J-I, Long EO, Ljunggren H-G (2007). Defective cytotoxic lymphocyte degranulation in syntaxin-11 deficient familial hemophagocytic lymphohistiocytosis 4 (FHL4) patients. Blood.

[bib4] Chang CL, Chen YJ, Liou J (2017). ER-plasma membrane junctions: Why and how do we study them?. Biochimica et Biophysica Acta. Molecular Cell Research.

[bib5] Chinn IK, Eckstein OS, Peckham-Gregory EC, Goldberg BR, Forbes LR, Nicholas SK, Mace EM, Vogel TP, Abhyankar HA, Diaz MI, Heslop HE, Krance RA, Martinez CA, Nguyen TC, Bashir DA, Goldman JR, Stray-Pedersen A, Pedroza LA, Poli MC, Aldave-Becerra JC, McGhee SA, Al-Herz W, Chamdin A, Coban-Akdemir ZH, Jhangiani SN, Muzny DM, Cao TN, Hong DN, Gibbs RA, Lupski JR, Orange JS, McClain KL, Allen CE (2018). Genetic and mechanistic diversity in pediatric hemophagocytic lymphohistiocytosis. Blood.

[bib6] Clapham DE (2007). Calcium signaling. Cell.

[bib7] Cormet-Boyaka E, Di A, Chang SY, Naren AP, Tousson A, Nelson DJ, Kirk KL (2002). CFTR chloride channels are regulated by a SNAP-23/syntaxin 1A complex. PNAS.

[bib8] Delevoye C, Nilges M, Dautry-Varsat A, Subtil A (2004). Conservation of the biochemical properties of IncA from *Chlamydia trachomatis* and *Chlamydia caviae*: oligomerization of IncA mediates interaction between facing membranes. The Journal of Biological Chemistry.

[bib9] D’Orlando O, Zhao F, Kasper B, Orinska Z, Müller J, Hermans-Borgmeyer I, Griffiths GM, Zur Stadt U, Bulfone-Paus S (2013). Syntaxin 11 is required for NK and CD8. European Journal of Immunology.

[bib10] Dulubova I, Khvotchev M, Liu S, Huryeva I, Südhof TC, Rizo J (2007). Munc18-1 binds directly to the neuronal SNARE complex. PNAS.

[bib11] Feske S, Gwack Y, Prakriya M, Srikanth S, Puppel S-H, Tanasa B, Hogan PG, Lewis RS, Daly M, Rao A (2006). A mutation in Orai1 causes immune deficiency by abrogating CRAC channel function. Nature.

[bib12] Hackmann Y, Graham SC, Ehl S, Höning S, Lehmberg K, Aricò M, Owen DJ, Griffiths GM (2013). Syntaxin binding mechanism and disease-causing mutations in Munc18-2. PNAS.

[bib13] Hellewell AL, Foresti O, Gover N, Porter MY, Hewitt EW (2014). Analysis of familial hemophagocytic lymphohistiocytosis type 4 (FHL-4) mutant proteins reveals that S-acylation is required for the function of syntaxin 11 in natural killer cells. PLOS ONE.

[bib14] Hou X, Burstein SR, Long SB (2018). Structures reveal opening of the store-operated calcium channel Orai. eLife.

[bib15] Jahn R, Scheller RH (2006). SNAREs--engines for membrane fusion. Nature Reviews. Molecular Cell Biology.

[bib16] Jarvis SE, Zamponi GW (2005). Masters or slaves? Vesicle release machinery and the regulation of presynaptic calcium channels. Cell Calcium.

[bib17] Jessen B, Kögl T, Sepulveda FE, de Saint Basile G, Aichele P, Ehl S (2013). Graded defects in cytotoxicity determine severity of hemophagocytic lymphohistiocytosis in humans and mice. Frontiers in Immunology.

[bib18] Lang T, Bruns D, Wenzel D, Riedel D, Holroyd P, Thiele C, Jahn R (2001). SNAREs are concentrated in cholesterol-dependent clusters that define docking and fusion sites for exocytosis. The EMBO Journal.

[bib19] Lee S, Shin J, Jung Y, Son H, Shin J, Jeong C, Kweon D-H, Shin Y-K (2020). Munc18-1 induces conformational changes of syntaxin-1 in multiple intermediates for SNARE assembly. Scientific Reports.

[bib20] Li Z, Liu L, Deng Y, Ji W, Du W, Xu P, Chen L, Xu T (2011). Graded activation of CRAC channel by binding of different numbers of STIM1 to Orai1 subunits. Cell Research.

[bib21] Li P, Miao Y, Dani A, Vig M (2016). α-SNAP regulates dynamic, on-site assembly and calcium selectivity of Orai1 channels. Molecular Biology of the Cell.

[bib22] Lu X, Wang Y, Yu K, Li M, Yang X, Shen Y (2024). Activated human Orai1 channel in lipid biolayer may exist as a pentamer. Biochemical and Biophysical Research Communications.

[bib23] Macartney CA, Weitzman S, Wood SM, Bansal D, Steele M, Meeths M, Abdelhaleem M, Bryceson YT (2011). Unusual functional manifestations of a novel STX11 frameshift mutation in two infants with familial hemophagocytic lymphohistiocytosis type 4 (FHL4). Pediatric Blood & Cancer.

[bib24] Marsh RA, Satake N, Biroschak J, Jacobs T, Johnson J, Jordan MB, Bleesing JJ, Filipovich AH, Zhang K (2010). STX11 mutations and clinical phenotypes of familial hemophagocytic lymphohistiocytosis in North America. Pediatric Blood & Cancer.

[bib25] Maul-Pavicic A, Chiang SCC, Rensing-Ehl A, Jessen B, Fauriat C, Wood SM, Sjöqvist S, Hufnagel M, Schulze I, Bass T, Schamel WW, Fuchs S, Pircher H, McCarl C-A, Mikoshiba K, Schwarz K, Feske S, Bryceson YT, Ehl S (2011). ORAI1-mediated calcium influx is required for human cytotoxic lymphocyte degranulation and target cell lysis. PNAS.

[bib26] McGraw TE, Maxfield FR (1990). Human transferrin receptor internalization is partially dependent upon an aromatic amino acid on the cytoplasmic domain. Cell Regulation.

[bib27] McNally BA, Somasundaram A, Yamashita M, Prakriya M (2012). Gated regulation of CRAC channel ion selectivity by STIM1. Nature.

[bib28] Mercer JC, Dehaven WI, Smyth JT, Wedel B, Boyles RR, Bird GS, Putney JW (2006). Large store-operated calcium selective currents due to co-expression of Orai1 or Orai2 with the intracellular calcium sensor, Stim1. The Journal of Biological Chemistry.

[bib29] Miao Y, Miner C, Zhang L, Hanson PI, Dani A, Vig M (2013). An essential and NSF independent role for α-SNAP in store-operated calcium entry. eLife.

[bib30] Miao Y, Bhushan J, Dani A, Vig M (2017). Na^+^ influx *via* Orai1 inhibits intracellular ATP-induced mTORC2 signaling to disrupt CD4 T cell gene expression and differentiation. eLife.

[bib31] Müller M-L, Chiang SCC, Meeths M, Tesi B, Entesarian M, Nilsson D, Wood SM, Nordenskjöld M, Henter J-I, Naqvi A, Bryceson YT (2014). An N-Terminal missense mutation in STX11 causative of FHL4 Abrogates Syntaxin-11 Binding to Munc18-2. Frontiers in Immunology.

[bib32] Neveu E, Khalifeh D, Salamin N, Fasshauer D (2020). Prototypic SNARE proteins are encoded in the genomes of heimdallarchaeota, potentially bridging the gap between the prokaryotes and eukaryotes. Current Biology.

[bib33] Park CY, Hoover PJ, Mullins FM, Bachhawat P, Covington ED, Raunser S, Walz T, Garcia KC, Dolmetsch RE, Lewis RS (2009). STIM1 clusters and activates CRAC channels via direct binding of a cytosolic domain to Orai1. Cell.

[bib34] Paumet F, Wesolowski J, Garcia-Diaz A, Delevoye C, Aulner N, Shuman HA, Subtil A, Rothman JE (2009). Intracellular bacteria encode inhibitory SNARE-like proteins. PLOS ONE.

[bib35] Peinelt C, Vig M, Koomoa DL, Beck A, Nadler MJS, Koblan-Huberson M, Lis A, Fleig A, Penner R, Kinet J-P (2006). Amplification of CRAC current by STIM1 and CRACM1 (Orai1). Nature Cell Biology.

[bib36] Prakriya M, Lewis RS (2006). Regulation of CRAC channel activity by recruitment of silent channels to a high open-probability gating mode. The Journal of General Physiology.

[bib37] Prakriya M, Lewis RS (2015). Store-operated calcium channels. Physiological Reviews.

[bib38] Ramanagoudr-Bhojappa R, Miao Y, Vig M (2021). High affinity associations with α-SNAP enable calcium entry via Orai1 channels. PLOS ONE.

[bib39] Soboloff J, Spassova MA, Tang XD, Hewavitharana T, Xu W, Gill DL (2006). Orai1 and STIM reconstitute store-operated calcium channel function. The Journal of Biological Chemistry.

[bib40] Stathopulos PB, Schindl R, Fahrner M, Zheng L, Gasmi-Seabrook GM, Muik M, Romanin C, Ikura M (2013). STIM1/Orai1 coiled-coil interplay in the regulation of store-operated calcium entry. Nature Communications.

[bib41] Toft-Bertelsen TL, Ziomkiewicz I, Houy S, Pinheiro PS, Sørensen JB (2016). Regulation of Ca2+ channels by SNAP-25 via recruitment of syntaxin-1 from plasma membrane clusters. Molecular Biology of the Cell.

[bib42] van den Bogaart G, Meyenberg K, Risselada HJ, Amin H, Willig KI, Hubrich BE, Dier M, Hell SW, Grubmüller H, Diederichsen U, Jahn R (2011). Membrane protein sequestering by ionic protein-lipid interactions. Nature.

[bib43] Vig M, Beck A, Billingsley JM, Lis A, Parvez S, Peinelt C, Koomoa DL, Soboloff J, Gill DL, Fleig A, Kinet J-P, Penner R (2006a). CRACM1 multimers form the ion-selective pore of the CRAC channel. Current Biology.

[bib44] Vig M, Peinelt C, Beck A, Koomoa DL, Rabah D, Koblan-Huberson M, Kraft S, Turner H, Fleig A, Penner R, Kinet J-P (2006b). CRACM1 is a plasma membrane protein essential for store-operated Ca2+ entry. Science.

[bib45] Vig M, Kinet JP (2007). The long and arduous road to CRAC. Cell Calcium.

[bib46] Vig M, DeHaven WI, Bird GS, Billingsley JM, Wang H, Rao PE, Hutchings AB, Jouvin M-H, Putney JW, Kinet J-P (2008). Defective mast cell effector functions in mice lacking the CRACM1 pore subunit of store-operated calcium release-activated calcium channels. Nature Immunology.

[bib47] Vig M, Kinet JP (2009). Calcium signaling in immune cells. Nature Immunology.

[bib48] Yoon TY, Munson M (2018). SNARE complex assembly and disassembly. Current Biology.

[bib49] Zhang K, Astigarraga I, Bryceson Y, Adam MP (1993). GeneReviews.

[bib50] Zhang SL, Yeromin AV, Zhang XH-F, Yu Y, Safrina O, Penna A, Roos J, Stauderman KA, Cahalan MD (2006). Genome-wide RNAi screen of Ca(2+) influx identifies genes that regulate Ca(2+) release-activated Ca(2+) channel activity. PNAS.

[bib51] Zhang Y, Wang Y, Liu J, Bei W, Wang H, Wang J, Chen L, Wang Y (2026). Structural dynamics of the human Orai1 channel revealed by cryo-electron microscopy. PLOS ONE.

[bib52] Zhou Y, Cai X, Loktionova NA, Wang X, Nwokonko RM, Wang X, Wang Y, Rothberg BS, Trebak M, Gill DL (2016). The STIM1-binding site nexus remotely controls Orai1 channel gating. Nature Communications.

