# Supplementary material for: Syntaxin11 deficiency inhibits CRAC channel priming to suppress cytotoxicity and gene expression in T lymphocytes independent of membrane trafficking
Source: eLife. 2026 Oct 19;15:RP111335. doi: 10.7554/eLife.111335 (PMC13679927; doi:10.7554/eLife.111335)
Supplement: Supplementary file 3 [file elife-111335-fig1-data3.zip › Figure 1-source data 3/Figure 1G.pdf]

Figure 1G

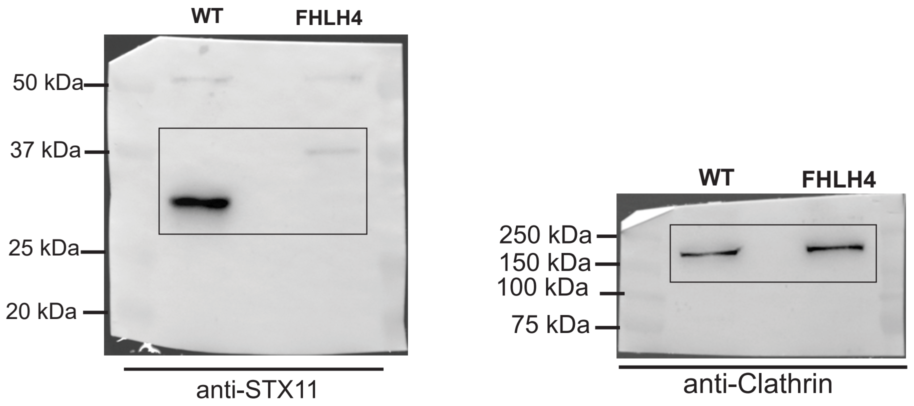
WT
FHLH4
50 kDa
37 kDa
25 kDa
20 kDa
anti-STX11
WT
FHLH4
250 kDa
150 kDa
100 kDa
75 kDa
anti-Clathrin
