# Supplementary material for: Syntaxin11 deficiency inhibits CRAC channel priming to suppress cytotoxicity and gene expression in T lymphocytes independent of membrane trafficking
Source: eLife. 2026 Oct 19;15:RP111335. doi: 10.7554/eLife.111335 (PMC13679927; doi:10.7554/eLife.111335)

HEK293 untransfected cells_BTXA647 background binding

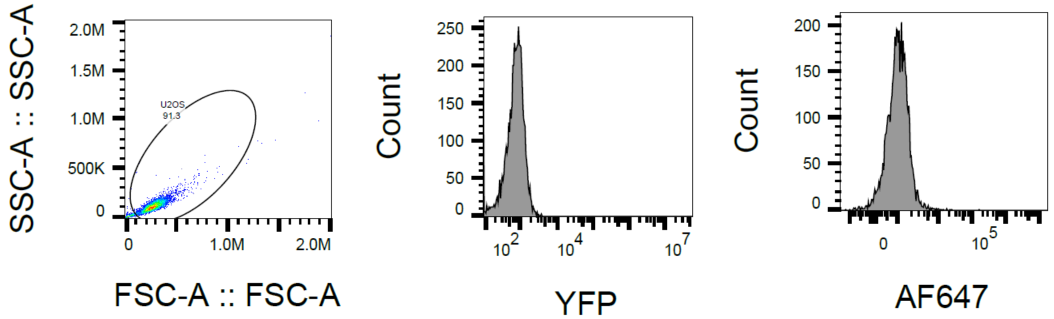

Scr shRNA_Resting (black solid line)

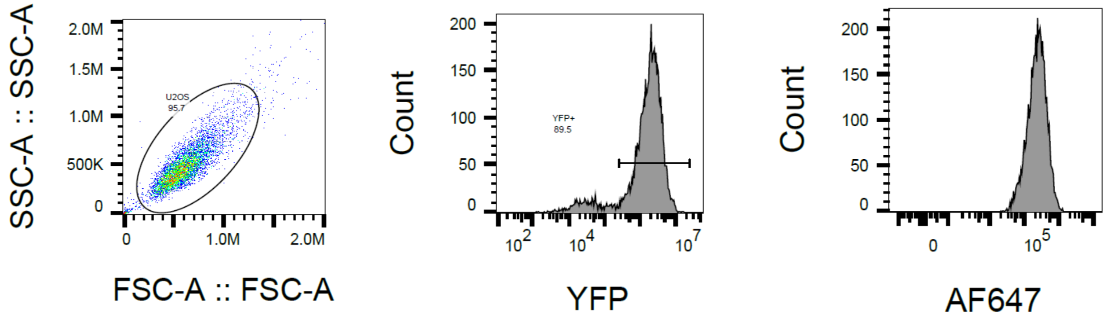

Scr shRNA_Store-depleted (black broken line)

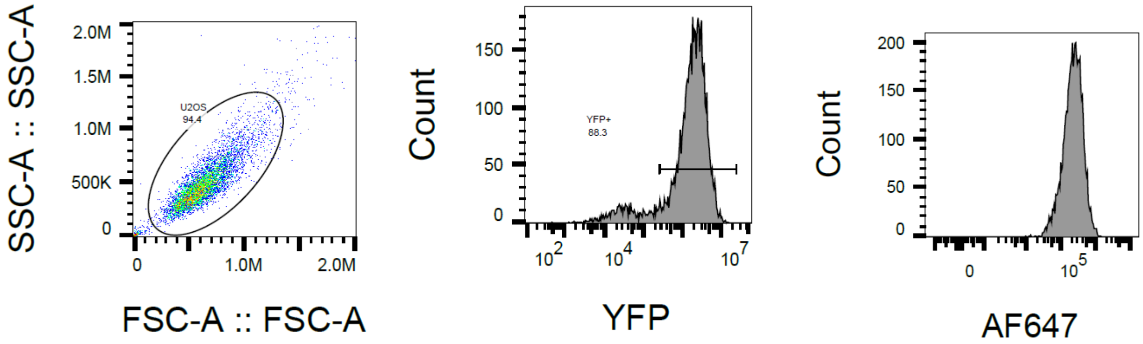

STX11 shRNA_Resting (red solid line)

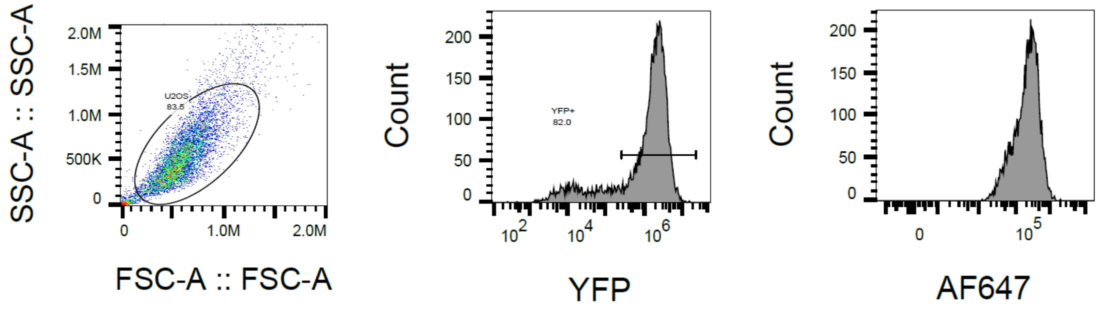

STX11 shRNA_Store-depleted (red broken line)

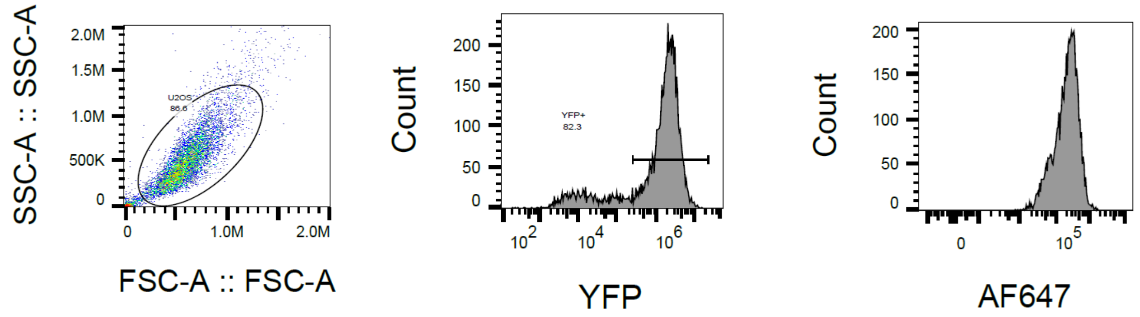

Supplement: Supplementary file 6 [file elife-111335-fig2-figsupp2-data1.zip › Gating Strategy.pdf]
