# Supplementary material for: Syntaxin11 deficiency inhibits CRAC channel priming to suppress cytotoxicity and gene expression in T lymphocytes independent of membrane trafficking
Source: eLife. 2026 Oct 19;15:RP111335. doi: 10.7554/eLife.111335 (PMC13679927; doi:10.7554/eLife.111335)
Supplement: Supplementary file 9 [file elife-111335-fig4-data1.zip › Figure 4-source data 1/Figure 4A.pdf]

Figure 4A

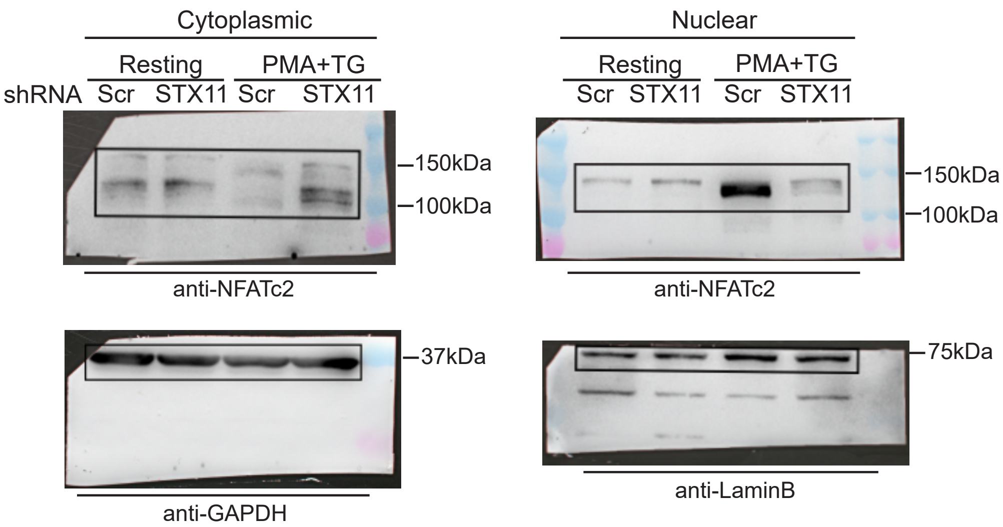

Cytoplasmic
Nuclear
Resting
PMA+TG
Resting
PMA+TG
shRNA
Scr
STX11
Scr
STX11
Scr
STX11
Scr
STX11
150kDa
100kDa
150kDa
100kDa
anti-NFATc2
anti-NFATc2
37kDa
75kDa
anti-GAPDH
anti-LaminB
