# Supplementary material for: Syntaxin11 deficiency inhibits CRAC channel priming to suppress cytotoxicity and gene expression in T lymphocytes independent of membrane trafficking
Source: eLife. 2026 Oct 19;15:RP111335. doi: 10.7554/eLife.111335 (PMC13679927; doi:10.7554/eLife.111335)
Supplement: Supplementary file 11 [file elife-111335-fig4-data4.zip › Gating strategy.pdf]

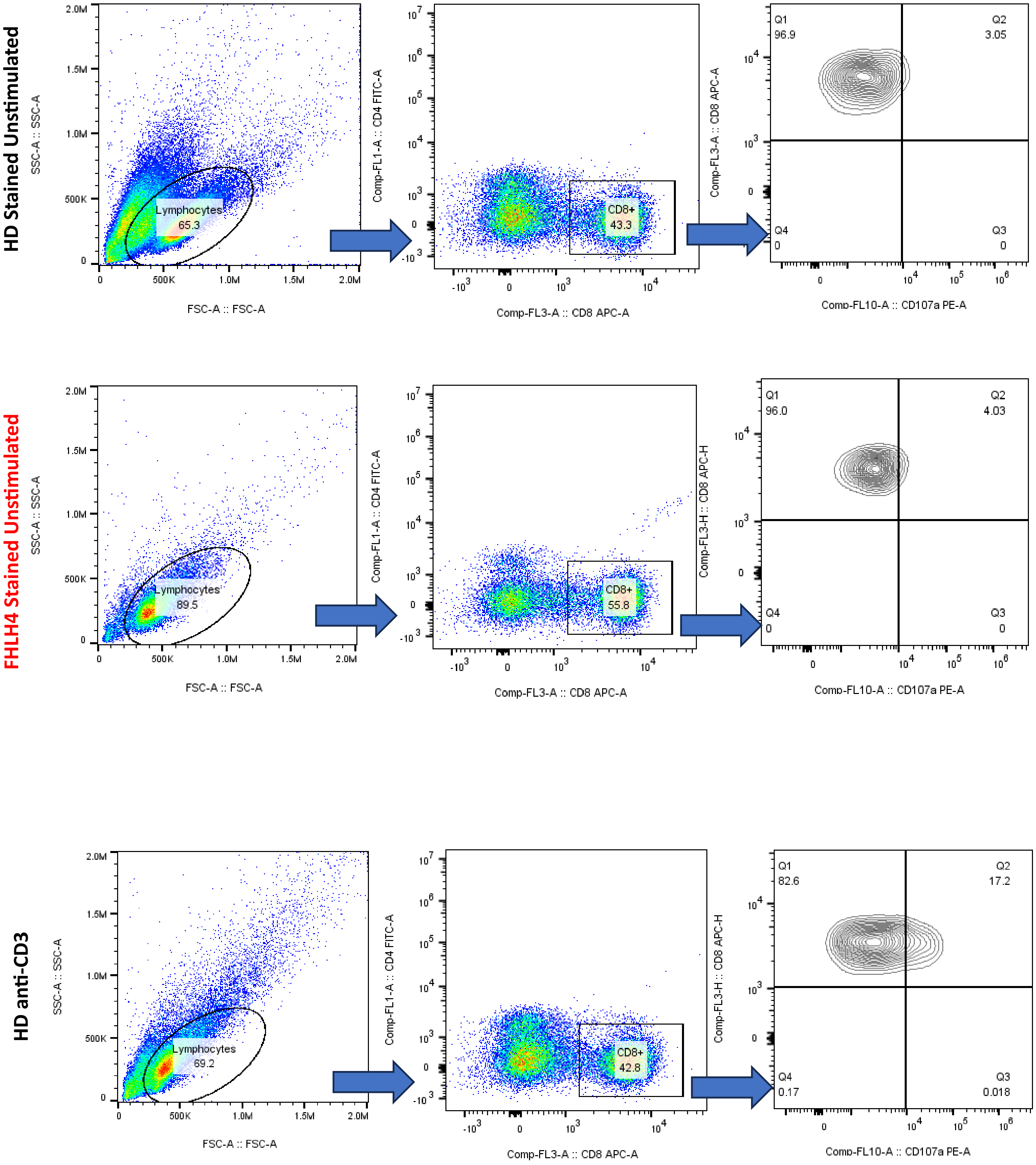

HD Stained Unstimulated
FHLH4 Stained Unstimulated
HD anti-CD3
SSC-A :: SSC-A
FSC-A :: FSC-A
Lymphocytes
65.3
89.5
69.2
Comp-FL1-A :: CD4 FITC-A
Comp-FL3-A :: CD8 APC-A
CD8+
43.3
55.8
42.8
Comp-FL3-A :: CD8 APC-A
Comp-FL3-H :: CD8 APC-H
Comp-FL10-A :: CD107a PE-A
Q1 96.9
Q2 3.05
Q4 0
Q3 0
Q1 96.0
Q2 4.03
Q4 0
Q3 0
Q1 82.6
Q2 17.2
Q4 0.17
Q3 0.018

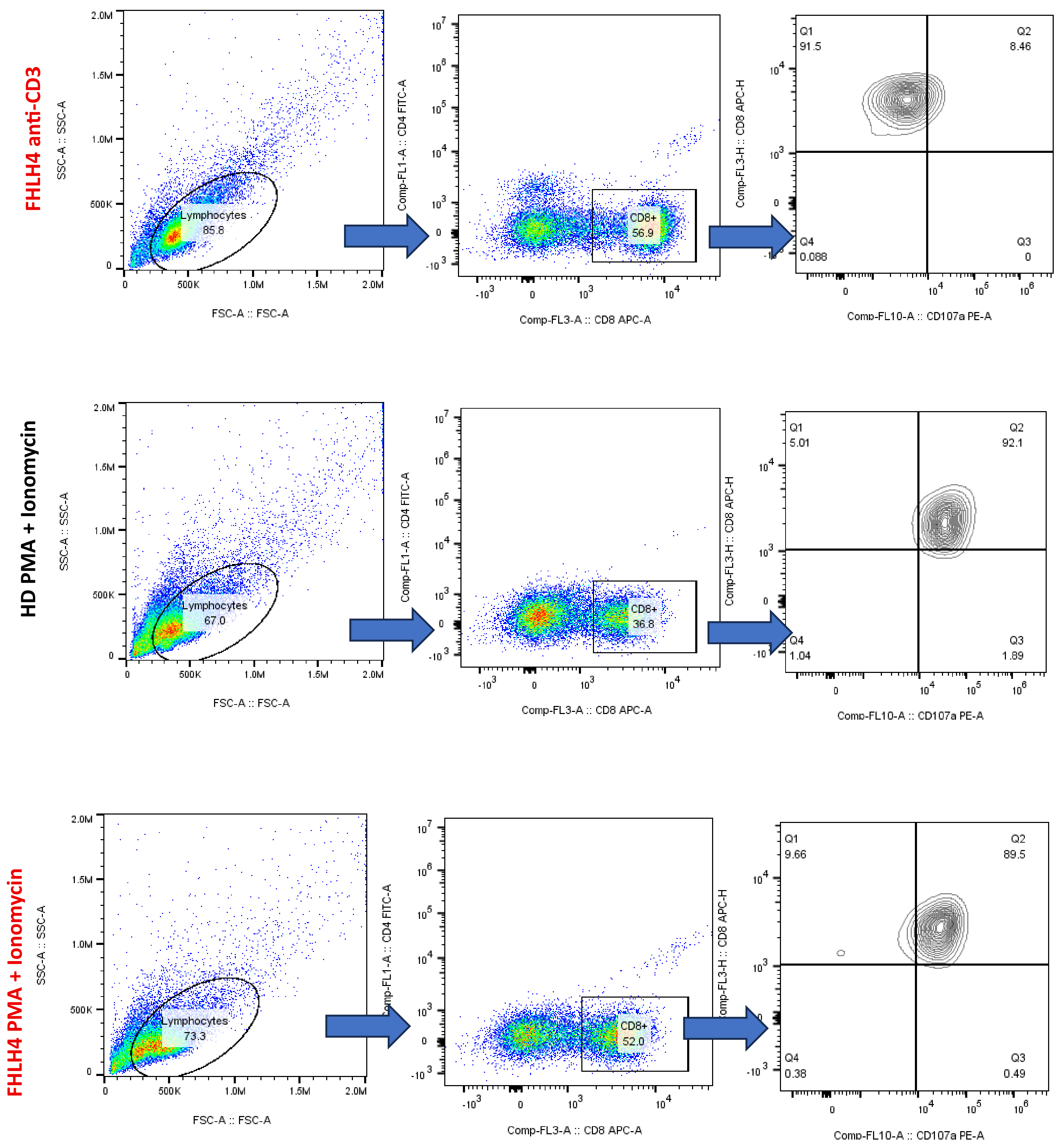

FHLH4 anti-CD3
HD PMA + Ionomycin
FHLH4 PMA + Ionomycin
SSC-A :: SSC-A
FSC-A :: FSC-A
Lymphocytes
85.8
67.0
73.3
Comp-FL1-A :: CD4 FITC-A
Comp-FL3-A :: CD8 APC-A
CD8+
56.9
36.8
52.0
Comp-FL3-H :: CD8 APC-H
Comp-FL10-A :: CD107a PE-A
Q1
91.5
Q2
8.46
Q3
0
Q4
0.088
Q1
5.01
Q2
92.1
Q3
1.89
Q4
1.04
Q1
9.66
Q2
89.5
Q3
0.49
Q4
0.38
