# Supplementary material for: Syntaxin11 deficiency inhibits CRAC channel priming to suppress cytotoxicity and gene expression in T lymphocytes independent of membrane trafficking
Source: eLife. 2026 Oct 19;15:RP111335. doi: 10.7554/eLife.111335 (PMC13679927; doi:10.7554/eLife.111335)
Supplement: Supplementary file 12 [file elife-111335-fig5-data2.zip › Figure 5-source data 2/Figure 5I.pdf]

Figure 5I

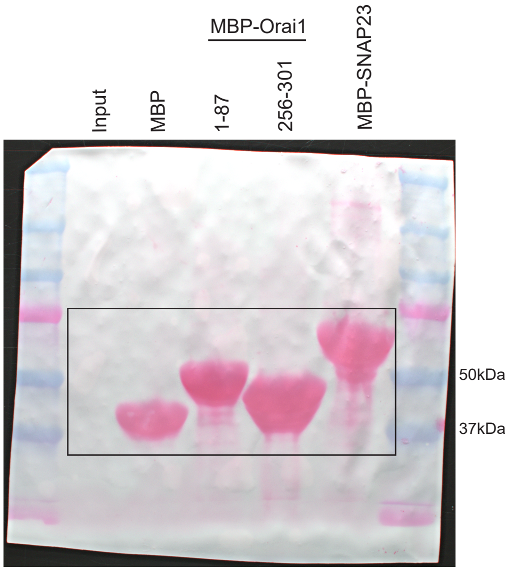

MBP-Orai1
Input
MBP
1-87
256-301
MBP-SNAP23
50kDa
37kDa

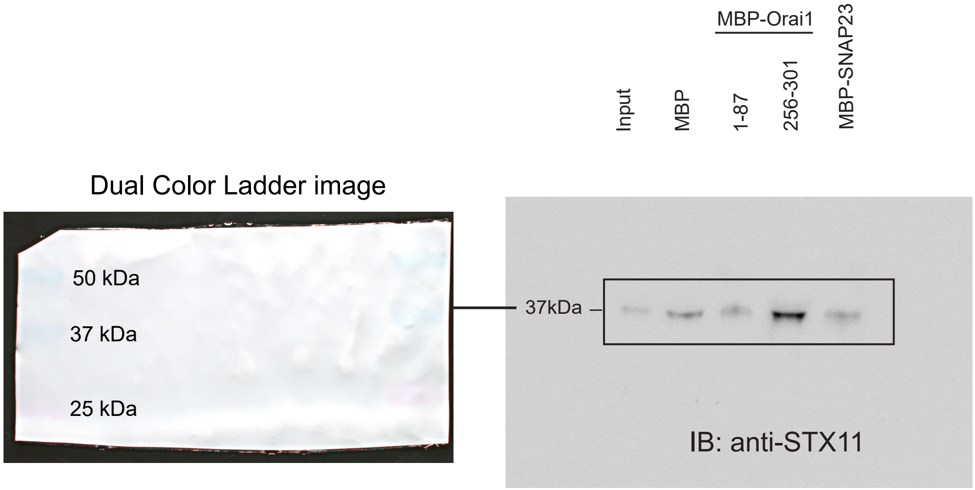

Dual Color Ladder image
50 kDa
37 kDa
25 kDa
MBP-Orai1
Input
MBP
1-87
256-301
MBP-SNAP23
37kDa
IB: anti-STX11
