# Supplementary material for: Syntaxin11 deficiency inhibits CRAC channel priming to suppress cytotoxicity and gene expression in T lymphocytes independent of membrane trafficking
Source: eLife. 2026 Oct 19;15:RP111335. doi: 10.7554/eLife.111335 (PMC13679927; doi:10.7554/eLife.111335)

Figure 5-figure supplement 4(A)

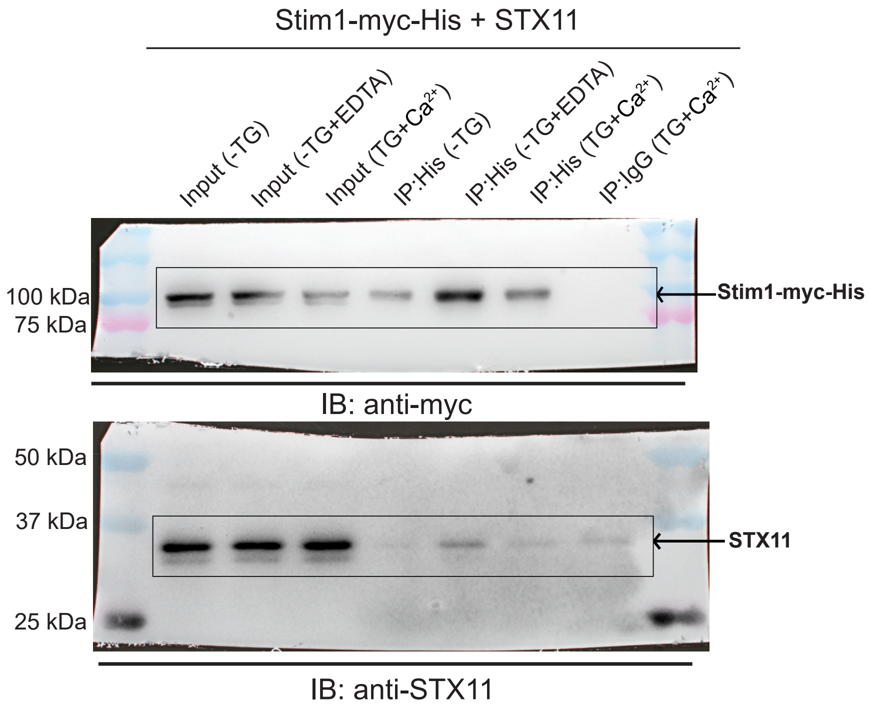

Supplement: Supplementary file 17 [file elife-111335-fig5-figsupp4-data1.zip › Figure 5-figure supplement 4-source data 1/Figure 5-figure supplement 4-source data 1.pdf]
