# Supplementary material for: Syntaxin11 deficiency inhibits CRAC channel priming to suppress cytotoxicity and gene expression in T lymphocytes independent of membrane trafficking
Source: eLife. 2026 Oct 19;15:RP111335. doi: 10.7554/eLife.111335 (PMC13679927; doi:10.7554/eLife.111335)
Supplement: Supplementary file 19 [file elife-111335-fig6-data1.zip › Figure 6-source data 1/Figure 6F.pdf]

Figure 6F

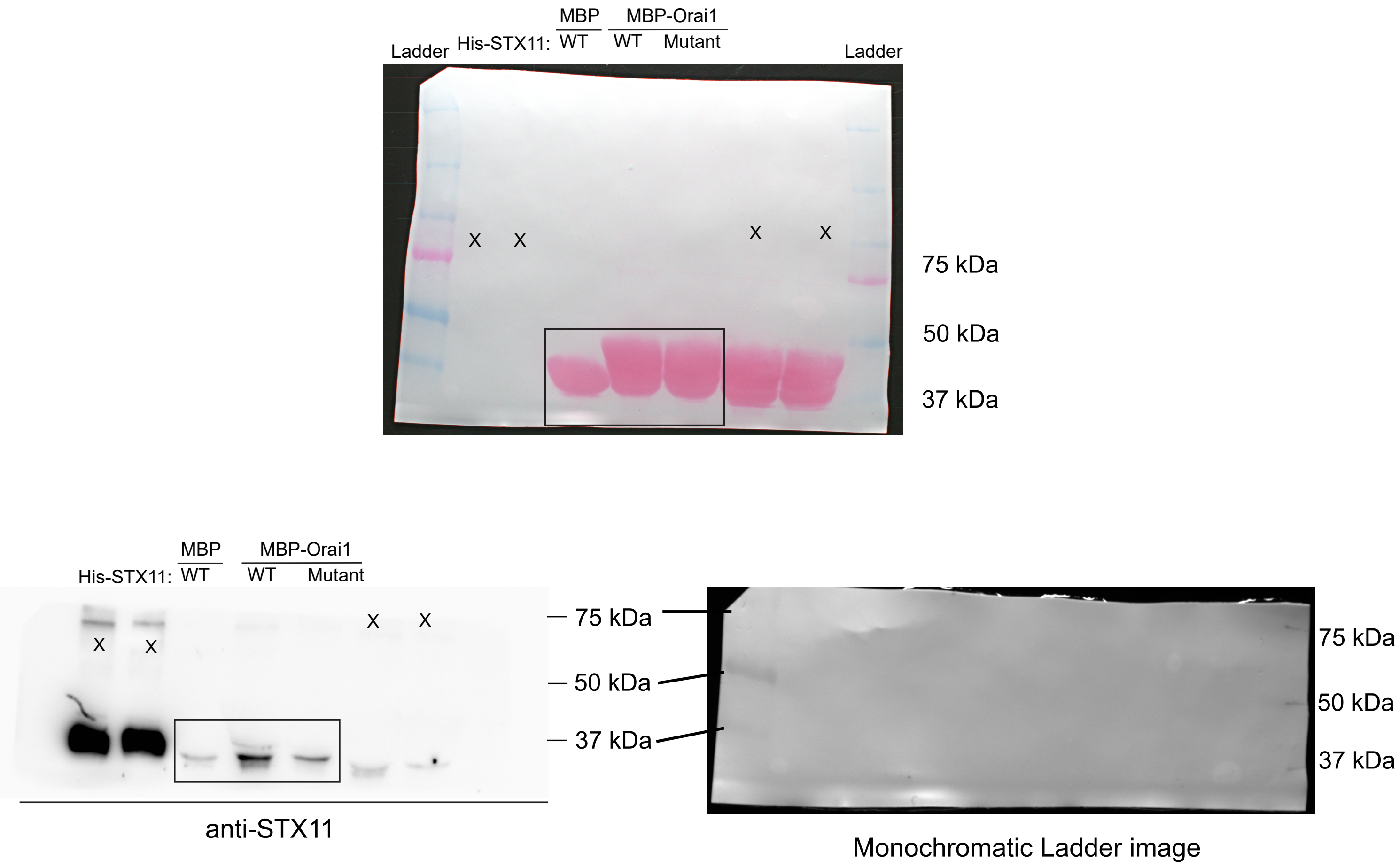

X: Represents lanes not relevant to Figure 6F
