# Supplementary material for: Syntaxin11 deficiency inhibits CRAC channel priming to suppress cytotoxicity and gene expression in T lymphocytes independent of membrane trafficking
Source: eLife. 2026 Oct 19;15:RP111335. doi: 10.7554/eLife.111335 (PMC13679927; doi:10.7554/eLife.111335)
Supplement: Supplementary file 21 [file elife-111335-fig8-data2.zip › Figure 8-source data 2/Figure 8D.pdf]

Figure 8D

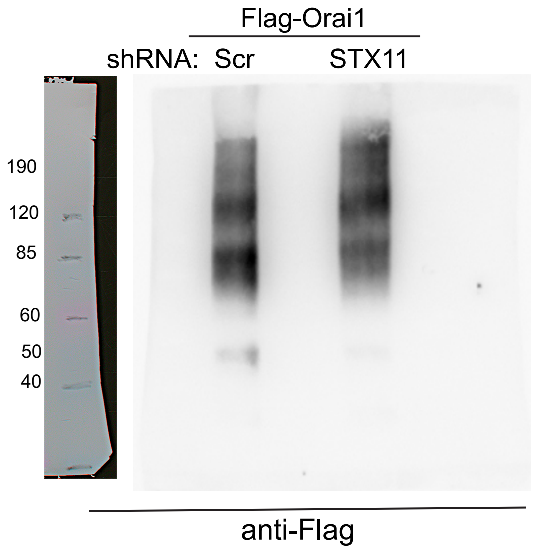

Exposure: 1 min
