# Supplementary material for: Syntaxin11 deficiency inhibits CRAC channel priming to suppress cytotoxicity and gene expression in T lymphocytes independent of membrane trafficking
Source: eLife. 2026 Oct 19;15:RP111335. doi: 10.7554/eLife.111335 (PMC13679927; doi:10.7554/eLife.111335)
Supplement: Supplementary file 23 [file elife-111335-fig8-figsupp1-data1.zip › Figure 8-figure supplement 1-source data 1/Figure 8-figure supplement 1.pdf]

Figure 8-figure supplement 1

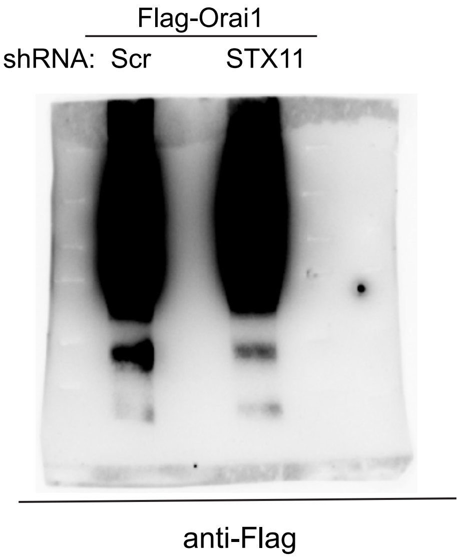

Exposure: 10 min
