# Supplementary material for: Syntaxin11 deficiency inhibits CRAC channel priming to suppress cytotoxicity and gene expression in T lymphocytes independent of membrane trafficking
Source: eLife. 2026 Oct 19;15:RP111335. doi: 10.7554/eLife.111335 (PMC13679927; doi:10.7554/eLife.111335)
Supplement: Peer review history

# Editor report: eLife Assessment

**DOI:** 10.7554/eLife.111335.3.sa0

**Authors:** Kenton J Swartz

**Affiliations:** National Institute of Neurological Disorders and Stroke United States

**Keywords:** Convincing, Important

This study reports a novel function for syntaxin 11, a specialized SNARE protein critical for the immune system whose mutations cause familial hemophagocytic lymphohistiocytosis type 4. The data **convincingly** show that depletion of STX11 impairs store-operated calcium entry in Jurkat T cells and that this defect is recapitulated in primary cells from a patient suffering from the disease; the authors further show that the syntaxin interacts with the pore subunit of the ORAI1 channel and propose that it primes the channel by promoting the assembly of multimers before activation by its endogenous ligand, the ER Ca2+ sensing protein STIM1. This is a conceptually **important** claim that challenges the prevailing view that all structural transitions in ORAI1 are STIM-driven. The high-quality data strongly support the interpretations, but the discussion would be strengthened if the authors suggest alternative mechanisms and mention earlier studies reporting ORAI1 regulation by vesicular trafficking.

# Referee report: Reviewer #1 (Public review)

**DOI:** 10.7554/eLife.111335.3.sa1

Summary:

Patients with STX11 mutations develop familial hemophagocytic lymphohistiocytosis Type 4, a fatal immune disorder marked by defective T and NK cell cytotoxicity and cytokine storm. The conventional explanation attributes this to impaired cytotoxic granule release, but this has never fully accounted for the broader disease picture. This study proposes an alternative mechanism. The authors show that STX11 is required for store-operated calcium entry through ORAI1 channels, which are essential for both cytotoxic killing and NFAT-driven gene expression in T cells. In STX11-deficient cells, ORAI1 currents drop, NFAT nuclear translocation fails, IL-2 expression is suppressed, and degranulation is impaired. These defects are largely rescued by ionomycin or a constitutively active ORAI1 mutant, placing the primary lesion at calcium signaling rather than the fusion machinery. Mechanistically, STX11 binds the C-terminal tail of ORAI1 via its Habc domain and maintains ORAI1 in a state competent for productive assembly prior to STIM1-dependent gating, a step the authors call "priming."

Strengths:

The paper identifies a novel and disease-relevant role for STX11 in calcium channel regulation and raises the possibility of using channel agonists as a therapeutic strategy in the disease. The biochemical and functional data are of high quality and generally consistent with the interpretation. The proposal that a non-conventional syntaxin directly interacts with ion channels to prime its activation is novel and interesting. Additional experiments now exclude the possibility that STX11 acts as a SNARE to sustain calcium fluxes by promoting the delivery of additional functional channels.

Weaknesses:

Previous studies reporting regulation of ORAI1 by vesicular trafficking are ignored and alternative mechanisms are not considered.

# Referee report: Reviewer #2 (Public review)

**DOI:** 10.7554/eLife.111335.3.sa2

Summary:

Vig's lab delineates a critical role for STX11 in CRAC channel function, particularly in the context of the fatal immune disorder familial hemophagocytic lymphohistiocytosis type 4 (FHL4). They demonstrate that Syntaxin 11 directly binds and regulates Orai1, and that STX11 depletion abolishes CRAC currents and downstream signaling. Loss of STX11 reduces IL2 gene expression and impairs degranulation, both of which are rescued by the constitutively active Orai1 mutant H134S, whereas a gain‑of‑function mutant targeting the C‑terminus fails to restore these defects. The authors conclude that STX11 primes Orai1 for optimal local assembly that is independent of STIM1 yet required for CRAC channel gating.

Strengths:

This study is firmly grounded in disease biology and demonstrates that STX11 downregulation leads to profound functional defects. Using a comprehensive suite of methods and analyses, the authors interrogate the co-regulation of STX11 and Orai1 and present a near-complete view of STX11's modulatory role in CRAC channel function and downstream signaling pathways. The figures are clear, and the statistical analyses are rigorous and convincing.

Weaknesses:

The authors conclude that Syntaxin 11 directly binds Orai1. This conclusion is well supported by a multifaceted approach-including co-immunoprecipitation (co-IP), molecular dynamics simulations, co-localization/FRET assays, and targeted mutational analysis-all of which are thoroughly executed. While the interaction appears reasonably strong in co-IP experiments, the STX11-Orai1 interaction is comparatively weaker in pull-down assays, which the authors attribute to instability of the purified His-STX11 protein. A remaining gap is direct evidence of interaction in live cells; this is understandably challenging given that fluorescent tagging of STX11 is not feasible. Fully resolving this question lies beyond the scope of the present study and will require more advanced approaches to capture STX11 binding dynamics.

Comments on revised version.

The authors have addressed all my comments highly satisfactorily!

# Author comment: Author response

**DOI:** 10.7554/eLife.111335.3.sa3

**Authors:** Sritama Datta, Abhikarsh Gupta, Kunal M Jagetiya, Resmi Bera, Vikas R Tiwari, Megumi Yamashita, Atharva R Yande, Abdul Rishad, Vishal Malik, Sreejith Raran-Kurussi, Sandra Ammann, Mohammad Shahrooei, Kalyaneswar Mandal, Ramanathan Sowdhamini, Murali Prakriya, Adish Dani, Monika Vig

**Affiliations:** Tata Institute of Fundamental Research Hyderabad India; Tata Institute of Fundamental Research Hyderabad India; Tata Institute of Fundamental Research Hyderabad India; Tata Institute of Fundamental Research Hyderabad India; National Centre for Biological Sciences Bangalore India; Northwestern University Chicago United States; Tata Institute of Fundamental Research Hyderabad India; Tata Instituteof Fundamental Research, Hyderabad Hyderabad India; Tata Institute of Fundamental Research, Hyderabad Hyderabad India; Tata Institute for Fundamental Research Hyderabad India; University of Freiburg Freiburg Germany; Dr. Shahrooei Lab Tehran Islamic Republic of Iran; Tata Institute of Fundamental Research Hyderabad India; National Centre for Biological Sciences (TIFR) Bangalore India; Northwestern University Chicago United States; Tata Institute of Fundamental Research Hyderabad India; Tata Institute of Fundamental Research Hyderabad India

The following is the authors’ response to the original reviews.

**eLife Assessment**

This study reports a novel function for syntaxin 11, a specialized SNARE protein critical for the immune system whose mutations cause familial hemophagocytic lymphohistiocytosis type 4. The data convincingly show that depletion of STX11 impairs store-operated calcium entry in Jurkat T cells and that this defect is recapitulated in primary cells from a patient suffering from the disease; the authors further show that the syntaxin interacts with the pore subunit of the ORAI1 channel and propose that it primes the channel by promoting the assembly of multimers before activation by its endogenous ligand, the ER Ca2+ sensing protein STIM1. This is a conceptually important claim that challenges the prevailing view that all structural transitions in ORAI1 are STIM-driven. The data are high-quality and broadly consistent with the interpretation, but alternative mechanisms for the defects are not considered; additional work should rule out vesicular trafficking, discuss other mechanisms, and address methodological issues.

We thank the editor and reviewers for assessing our work. We have now included additional experiments in a new main Figure 2, which directly rule out any general or Orai1 plasma membrane trafficking defects in Syntaxin11-depleted cells. There are additional experiments and/or analysis in many other figures, throughout the paper. We have included new and missing methods, quantifications and calibrations, and provided response to each of the reviewer’s comments below.

**Public Reviews:**

**Reviewer #1 (Public review):**

Summary:

Patients with STX11 mutations develop familial hemophagocytic lymphohistiocytosis Type 4, a fatal immune disorder marked by defective T and NK cell cytotoxicity and cytokine storm. The conventional explanation attributes this to impaired cytotoxic granule release, but this has never fully accounted for the broader disease picture. This study proposes an alternative mechanism. The authors show that STX11 is required for store-operated calcium entry through ORAI1 channels, which are essential for both cytotoxic killing and NFAT-driven gene expression in T cells. In STX11-deficient cells, ORAI1 currents drop, NFAT nuclear translocation fails, IL-2 expression is suppressed, and degranulation is impaired. These defects are largely rescued by ionomycin or a constitutively active ORAI1 mutant, placing the primary lesion at calcium signaling rather than the fusion machinery. Mechanistically, STX11 binds the C-terminal tail of ORAI1 via its Habc domain and maintains ORAI1 in a state competent for productive assembly prior to STIM1-dependent gating, a step the authors call "priming."

Strengths:

The paper identifies a novel and disease-relevant role for STX11 in calcium channel regulation and raises the possibility of using channel agonists as a therapeutic strategy in the disease. The biochemical and functional data are of high quality and generally consistent with the interpretation. The proposal that a non-conventional syntaxin directly interacts with ion channels to prime its activation is novel and interesting.

Weaknesses:

For readers to appreciate the value of patient experiments derived from a single individual, the authors should quote prior studies showing that STX11 protein levels are abolished in all known human STX11 mutations. The priming model, while functionally well-supported, rests on indirect structural evidence, and the precise conformational transition involved remains to be defined. These are acknowledged limitations, but alternate mechanisms have not been explored and formally excluded. More direct evidence should be provided to exclude the possibility that STX11 could act as a conventional SNARE and sustain calcium fluxes by promoting the delivery of additional ORAI1 channels from vesicles.

In the revised version, we have included references for all those prior STX11 human mutations that have been biochemically characterized till date. The reviewer has correctly pointed out that STX11 protein levels were almost abolished in almost all previously reported mutations. See line 168-173. Therefore, the prior STX11 patient mutations are essentially comparable to the frameshift mutation characterized in this study, in terms of STX11 protein depletion and, therefore, the mechanisms underlying the phenotypic defects reported here as well as earlier. We, therefore, believe that our data from even a single FHLH4 patient, with severely depleted STX11 levels, and additional knockdown studies across three different cell lines, are representative of majority of STX11 mutant FHLH4 patients that have been previously characterized.

Regarding the Reviewers’ concern that absence of STX11 as a conventional SNARE could affect Orai1 channel delivery from intracellular vesicles. We would like to point out the following:

(1) In Miao et al. 2013 (1), Figure 3C-D, we showed that expression of a dominant-negative mutant of NSF, a non-redundant protein in vesicle trafficking, impaired vesicle trafficking but did not affect SOCE. This experiment had essentially ruled out a role for vesicle trafficking in SOCE. In the same paper, we had also shown that Orai1 levels in the PM do not increase post-store depletion (Figure 3-figure supplement 2).

(2) SNAP23/25 form a four helical bundle with R- and Q-SNAREs in orchestrating vesicle fusion. In this paper, we have ruled out a direct role for SNAP23, SNAP25 and SNAP29 in SOCE (Figure 2-figure supplement 3).

(3) In v1 of this manuscript, we had shown that U2OS cells stably expressing Orai1-BBS-YFP have identical levels of Orai1 in the PM with and without STX11 depletion (Supplementary Figure 3B). This showed that the biosynthesis or delivery of Orai1 to the PM is not affected by STX11 depletion. The levels were also assessed in store-depleted U2OS cells but not included because in Miao et al. 2013 we had already established that levels of PM Orai1 remain essentially equal in resting versus store-depleted cells.

In the revised version, we have included the data from store-depleted cells in U2OS and also done quantification of PM Orai1 in HEK293 and Jurkat T cells. In addition, we have added three independent membrane trafficking/ vesicle secretion assays performed in STX11-depleted cells (new Figure 2 and associated supplements). In all cases, we find no evidence of Orai1 in intracellular vesicles or a general defect in membrane trafficking/ secretion in STX11-depleted cells. Orai1 is constitutively and stably expressed in the PM in resting as well as store-depleted cells in three different cell lines.

(4) Most importantly, in Figure 7I-J of this manuscript, we showed that calcium influx from a constitutively active mutant Orai1 (Orai H134S) is identical between STX11-depleted and scramble control cells. If wildtype Orai1 was indeed stuck in vesicles in STX11-depleted cells, then how would mutant H134S Orai1 be able to rescue the defect in SOCE? We have included the quantification of PM levels of Orai1 mutants w.r.t WT Orai1 in new Figure 8-figure supplement 3B and 3D.

In summary, we have now done several new experiments to directly measure Orai1 levels in the PM and general vesicle trafficking assays in HEK293 and Jurkat T cells and have found no defects in these upon STX11 depletion.

Regarding STX11 induced precise conformational transition, we are trying to setup collaborations with scientists who might be able to visualize this *i*n situ. Please note that while purification of isolated pore subunits of ion channels followed by crystallization or expression in synthetic membranes for cryo-EM is currently considered a gold standard in the analysis of ion channel pore subunits, we have shown that ion channels are dynamic macromolecular complexes, in vivo (2), where synaptic proteins dynamically bind to induce conformational changes and affect their stoichiometry (2). Please also see (3) and (4). More advanced approaches, therefore, need to be developed to enable visualization of the dynamics of ion channel macromolecular complexes in their native environment in situ. In the absence of such approaches, the structural insights obtained from detergent-purified isolated subunits will remain incomplete.

**Reviewer #2 (Public review):**

Summary:

Vig's lab delineates a critical role for STX11 in CRAC channel function, particularly in the context of the fatal immune disorder familial hemophagocytic lymphohistiocytosis type 4 (FHL4). They demonstrate that Syntaxin 11 directly binds and regulates Orai1, and that STX11 depletion abolishes CRAC currents and downstream signaling. Loss of STX11 reduces IL2 gene expression and impairs degranulation, both of which are rescued by the constitutively active Orai1 mutant H134S, whereas a gain‑of‑function mutant targeting the C‑terminus fails to restore these defects. The authors conclude that STX11 primes Orai1 for optimal local assembly that is independent of STIM1 yet required for CRAC channel gating.

Strengths:

This study is firmly grounded in disease biology and demonstrates that STX11 downregulation leads to profound functional defects. Using a comprehensive suite of methods and analyses, the authors interrogate the co-regulation of STX11 and Orai1 and present a near-complete view of STX11's modulatory role in CRAC channel function and downstream signaling pathways. The figures are clear, and the statistical analyses are rigorous and convincing.

Weaknesses:

The authors conclude that Syntaxin 11 directly binds Orai1. This conclusion is well supported by a multifaceted approach, including co-immunoprecipitation (co-IP), molecular dynamics simulations, co-localization/FRET assays, and targeted mutational analysis-all of which are thoroughly executed. While the interaction appears reasonably strong in co-IP experiments, the STX11-Orai1 interaction is comparatively weaker in pull-down assays, which the authors attribute to instability of the purified His-STX11 protein. A remaining gap is direct evidence of interaction in live cells; this is understandably challenging given that fluorescent tagging of STX11 is not feasible. Fully resolving this question lies beyond the scope of the present study and will require more advanced approaches to capture STX11 binding dynamics.

We thank the reviewer for acknowledging that analysis of the dynamic binding of STX11 will require standardization of advanced techniques which are beyond the scope of the present study. We plan to continue developing methods that will allow us to visualize the binding and unbinding of STX11 to Orai1 in vivo.

**Recommendations for the authors:**

**Reviewer #1 (Recommendations for the authors):**

Mechanistic issues:

(1) More direct evidence should be provided to exclude the possibility that STX11 could act as a conventional SNARE and sustain calcium fluxes by promoting the delivery of additional functional channels, stored in secretory vesicles or recycling endosomes, to the membrane. A significant fraction of the ORAI1 channel is in vesicles, and the mobilization of this intracellular pool regulates the rates of calcium fluxes in HEK-293 cells (PMID 26116575) and effector T cells (PMID: 35217583). Mobilization of this pool could account for part or all of the functional effects reported here. The only evidence that STX11 depletion does not impact the plasma membrane availability of the channel relies on one single flow cytometry profile (Supplementary Figure 3). This experiment is performed in U2OS cells stably expressing a fusion protein containing an extracellular bungarotoxin site. This cellular system is only used here; the other data are obtained either in HEK-293 cells or in Jurkat T cells. The level of endogenous STX11 in U2OS cells is unknown, and the efficiency of protein depletion has not been assessed. The efficiency of depletion should be shown, and the total number of channels assessed by comparing the expression levels of permeabilized and non-permeabilized cells. A flow cytometry profile of cells treated with thapsigargin should be included to match the experimental conditions of functional recordings. It would be valuable to repeat this experiment in Jurkat T cells by expressing ectopically the channel tagged on the extracellular side. To further exclude the involvement of vesicular trafficking, the authors should also evaluate the contribution of VAMP8, as this R-SNARE has been proposed to interact with STX11 to regulate the exocytosis of specialized granules in cytotoxic T cells (PMID: 26124288).

We have not observed significant levels of intracellular Orai1, as described in the PMID 26116575 paper. Multiple technical reasons could explain the artefactual appearance of intracellular Orai1. HEK293 is an embryonic kidney cell line, with most cells showing a distinct spindle shape and filopodia as shown on the ATCC website (https://www.atcc.org/products/crl-1573). The HEK cells shown throughout the Hodeify et al. 2015 paper (PMID 26116575) lack this typical morphology that majority of the HEK cells should show. Transfecting cells with high amounts of DNA and liposomes can severely affect the health and morphology of the cells leading to artifacts where PM proteins appear to be stuck intracellularly. The plasma membrane of the cell in movie 1 of PMID 26116575, for instance, also shows membrane blebs or membrane ruffles. The authors should have used a PM marker such as WGA (wheat germ agglutinin) to distinguish PM Orai1 from any intracellular Orai1 to establish whether what appear as intracellular Orai1 vesicles are not blebs of PM Orai1. Similarly, no endo/exocytotic vesicle marker is used to distinguish them from Orai1’s presence in apoptotic vesicles of unhealthy cells. In view of the overall abnormal morphology and any PM or intracellular vesicle marker, the claim that Orai1 resides in intracellular vesicles is unfounded.

Similarly, the PMID: 35217583, quoted by reviewer #1, is about in vitro differentiated primary mouse T cells. The study lacks any detail of the generation of the HA-tagged mouse Orai1 plasmid, used in the study, or even a back reference to show how this plasmid was validated for normal expression in primary T cells earlier. Ectopic expression of CMV promoter-driven plasmids in mouse primary T cells is extremely challenging and often results in poor cell health and incomplete and selective expression in only 5-10% of cells. It is unclear if the same HA-tagged human Orai1 plasmid that was used in PMID 26116575 is being used in this study to express in primary mouse cells. In the absence of all this information, it is unclear whether there was an issue with the generation of a new HA-tagged mouse Orai1 construct, which made the protein get stuck intracellularly. Or potentially the expression of a human protein in mouse primary T cells is the problem. The functional verification of the construct by showing rescue of SOCE in Orai1-deficient primary mouse cells is an absolutely essential control but is missing. In the absence of these controls, one cannot disregard decades of robust data on the localization of Orai1 in the PM from multiple labs and papers that have established its PM localization conclusively (5) (6).

Most importantly, in both the PMID 26116575 and PMID: 35217583, HA tagged-Orai1 is detected using a bivalent antibody, followed by secondary antibody. It is well known that cross-linking of cell surface receptors or proteins using bivalent antibodies has a major caveat that involves antibody-mediated clustering and capping, especially in lymphocytes, which typically induces rapid internalization of the entire antigen-antibody complex. The paper by Sekine-Aizawa et al., in 2004, showed that tagging of receptors/ channels with bungarotoxin binding site (BBS) followed by labelling with bungarotoxin (BTX) bypasses this confounding factor and, therefore, allows accurate estimation and localization of PM versus intracellular proteins. The artefactual bivalent antibody-induced endocytosis continues even when cells are incubated on ice as endocytosis is only slowed but not stopped on ice.

Due to this challenge, we have generated BBS tagged Orai1-YFP. We use flow cytometry to show PM Orai1 because it is an unbiased and quantitative way of showing surface Orai1 expression (estimated by measuring the intensity of surface-bound BTX), simultaneously, in thousands of cells with no potential for visual bias in the selection and imaging of cells. BTX labelling is done on ice and cells are washed and fixed right after labelling with BTX-A647 to stop endocytosis. In the revised version, we have used both complementary approaches of flow cytometry and microscopy, showing representative images of cells, alongside quantifications. All new experiments were performed in HEK293 and Jurkat T cells to estimate surface versus total expression in a new main Figure 2. The post-store-depletion data have been added to the existing U2OS experiment in new Figure 2-figure supplement 2A-E.

Our BBS-tagged Orai1 construct also has a YFP tag at the C-terminus. Since the flow cytometry experiment involves gating on YFP-positive cells, the total number of channels (biosynthesis) can be compared by looking at the YFP intensities in the scramble versus STX11-depleted groups. These data have now been included in the previous and new experiments. In none of the cases could we detect any difference in YFP or BTX-A647 intensities, pre- or post-store-depletion in scr or STX11-depleted cells which would indicate defects in biosynthesis or Orai1’s presence in vesicles in any group.

Regarding a role for VAMP8, in Miao et al. eLIFE 2013 (1), Figure 3C-D, we showed that expression of a dominant negative mutant of NSF, a non-redundant protein in the vesicle trafficking pathway, impaired Transferrin receptor recycling within 20 hours but did not affect SOCE at all. This experiment had conclusively ruled out any role for vesicle trafficking in SOCE and therefore assessment of the role of each of the individual proteins involved in membrane trafficking becomes redundant. We have additionally ruled out a role for SNAP23/ SNAP25/ SNAP29 (old supplementary Figure 12, new Figure 2-figure supplement 3A-C). SNAP23/25 form a four helical bundle with most R-SNARE and Q-SNAREs to orchestrate vesicle fusion. R-SNAREs, typically, need SNAP23/25 to interact with Q-SNAREs. Since a role for these non-redundant proteins has also been ruled out by us, it is unlikely that VAMP8 plays a role in modulating the effects of STX11 in SOCE.

(2) Both ORAI1 and STX11 are S-Acylated on cysteine residues, and this post-translational modification promotes their recruitment to the immune synapse (PMID: 24910990, 34913437). One possibility that should be discussed is that STX11 could enhance the recruitment of the ORAI1 channel into lipid domains rich in cholesterol, thereby favoring its activation. This type of priming would still require direct interaction between the two proteins but involve a different mechanism than the one discussed by the authors. This could be experimentally tested by expressing a STX11 mutant lacking the cysteine residues required for its S-Acylation. It would also be interesting to test whether depletion of STX11 impairs the recruitment of ORAI1 to the immune synapse forming between Jurkat T cells and antigen-presenting cells.

In our experiments reported in this paper, we have used soluble anti-CD3 as well as plate-coated anti-CD3 in combination with soluble anti-CD28 to stimulate Jurkat and primary T cells. These antibodies are routinely used to stimulate T cells and this type of stimulus doesn’t depend on the formation of a classical immune synapse with an antigen-presenting cell (APC). Despite the absence of synapse, the T cells get fully activated and functional, as seen by NFAT translocation and secretion of cytokines, such as IL-2 in new Figure 4. Therefore, whether there is a defect in the recruitment of Orai1, or STX11, to T cell synapse formed with an APC is not within the scope of this study. Furthermore, accurate analysis of protein localization within the immune synapse requires a dedicated study employing sub-diffraction resolution microscopy approaches.

Regarding PMID: 24910990, and the mechanism of recruitment of STX11 to the membranes. We believe this remains unknown. The frameshift mutant used in our study lacked all terminal cysteines, which have been earlier proposed to be crucial for membrane targeting, as well as a terminal part of the SNARE domain and yet it localized to the PM just as well as wild-type STX11 (See new Figure 5E). We have added this result in the text line 302-305 and removed the line stating that post-translational modifications of the terminal cysteines target STX11 to the PM as previously claimed in PMID: 24910990 and mentioned in v1 of this paper. In view of these new data, it currently remains unknown whether potential attachment to PIP2 in PM via various basic residues, spread throughout the sequence (7, 8), or binding to another protein targets STX11 to the PM (PMID: 26771955). There is no obvious poly-basic stretch in STX11 sequence, therefore, a systematic and focused deletion and mutagenesis study will be needed to individually assess the above possibilities which is outside the scope of the present study.

Methodological issues

(3) Since STX11 colocalize with Orai1 already in basal conditions, independently of STIM1, it could influence basal calcium levels. This cannot be appreciated from the data presented, because all the SOCE protocols start in calcium-free conditions, preventing baseline comparison between WT and STX11-deficient cells. A potential difference in basal calcium levels should be explored, and the impact of STX11 depletion on basal calcium fluxes should be documented by calcium shifts (2 mM → 0 mM → 2 mM) or manganese quenching approaches.

The cells are typically loaded with Fura2 in 2mM calcium containing Ringer’s buffer. We switch the cells to 0mM right at the start of the SOCE protocol and start imaging within 5-10 seconds. Therefore, in our experience, the baselines of scramble versus STX11-depleted cells should show a difference even in the SOCE protocol if the basal calcium levels are affected because Fura2 is already present and bound to basal calcium present in the cytosol at the start of the protocol. Still, we have done the experiment suggested by the reviewer as shown in Author response image 1. The assay started with cells in 2 mM extracellular Ca^2+^ followed by addition of 10 mM EGTA, which according to the following equation quenches the 2 mM extracellular Ca^2+^ (here).

$$\displaystyle [\mathrm{Ca} 2+]_{\text {Free}}=K d *\left(\frac{[\mathrm{Ca} 2+]_{\text {Total}}}{[\text {EGTA}]_{\text {Total}}-[\text {Ca} 2+]_{\text {Total}}}\right)$$

where, [Ca2+]_Free_ is the free/unbound Ca2+, [Ca2+]_Total_ is the total Ca2+, [EGTA]_Total_ is the total EGTA concentration and Kd is the dissociation constant between Ca2+ and EGTA at 37°C and pH 7.4.

To confirm complete sequestration of the extracellular Ca^2+^, we also repeated the assay with 20 mM EGTA but did not notice any difference between the 10 mM and 20 mM EGTA conditions. We do not see any differences in basal calcium, under any condition, between Scr and STX11 shRNA treated cells HEK or Jurkat T cells.

**Author response image 1.** Representative Fura-2 traces of Scr (black) and STX11 (red) shRNA-treated HEK293 (A) and Jurkat (B) cells, where the cells were incubated with 2 mM Ca^2+^ followed by addition of 10 mM EGTA to quench the 2 mM Ca^2+^. Since we do not have access to a perfusion system, we could not test Fura2 response after re-addition of 2mM calcium to the existing EGTA and Ca^2+^ mixture. However, the transition from 0mM to 2mM is already shown in Figure 8.

(4) The quantification of the calcium imaging data is problematic and requires clarification. In most figures, the data are shown normalized to the control condition. According to the method section (lines 721-724), 100% is the maximum value of the scramble shRNA group (amongst the three experiments). But what was measured here? The slope during calcium readmission? The peak amplitude after calcium readmission? Expressed as a ratio or as calcium values? The recordings are presented in micromolar calcium concentration. This implies calibration, but a calibration procedure is not mentioned. Please clarify. For the recordings of constitutive calcium entry in Figure 7, this normalization is not performed, and the data are expressed as ratio values. Here, it looks like the parameter quantified and compared is the absolute ratio value after calcium readmission. This is inappropriate. The trace in Figure 7G shows that the basal levels differ by more than two ratio units between control and STX11-depleted cells. Normalizing the data in Figure 7G to the basal ratio value would show no difference in the peak response amplitude between the two conditions. These data should be re-analyzed, and both the slope and the amplitude of the response should be presented, with statistics performed on independent recordings, not on individual cells pooled from different experiments. Cells from the same recording are not experimentally independent samples but rather replicates of the same experiment.

We have updated the relevant method section with more details in lines 823-858. The peak amplitude after calcium readmission was measured and compared to the baseline as described in the updated methods. The Fura 2 calibration method has also been added to the revised version, we apologize for this omission earlier. Separate calibration was done for Fura 2 experiments in all figures except Figure 7 from version 1 (new Figure 8). The experiments in new figure 8 were done using a different objective (20X, water) and, therefore, although a separate calibration was done for these experiments it was not applied to the data. We apologise for this omission on our part and have now applied the respective calibration to these experiments.

The trace in Figure 7G of version 1 should look different even in 0mM calcium in our opinion. The reason is the same as that explained in point 3 above; CAD-mediated constitutive activation of Orai1 is one of the strongest. The cells, even when they are being loaded with Fura2 in Ringer’s buffer with 2mM calcium, are constitutively recruiting calcium ions. This should result in a shift in Fura2 excitation due to higher levels of basal calcium. When the cells are switched to 0mM calcium and imaged within 4-5 seconds, the intracellular Fura 2 is still bound to all this extra calcium in the cytosol and therefore the baselines should show a significant difference. If the cells were imaged for several minutes in 0mM calcium, we might have seen the difference in basal ratios slowly reducing. However, we switched to 2mM calcium within 120sec. At this point any free Fura2 would be expected to bind incoming calcium again. For the same reason, the cytosol of Scr cells which would still have relatively higher levels of intracellular calcium concentration compared to STX11-depleted cells will show a smaller further increase in 2mM due to calcium-dependent inhibition of CRAC currents within the time frames we have measured. We have now added the Fura-2-calibrated response in the new Figure 8G-H. We have shown below the baseline-subtracted (normalized) response for Figure 8G. As you can see, there is still a significant difference in 2mM calcium between Scr and STX11-depleted cells but in this representation the important difference at 0mM is masked, we have therefore chosen to retain the original figure with Fura-calibrated values at 0 as well as 2mM calcium in Figure 8G-H.

The cells shown in the old Figure 7G of version 1 were not from the same recording but from three different experiments. Because the cells were imaged with 20X objective in these experiments to allow selection of Orai1-CFP or mutant Orai1-CFP and CAD-YFP double-positive cells, the number of cells analyzed per experiment was less compared to other experiments. We have now shown Fura 2 calibrated values in the new Figure 8G-L. We have also re-done the statistical analysis on three independent experiments from each. As shown in Author response image 2, the difference is still statistically significant whether we show merged cells from all three experiments or single representative experiment out of three repeats. We believe merged cells have more information to offer and therefore have retained the same figures with the original analysis in the main Figure 8G-L.

**Author response image 2.** Box plots representing quantification of individual repeats of constitutive calcium influx in Scr and STX11 shRNA-treated HEK293 cells expressing YFP-CAD and Orai1-CFP without (A) and with baseline subtraction (B). (C-D) Quantification of individual repeats of Scr and STX11 shRNA-treated HEK293 cells expressing Orai1-H134S (C) and Orai1-ANSGA (D) mutants.

(5) Quantification of pull-down experiments. The binding data in Figures 4F, 5F, and 5J are presented largely qualitatively. Densitometric quantification with statistical comparisons across wild-type and mutant conditions would make these results more convincing, particularly given that the authors themselves acknowledge the interaction appears relatively weak in vitro.

This has been done and included alongside the respective panels in the new Figure 6G and 6L (for old Figure 5F and 5J of version 1, where differences appeared relatively small in some experiments). The differences across lanes in both figures and their repeats were statistically significant.

Figure 4F showed a clear and visually significant difference in binding across lanes and repeats and therefore no quantification is needed for these experiments in our opinion.

Limitations of the study and mechanistic inferences.

(7) Interpretation of the ORAI:ORAI FRET and crosslinking data. STX11 depletion increases basal ORAI:ORAI FRET (Figure 7A-C) and shifts crosslinked species toward higher molecular weights (Figure 7D-E). The authors interpret this as ORAI1 being trapped in an unprimed state, but higher FRET and higher-order species would conventionally suggest increased rather than decreased assembly. The paper needs a clearer mechanistic explanation of what "unprimed" looks like structurally. Is this aberrant crowding, non-productive oligomerization, or something else? The distinction between a change in intermolecular distance within existing oligomers versus an increase in oligomer density matters here and should be addressed.

Higher ORAI: ORAI FRET and a shift in the size of crosslinked Orai1 oligomers, when analyzed together, suggests formation of ‘non-functional’ higher-order oligomers. Higher order does not necessarily translate to better function in the case of ion channels, it can also lead to non-selectivity or formation of ‘non-productive’ oligomers, as mentioned by the reviewer. It was shown by us earlier in Li et al. (2016) (2) that bigger oligomer size revealed by higher number of photobleaching steps of Orai1 did not translate to better function but led to non-selectivity.

In new Figure 8, crosslinking with BS3, which has a spacer arm and working distance of ~11 Å, very likely reflects a change in the number of subunits within individual oligomers and not crosslinking of independent existing oligomers. This is because we show that neither total Orai1 expression nor Orai1 expression in the PM change in any group in new Figure 2. FRET works best within 1 to 10 nm distance, and therefore, in theory, can lead to energy transfer between neighbouring Orai1 oligomers in high Orai1-expressing cells. However, because there was no change in Orai1 abundance in the PM (new Figure 2) or distribution within PM (new Figure 7E,F,J,L) of any group, FRET changes also likely reflect intra-oligomer changes rather than inter-oligomer interactions. FRET changes can also arise from a change in the respective orientation of fluorophore pairs but when analyzed together with crosslinking studies, changes in pore assembly likely coincide with conformational shifts in Orai1 protomers. Furthermore, FRET has been used earlier to show shifts in conformation of other ion channels (9). Therefore, we believe that, when used together, these two approaches strongly suggest an intermediate conformational state along with a change in number of Orai1 subunits per channel since there was no evidence of overcrowding in the PM or obvious segregation of Orai1 in specific regions of PM in new Figures 2 and 7E,F,J,L.

We could not assess whether the oligomers of Orai1 formed in the absence of STX11 possess an intact pore. The presence or absence of pore in STX11-depleted cells will require extraction of Orai1 oligomers from native membranes and performing systematic structural analysis using cryo-EM or related approaches which is outside the scope of this study.

(8) The ANSGA versus H134S discrepancy. H134S ORAI1 rescues calcium influx in STX11-depleted cells (Figure 7I-J), but the ANSGA mutant does not (Figure 7K-L). The authors conclude from this that STX11 induces molecular shifts within ORAI1 transmembrane helices, and not in its C-terminal tail. This is an important mechanistic inference that needs more discussion. What does this imply about the conformational state of primed ORAI1? And why is straightening of the tails not sufficient for full opening without the correct TM helix arrangement? This distinction has implications for how the STX11-ORAI1 interaction should be modelled and should be engaged with more thoroughly.

There is no discrepancy here, please also see our response to reviewer 2’s comment #4. The experiment implies that the conformational state of primed Orai1 involves shifts in the TM region of Orai1 and is different from unprimed state. The structural similarities between H134S and ANSGA Orai1 mutants have not been formally established. Unlike H134S, no structure exists for the ANSGA mutant. In the absence of this, it is impossible to comment on whether the two constitutively active mutants are structurally comparable or whether there are multiple ways to stabilize open states of CRAC channel pore, especially when using TM mutants of Orai1.

The goal of this experiment was to determine what kinds of structural shits STX11 potentially induces in native Orai1. Using previously characterized constitutively active mutants and fusion proteins from the CRAC field, we have ruled out a potential role for STX11 in simply changing the orientation of Orai1 C-terminal tails. A discussion on the topic of why tail straightening of Orai1 is insufficient to open Orai1 is outside the scope of this paper. As pointed by reviewer 2, it is possible that C-term tails already exist pointing towards the cytosol in native, resting Orai1, although this has not been shown in any study using structure of full-length WT Orai1 and is purely speculative at this point. We prefer to not engage in speculative structural insights.

Other points

(9) Figures 1G and 1H. The patient-derived mutant STX11 band runs at approximately 37 kDa rather than the predicted 39.5 kDa. The authors suggest instability or reduced antibody reactivity, but premature translation termination is also a possibility that should be acknowledged.

We have added this point in line 168.

(10) Figure 2B. The traces and current voltage relationships should be rescaled to show the rectification and inactivation profile of the current in cells depleted of STX11.

This has been done and modified in new Figure 3 (Figure 2 of version 1).

While preparing source data files for all figures, we noticed an error in the value of the SE in the STX11-depleted group of old Figure 2C, which has now been corrected. The SE value in the older version was erroneously pasted from an adjacent data column.

Similarly, in old Figure 3 (version 1), new Figure 4C, we noticed that some data points in the STX11 group were pasted twice in the same excel column. These cells were removed and additional cells were analyzed from the same experiment and added to this group. The overall result remains the same but the distribution of data points looks a bit different.

(11) Figure 4B: This experiment should be repeated in cells treated with thapsigargin to deplete intracellular calcium stores, and the extent of colocalization quantified by measuring the Pearson's correlation coefficient.

This has been done. Pearson’s correlation coefficient is included in new Figure 5D.

(12) Figure 4F. Why is there no detectable band in the input lane of the left blot?

Western blots show relative intensities of bands of proteins across lanes. A faint band in the input lane of old Figure 4F suggests that the IP/ co-IP/ pull down was robust. If we increase the exposure, the input band would become stronger but the pull-down band would become over-saturated and the difference in the intensities would not be linear. The faint non-specific bands in other lanes represent a fraction of soluble STX11 that tends to crash out of solution over time and gets spun down with the beads. See lines 535-541 explaining this.

(13) Figure 5. Immunofluorescence data showing the membrane staining of the mutated syntaxin and channel should be included, as well as calcium recordings of cells expressing YFP-CAD with WT and mutated ORAI1.

In version 2 Figure 6A, we have now also shown co-localization of mutant STX11 with Orai1-YFP in resting and store-depleted cells, in addition to WGA. Pearson’s correlation (not shown) did not show any significant difference in the localization of mutant synatxin 11 w.r.t Orai1. Calcium recordings of CAD-induced constitutive calcium influx from wild-type versus mutant Orai1 are now shown in new Figure 6O-P.

(14) Figure 6B. A clear colocalization of CFP-O1 and STIM1-YFP is visible on the images, yet the authors conclude from morphometric analysis that the channel is not recruited into ER-PM clusters. Please show the difference in colocalization quantified by measuring the Pearson's correlation coefficient. Pictures should also be provided with the C-terminally tagged construct.

The quantification of CFP-Orai1 localization inside Stim1-YFP puncta was already shown in old Figure 6E and F. The residence of Orai1 inside STIM1 puncta versus total Orai1 in the PM of STX11-depleted groups was clearly reduced. We have now also shown Pearson’s correlation coefficient for Stim Orai co-localization inside puncta in new Figure 7F.

TIRF microscopy images of C-terminally tagged Orai1 were already included in Supplementary Figure 10. No defect in co-clustering of C-terminally tagged Orai1-YFP and N-terminally tagged CFP-Stim1 was seen and yet SOCE was inhibited. Therefore, we never concluded from Figure 6 that Orai1 and Stim1 fail to co-localize. We said, they fail to form ‘functional’ clusters. We have now moved the representative TIRF images from supplementary figure 10 to the new main Figure 7G. The Pearson’s correlation coefficient for Stim Orai1 co-localization inside puncta is shown in new Figure 7L.

(15) Figure 6E and 6F show the same data.

Figure 6E showed fraction of Orai1 inside Stim1 puncta divided by total Orai1, and 6F showed fraction of Orai1 outside puncta divided by total Orai1. The plots are different but we agree that the data are coming from same cells. We have removed old panel 6F and replaced it with Pearson’s correlation coefficient of Stim1:Orai1 colocalization in puncta in new Figure 7F.

(16) Figure 7G-L. The difference in constitutive calcium fluxes should be confirmed by Manganese quench recordings. The surface expression of the Orai1 mutants should be shown.

We have now shown the quantification of surface expression of Orai1 mutants for each respective mutant in the new Figure 8-figure supplement 3B and 3D. The Orai1 mutants we have used in this paper are well established in the literature, they showed clear surface localization and the differences in calcium influx between Scr and STX11 treated cells upon overexpression of Orai1 mutants in HEK are robust. Therefore, we do not see any compelling reason for repeating all of the experiments from Figure 7G to 7L to also show manganese quench recordings, as suggested by the reviewer. We have applied Fura 2 calibration done for these experiments to calculate intracellular calcium. These have been shown in the revised and new Figure 8G-L, where F340/380 ratios of representative calcium assays have also been replaced with the calibrated intracellular calcium concentration.

(17) Supplementary Figure 12. The recordings show a very large variability between experiments. The different SNAREs that are depleted here could compensate for each other, accounting for this variability. It would be interesting to show the effect of the combined silencing of all the SNARES tested here. The efficiency of the protein depletion should also be documented.

Genome-wide high- or medium-throughput screens are inherently noisy. None of the genome-wide high- or medium-throughput screens show evidence of protein depletion for each gene in any of the published screens to our knowledge. We chose to only characterize the candidates that reproducibly showed > 70% inhibition of SOCE, others were ignored as noise.

Silencing of all SNAREs together will definitely lead to loss of morphology and early lethality as all membrane trafficking will be stopped. We never analyze cells that do not show normal morphology and have compromised viability for ablation of SOCE.

(18) Lines 236-238. The authors note that STX11 harbors a stretch of C-terminal cysteines proposed to be essential for its membrane localization, but do not elaborate on the underlying mechanism. It would strengthen the discussion to explicitly acknowledge that this membrane anchoring is mediated by S-acylation of these cysteines PMID: 24910990 and to connect this to the known enrichment of Orai1 in lipid rafts and the immune synapse PMID 34913437. Both observations are relevant to understanding how STX11 and Orai1 are brought into proximity at the plasma membrane, and their omission leaves an explanatory gap in the proposed interaction model.

Please see our response to point #2 above. We do not think C-terminal cysteines target STX11 to the PM. We have corrected this claim based on an earlier study, PMID: 24910990, in the revised version of this paper. Analysis of immune synapse and lipid rafts are outside the scope of this paper. The mechanism of PM targeting of STX11 is currently unestablished and will require a systematic and focused mutational analysis which is outside the scope and main focus of this paper.

(19) Line 351. The statement that syntaxin depletion does not alter the structure or proximity of junctional ER to the plasma membrane is not supported by data. Neither electron microscopy nor TIRF imaging has been performed, which would be required to back up this claim.

Because Stim1 itself can be used as a marker of ER-PM junctions, this statement was supported by data shown in Figure 6C, D, G, H of version 1 of this paper where the intensity and area of Stim1 clusters was assessed using TIRF microscopy and found to be indistinguishable between STX11 and scramble control cells. The imaging done in Figure 6G, H was TIRF imaging and this was already specified in the legend. We have now also done TIRF imaging of GFP-Mapper-expressing scr and STX11-depleted cells. Mapper is a genetically encoded fluorescent protein that was previously shown to mark ER-PM junctions (10). We found no significant difference in the area or intensity of GFP-Mapper puncta (new Figure 7O-Q), just like Stim1 puncta didn’t show any defect in STX11-depleted cells. Please see modified text from 416-423.

(20) The molecular dynamics methods need more detail: force field, simulation length, water box dimensions, and convergence criteria should all be specified to allow replication. The supplementary RMSD plots (Supplementary Figure 5B) should also show individual replicate trajectories rather than averages only.

We had already mentioned the force field (OPLS4) and simulation length (500ns) in the methods section. Also, the RMSD plots in Supplementary Figure 5B already showed individual replicates in version 1.

We have now updated the methods with following additions:

The OPLS4 force field was used for all 500 ns simulations in an orthorhombic water box with a buffer distance of 10 Å beyond the solute in each direction. Simulation stability was assessed based on the protein backbone RMSD over simulation time.

Trajectory clustering was performed using the trajectory clustering tool in Schrödinger, which applies affinity propagation to the pairwise backbone RMSD-based similarity matrix. Within each affinity propagation run, convergence was defined as no change in the set of exemplar frames for 15 consecutive iterations, with a maximum of 400 iterations per run. If convergence was not reached, the damping factor was increased from 0.5 in increments of 0.01 until convergence.

**Reviewer #2 (Recommendations for the authors):**

Overall, this is a timely and impactful study supported by a broad set of methods and cell types. Before publication, the manuscript should address the following points.

Major:

(1) The authors note that STX11 contains cysteine residues that enable membrane association. What is the specific mechanism of membrane attachment? Could it occur via S-acylation (palmitoylation)? Both Orai1 and STIM1 are known to undergo S-acylation, which raises the possibility that this modification might also facilitate STX11 membrane anchoring and/or co-residence with Orai1. Is STX11 constitutively membrane-associated, or does it show preferential localization to specific membrane subdomains, particularly in proximity to Orai1?

STX11 is constitutively membrane-associated and does not show any preferential localization to specific membrane subdomains in confocal images. Figure 4, panel A and B from version1 clearly showed this. In an earlier paper by Hellewell et al. 2014, PMID: 24910990, S-acylation of terminal cysteines of STX-11 was proposed to be crucial for membrane attachment of STX11 and its recruitment to the immune synapse. However, please see our response to reviewer 1’s comment #2 and a new Figure 5E for the localization of the frameshift FHLH4 mutant characterized in this paper. The frameshift mutant that we have characterized lacked all terminal cysteines as well as a short terminal part of the SNARE domain. Cloning and ectopic expression of this mutant still showed constitutive localization to PM and did not show preferential distribution to any specific regions. Therefore, we do not think that terminal cysteines of STX11 contribute to its membrane attachment, we have accordingly modified lines 291-293, 302-305, 540 in the revised version. Also see our response to your point#6 below.

(2) Is there a possibility to monitor a dynamic change in STX11 co-localization from before to after store-depletion?

We did not observe any change in the overall distribution of STX11 in cells expressing STX11 alone or co-expressing Orai1 with STX11, pre- or post-store-depletion (please see new figure 5B-C). In cells co-expressing ORAI1, STX11 and STIM1 (see new Figure 5M-N), we could not capture the dynamic segregation of STX11 into regions of PM devoid of STIM:ORAI puncta and therefore have only pre- or post-store-depletion images. Dynamic change in STX11 distribution would require live, multi-colour, high-resolution imaging of diffraction-limited ER-PM junctions and adjacent regions which is technically extremely challenging, and especially due to our inability to tag STX11 with a fluorescent tag without disrupting its localization. Also see our response to your point#6 below.

(3) The authors use CAD to prove that the interaction with the R289A_E272A_E275A_E278A mutant is normal as for the wild-type. Does this also hold for STIM1 wild-type full-length?

This is also true for full-length STIM1. The data have now been added to the new Figure 7-figure supplement1.

(4) The authors state that STIM1 binds both the N- and C-termini of Orai1. While STIM1 binding to the Orai1 C-terminus is well established, the nature of its interaction with the N-terminus remains debated. Fragment-based assays suggest direct binding to the N-terminus; however, direct interaction with full-length Orai1 has not been conclusively demonstrated. This point should be phrased more cautiously to reflect the current uncertainty.

We have re-phrased the sentence as follows in line 543: “The individual relevance of Orai1 N- versus C-terminus in the trapping versus gating of Orai1 remains unclear”

(4) In the discussion, the authors report: "Though crucial for trapping and gating, Orai1 tails were missing from early structures of Drosophila Orai [28]". A previous NMR structure suggested that the C-terminal tails of two adjacent Orai1 subunits bend and pair with each other in an antiparallel fashion, and sit closely apposed to PM [37]. However, in recent structures of constitutively active H134 mutant Orai, the C-terminal tails were found to orient away from the membrane [28]. In STX11-depleted cells, switching the CFP-tag from the Orai1 N- to the C-terminus could rescue its clustering but not gating by Stim1. Furthermore, STX11 depletion inhibited the constitutively active ANSGA mutant of Orai1 [29], where the tails of Orai1 are proposed to be constitutively unlatched. These data essentially reinforce our conclusions that STX11 induced molecular shifts encompass Orai1 transmembranes.' However, the information provided here is not fully correct. The early Drosophila Orai structure lacks the full N-terminus but retains most of the C-terminus. It was the X‑ray, not cryo‑EM, structure that suggested an antiparallel arrangement of the Orai1 C-termini. Although the "open" X‑ray structure shows unlatching and straightening of TM4-C-termini, it remains uncertain whether these features reflect physiological gating or crystallization artifacts. It is also unclear whether the Orai1 ANSGA gain‑of‑function mutant adopts a similar unlatching; however, prior work indicates that ANSGA impairs proper coupling to the C‑terminal binding interface (in contrast to Orai1 H134S, which maintains effective coupling). This raises the key question: why do H134S and ANSGA respond differently to STX11 depletion? One possibility is that these mutants stabilize distinct conformations of the TM4-C-termini ("latched" vs "unlatched" states) that differentially dictate the requirement for STX11 in channel assembly or gating. We recommend refining the discussion

We have changed the word ‘missing’ to ‘truncated’ in line 545 and 546.

We agree that there is no evidence in literature that establishes similarity between H134S and ANSGA mutation-induced conformations of Orai1. It is, however, implied in most previous studies of mutant Orai1s that there is only one possible open state/conformation. We have added the suggested point and modified the discussion in line 551-556.

(5) The authors highlight: 'A major problem with this interpretation is that even though amplification of CRAC currents was shown, none of the previous patch clamp studies established whether the higher currents resulted from a greater number of active channels or unchecked conductance per channel by performing single channel recordings.' It should be noted that CRAC channels have extremely low single‑channel conductance, making direct single‑channel recordings challenging. As a result, estimates of open probability and channel number typically rely on fluctuation (noise) analysis rather than direct measurements of single‑channel events (see https://doi.org/10.1085/jgp.200609588). We suggest acknowledging this limitation in the discussion to contextualize the interpretation of gating and channel density.

We acknowledge how challenging it is to record the single-channel conductance from CRAC channels. We have added this fact to the discussion and the reference that the reviewer has suggested in line 570-572.

(6) STX11 appears to shift Orai1 localization into puncta. Activated STIM1 is known to engage plasma membrane PIP2 to facilitate Orai1 coupling. How, if at all, is STX11 linked to PIP2 or PIP2-rich microdomains? Is there evidence for direct PIP2 binding by STX11, or for indirect recruitment via PIP2-binding partners? Any available data on STX11's lipid interactions or its enrichment within PIP2-enriched regions would help clarify this mechanism.

We have not claimed that STX11 shifts Orai1 into puncta. We already showed in old supplementary figure 10 and Figure 6G-J of version1 (v1) of this paper that the C-terminally tagged Orai1-CFP can very well form puncta and co-localize with STIM1 in STX11-depleted cells. To avoid this confusion, we have moved the old Supplementary Figure 10 from v1 to the main figure in revised version, see new Figure 7 panel G. Despite the presence of ORAI1-CFP in puncta with YFP-Stim1, the SOCE was inhibited in STX11-depleted cells. Please also see new Pearson’s correlation coefficient for Stim1 and Orai1 colocalization in Figure 7 panel L. Therefore we concluded that, Orai1 forms ‘nonfunctional’ clusters with Stim1 in STX11 depleted cells, please see modified lines 408-412, clearly explaining this.

Although syntaxin 1A has been shown to interact with cholesterol (11) as well as PIP2 (7, 8) using either a stretch of polybasic residues or basic residues spread throughout several domains. To our knowledge, these have not been proposed to recruit or segregate STX11 in membranes. STX11 doesn’t contain an obvious stretch of poly-basic residues in its sequence, either, to quickly mutate and address this question. Please also see our response to your point #1 and #2 above. Answering this question will require a systematic and dedicated mutagenesis study.

Minor:

(1) Please indicate in Figure 1 in the respective graphs in which cell type the Ca2+ imaging studies have been performed.

Done.

(2) Figure 4C, D: Why are the input bands so weak?

Please see our response to reviewer #1’s similar comment 12 above.

(3) Figure 5A: Please clarify what WGA is.

WGA is wheat germ agglutinin which is used to mark PM in imaging experiments. It binds to N-acetyl-D-glucosamine and sialic acid residues found in mammalian cell membranes and glycoproteins. We have added the explanation to the new Figure 6A legend.

The authors state that "the constitutively active ANSGA (261-265) mutant of Orai1 (Supplementary Figure 11G), which harbors 4 consecutive mutations in the Orai1 C-terminus ...". Please clearly state this is the nexus region connecting the C-terminus with TM4. The 5 aa stretch is not the C-terminus; it is just close to the C-terminus.

We have modified this, as suggested, in line 470-471.

References:

(1) Miao Y, Miner C, Zhang L, Hanson PI, Dani A, Vig M. An essential and NSF independent role for alpha-SNAP in store-operated calcium entry. Elife. 2013;2:e00802.

(2) Li P, Miao Y, Dani A, Vig M. alpha-SNAP regulates dynamic, on-site assembly and calcium selectivity of Orai1 channels. Mol Biol Cell. 2016;27(16):2542-53.

(3) Chorev DS, Baker LA, Wu D, Beilsten-Edmands V, Rouse SL, Zeev-Ben-Mordehai T, et al. Protein assemblies ejected directly from native membranes yield complexes for mass spectrometry. Science. 2018;362(6416):829-34.

(4) Dorwart MR, Wray R, Brautigam CA, Jiang Y, Blount P. *S. aureus* MscL is a pentamer in vivo but of variable stoichiometries in vitro: implications for detergent-solubilized membrane proteins. PLoS Biol. 2010;8(12):e1000555.

(5) Vig M, Peinelt C, Beck A, Koomoa DL, Rabah D, Koblan-Huberson M, et al. CRACM1 is a plasma membrane protein essential for store-operated Ca2+ entry. Science. 2006;312(5777):1220-3.

(6) Feske S, Gwack Y, Prakriya M, Srikanth S, Puppel SH, Tanasa B, et al. A mutation in Orai1 causes immune deficiency by abrogating CRAC channel function. Nature. 2006;441(7090):179-85.

(7) Murray DH, Tamm LK. Clustering of syntaxin-1A in model membranes is modulated by phosphatidylinositol 4,5-bisphosphate and cholesterol. Biochemistry. 2009;48(21):4617-25.

(8) van den Bogaart G, Meyenberg K, Risselada HJ, Amin H, Willig KI, Hubrich BE, et al. Membrane protein sequestering by ionic protein-lipid interactions. Nature. 2011;479(7374):552-5.

(9) Miranda P, Contreras JE, Plested AJ, Sigworth FJ, Holmgren M, Giraldez T. State-dependent FRET reports calcium- and voltage-dependent gating-ring motions in BK channels. Proc Natl Acad Sci U S A. 2013;110(13):5217-22.

(10) Chang CL, Chen YJ, Liou J. ER-plasma membrane junctions: Why and how do we study them? Biochim Biophys Acta Mol Cell Res. 2017;1864(9):1494-506.

(11) Lang T, Bruns D, Wenzel D, Riedel D, Holroyd P, Thiele C, et al. SNAREs are concentrated in cholesterol-dependent clusters that define docking and fusion sites for exocytosis. EMBO J. 2001;20(9):2202-13.
